# Solvent-Fueled Anion Translocation in Crystalline Phosphonium Salt

**DOI:** 10.1021/jacs.6c12003

**Published:** 2026-09-16

**Authors:** Eetu Hakkarainen, Pei-Ying Huang, Chen-Yu Lin, Pi-Tai Chou, Igor O. Koshevoy, Glib V. Baryshnikov, Andrey Belyaev

**Affiliations:** † Department of Chemistry and Sustainable Technology, 163043 University of Eastern Finland, Joensuu 80101, Finland; ‡ Department of Chemistry, 33561 National Taiwan University, Taipei, Taiwan 10617, Republic of China; § Laboratory of Organic Electronics, Department of Science and Technology, 4566 Linköping University, Norrköping 60174, Sweden

## Abstract

Externally stimulated ion motion within rigid crystalline matrices has significant potential to tune the functional properties of bulk materials but is difficult to control. Here, we report a diphosphonium diiodide that allows facile solid-state solvent-driven anion translocation, providing a direct optical readout of ionic motion. The dynamic character of anion−π^+^ interactions enables reversible reorganization of ion pairs via exchange of co-crystallized solvent. The relocation of the iodides over the distance of 4.3 Å triggers phase transitions and transforms the configuration of the excited state, thereby switching among locally excited fluorescence (^1^LE), locally excited phosphorescence (^3^LE), and charge-transfer phosphorescence (^3^CT). Consequently, photoluminescence is modulated across an exceptionally wide spectral window from 415 to 650 nm. Structural and theoretical investigations reveal that noncovalent interactions with solvent molecules dictate the delicate balance between solvation, hydrogen bonding, and anion−π^+^ contacts. The resulting ion-migration-driven phase dynamics enable the conversion of directional solid-state motion into programmable luminescent responses, offering a new platform for sensing, information storage, and adaptive optoelectronic technologies.

## Graphical abstract

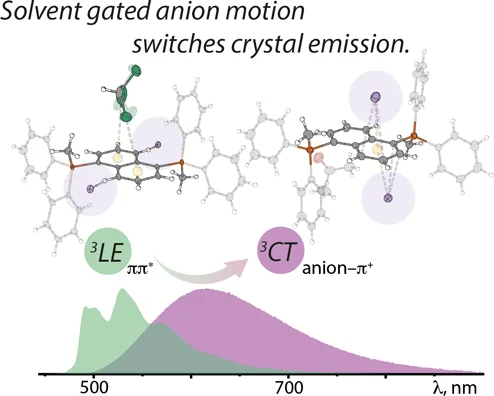

## Introduction

The translation of programmed molecular motion into functional materials represents a central objective in modern chemistry.^1^ Incorporation of dynamic units within hierarchical architectures allows nanoscale motion to be amplified into macroscopic responses, which find applications in soft robotics, adaptive actuators, self-healing systems, artificial muscles, energy transduction platforms, dynamic luminescent materials, and many more.^2–7^ While solution-phase, polymeric systems and liquid crystals readily undergo structural rearrangements, rigid crystalline solids impose strict constraints through dense packing and extended networks of non-covalent interactions. Rather than merely limiting mobility, such confinement can encode cooperative and anisotropic motion within the lattice, enabling crystalline actuators, photomechanical materials, and responsive frameworks.^1,8–10^ Crystalline molecular machines incorporating rotator–stator architectures and free-cavity-engineered lattices have demonstrated persistent motion while preserving long-range order.^11–14^ In parallel, coupling solid-state motion to optical output has emerged as a powerful strategy toward sensing materials: reversible switching of luminescence via the perturbation of π–π interactions,^15^ conformational isomerism as a result of single-crystal-to-single-crystal transformations,^16–18^ and solvent- or mechanically induced time-dependent photoemission responses in host–guest (co)­crystals^6,19–23^ illustrate how structural dynamics can be translated into enhanced luminescence and/or tunable color switching.^24^ Reversibility, regulation and predictability of these motion processes are critical for practical applications, as multiple, fatigue-resistant cycles of structural and emissive switching are required for adaptive materials, encryption, solid-state sensors, and information storage systems.^25–27^ Nevertheless, controlled ionic motion, particularly directional cation/anion translocation, within rigid crystalline materials, remains essentially unexplored.^28^ Importantly, a solid-state platform in which stimulus-driven ion migration directly governs excited-state, e.g., fluorescence versus phosphorescence pathways, has not yet been realized.

Herein, we report the first artificial molecular material demonstrating controllable and reversible anion motion within a crystalline environment driven by guest solvent. The anion translocation reorganizes anion (I^–^)−π (diphosphonium)^+^ pair that changes the configuration of the excited state and the emission characteristics. The observed solvent-fueled ion motion establishes a structure–function platform to control competing noncovalent interactions, such as Coulombic attraction, hydrogen bonding, anion−π^+^ interactions, and solvent–chromophore coupling, thereby modulating anion–cation communication within the lattice. As a result, the radiative decay of the crystalline material can be selectively directed to either fluorescence (^1^LE) or phosphorescence (^3^LE or ^3^CT) channels. In contrast to previously reported luminescent ionic contact pairs,^29,30^ which were investigated in the solid state as static structures and photoemissions, this new system introduces a novel design principle, where ionic motion regulates the on–off modes of anion−π^+^ interactions directly coupled to a distinct photophysical response.

## Result and Discussion

### Synthesis

The diphosphonium diiodide **1** (Figure 1) was readily obtained as a pale-yellow microcrystalline solid in nearly quantitative yield by the alkylation of 1,5-bis­(diphenylphosphino)­naphthalene (**a1**) with an excess of methyl iodide in chloroform (see Supporting Information for experimental details). The structure and integrity of the product were confirmed by high-resolution mass spectrometry (HRMS), and ^1^H, ^13^C­{^1^H}, ^31^P­{^1^H} NMR spectroscopy (Figures S1–S10). The resonance at 23.9 ppm in the ^31^P­{^1^H} spectrum agrees with reported values for related tetraarylphosphonium congeners.^29,31^ Elemental analysis and high-performance liquid chromatography (Figure S11) confirmed its analytical purity.

**Figure 1:**
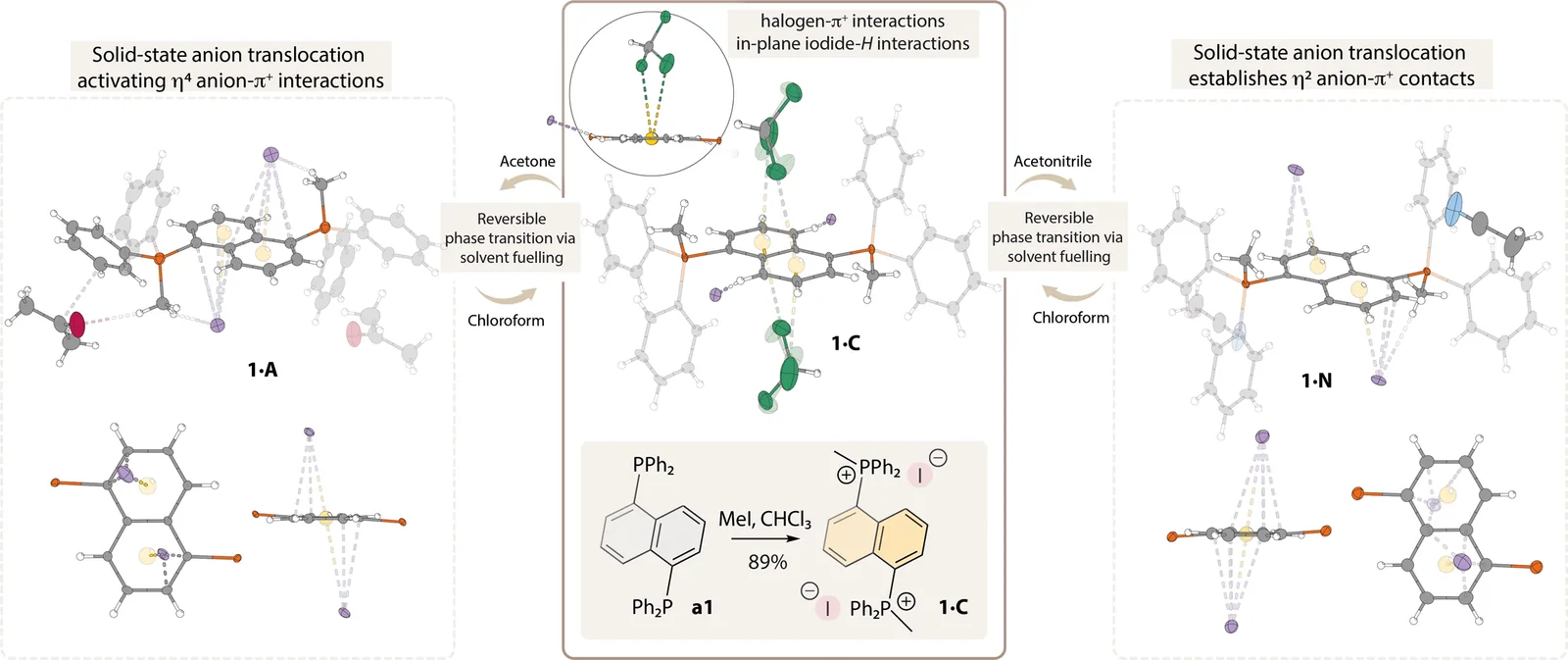
Fragments of crystal structures of solvated forms **1·C**, **1·N,** and **1·A** with the shortest halogen-π^+^, anion-π^+^, and I-centroid­(π^+^) interactions (150 K; displacement ellipsoids are shown at the 50% probability level) and solvent-induced interconnecting phase transitions. Inset shows synthetic access to **1·C**.

### Solid-State Anion Relocation

Single crystals of the chloroform solvate **1·C** (*P*2_1_/*c* space group) were readily obtained from the corresponding solvent (Figure 1 and Table S1). Unlike previously reported diphosphonium salts,^29^ no anion (I^–^)−π­(diphosphonium)^+^ interactions are observed in **1·C**, having a nearly in-plane (I^–^)–naphthalene configuration of the ion pair (Figure 1). Instead, the electron-deficient polyaromatic core is encapsulated by chloroform molecules through multiple C–Cl···π^+^ contacts, with Cl···centroid separations of 3.55–4.55 Å. These halogen−π^+^ interactions dominate the supramolecular organization, shielding the cation-decorated π-surface and suppressing soft anion−π^+^ bonding. The chloroform molecules further self-assemble along the *c*-axis into infinite one-dimensional chains sustained by a cooperative network of C–H···I (2.65–3.11 Å) hydrogen and Cl···Cl (3.68–3.82 Å)/Cl···I (3.68 Å) halogen bonds (Figures S12–S14).^32^ This solvent-supported architecture defines continuous void regions lined by the cationic framework as pre-organized pathways for possible guest exchange. Indeed, upon exposure to drops of acetonitrile or its vapors, microcrystalline **1·C** instantly transforms into the modification **1·N** (*P*1̅), as confirmed by SCXRD and PXRD (Figures 1, 2, S14, S15 and Table S1). The phase transition presumably proceeds via solvent substitution within existing voids and is accompanied by the migration of the iodide counterions (Figure 2). This adaptive reorganization of the ion pair gives rise to direct anion−π^+^ interactions manifested by two symmetry-related η^2^-type I^–^–π­(diphosphonium)^+^ (*d*(C···I) = 3.95/4.01 Å) and I···centroid contacts of 3.99 Å. The observed arrangement is apparently stabilized by C–H···I hydrogen bonding with aromatic and methyl protons. Similarly, the treatment of **1·C** with a few drops or fumes of acetone produces solvate **1·A** (*P*2_1_/*n*) featuring a pair of symmetrical trans-oriented anion­(I^–^)−π^+^ interactions over each six-membered ring of the naphthalene bearing a phosphonium group (Figures 1 and S15). The anion−π^+^ interactions in **1·A** can be described as distorted η^4^-type (*d*(C···I) = 4.02/4.06/4.23/4.27 Å) with I···centroid contacts, which are comparable to the distances found in **1·N** (4.00 Å). Acetone molecules intercalate between the phenyl rings of the diphosphonium dications, generating solvent channels within columns stabilized by π–π interactions with an interplanar distance of 3.67 Å (Figures S16 and S17).

**Figure 2:**
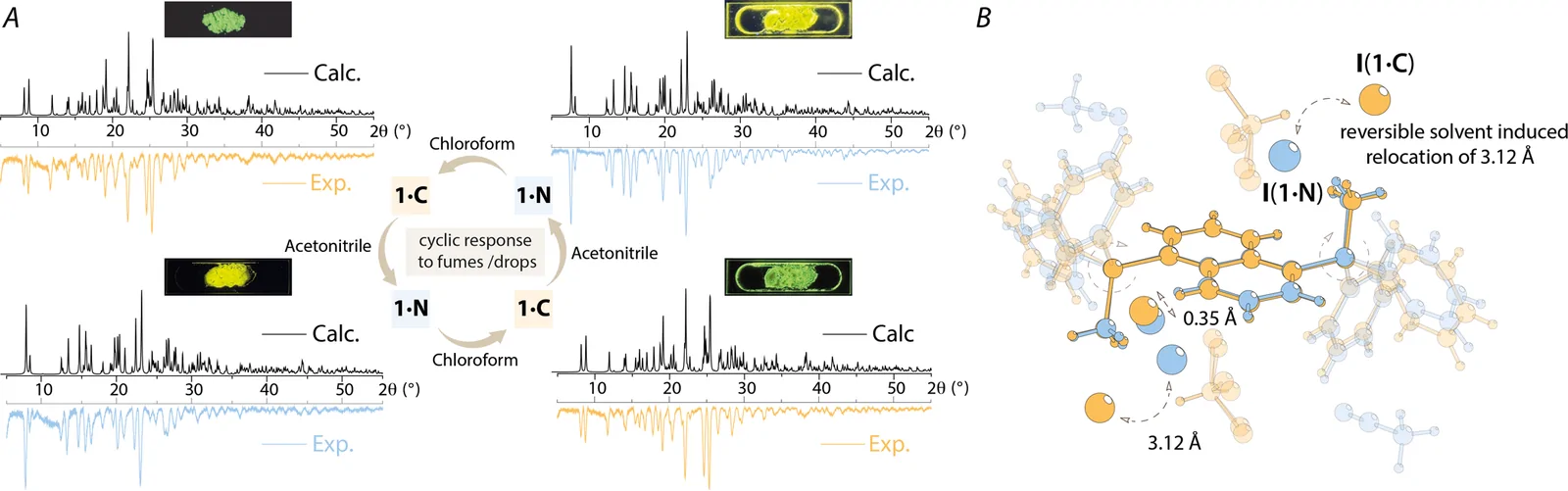
(A) PXRD data of the cyclic phase transition **1·C** ↔ **1·N** showing fully reversible crystalline transformation (insets show material upon irradiation with 365 nm); (B) overlay of the fragments of crystalline structures of **1·C** (yellow) and **1·N** (blue) fixed at naphthalene carbon atoms demonstrating the distance trajectory of anion­(I^–^) translocation in the solid state (ball and stick representation, shortest pairs of I^**1·C**^···I^**1·N**^ are shown).

Transitions **1·C** ↔ **1·A**, **1·C** ↔ **1·N** and **1·N** ↔ **1·A** are fully reversible upon exposure to either the vapors or droplets of the corresponding solvent (Figures 2 and S15). The longest path of anion translocation between two positions around the cationic core is calculated to be approximately 3.12–3.37 Å (Figures 2 and S18). This motion is coupled with positional adjustments of the −PPh_2_Me fragments, which undergo rotational motions relative to the naphthalene platform acting as a stator. It is accompanied by a substantial sharpening of the corresponding torsion angle between the naphthalene plane and the P–C_Me_ direction (ψ = 61.66° to 55.66°/60.72°) and the concomitant reorientation of the C_Me_–H···I bonding. Altogether, these changes demonstrate that the iodide migration is not a simple translation but a mechanically correlated process (Figure S19), in which the counterion and rotor move in concert. It also differs from the work of Gong, Sessler and Tang,^19^ in which solid-state phase transition proceeds via host–guest molecular motion within minutes to hours: the transformations **1·A** ↔ **1·C** ↔ **1·N** occur almost instantaneously upon solvent fueling (Supporting Information Videos S1, S2, S3 and S4). In the **1·C** phase, however, the iodide anion, despite being engaged in secondary contacts, is spatially segregated from the cationic π-surface by intercalated chloroform molecules, which prevent direct anion−π^+^ interaction. The C–H···I and C–Cl···I contacts under these conditions are insufficient to enforce intimate ion pairing at the electron-deficient aromatic core. The substitution of guest chloroform molecules with acetone or acetonitrile, both of which lack sufficiently acidic protons to form H-bonding with iodides, effectively removes these competing interactions involving solvents. The elimination of this solvation barrier enables the counterion to approach the π^+^ surface, where anion−π^+^ interactions become energetically dominant and dictate the reorganization of the ion pair. The phase transformation can thus be understood as an adaptive supramolecular reconfiguration in the solid state based on the solvent-gated rotor–anion coupled process.

Interestingly, the recrystallization of **1** at 297 K from acetone afforded two additional solvatomorphs besides the major **1·A^**o**^** phase, namely the minor **1·A** and traces of **1·A** ^**g**^ (Figures 3, S20 and Tables S1–S3). Short-time boiling of **1·A** in dry acetone (2–4 min, under an inert atmosphere) selectively yields the thermodynamically unfavored **1·A** ^**o**^ phase. SCXRD revealed that in **1·A** ^**o**^ (*Pbca*) the iodide anions sandwich the same six-membered ring of the naphthalene, engaging in η^1^ and η^2^-type anion−π^+^ interactions. The conversion of **1·A** to **1·A** ^**o**^ occurs only upon heating, indicating a thermally induced structural reorganization. Full-energy calculations of quantitatively equivalent **1·A**/**1·A** ^**o**^ structural models consistently favor the **1·A** configuration, with the calculated total energy of **1·A** being lower than that of **1·A** ^**o**^ by 140, 112, and 120 kcal mol^–1^ for the solvated (**1** + 2I^–^ + acetone), desolvated (**1** + 2I^–^), and cation-iodide−π^+^ (**1**) models, respectively. The persistence of this energetic ordering upon removal of acetone and simplification of the iodide environment supports an intrinsic energetic preference for the **1·A** anion configuration and is consistent with the experimentally observed thermally induced conversion to **1·A** ^**o**^. The process requires a longer relocation path for the iodide, combined with conformational reorientation of the −PPh_2_Me groups and partial disruption of already formed anion−π^+^ contacts, resulting in a higher energetic barrier and consequently slower transformation. Above agrees with the results of QTAIM analysis (Figure S21 and Table S4), indicating that for one of the iodides, the binding energy moderately changes upon conversion of **1·A** to **1·A** ^**o**^ (−3.26 vs −4.24 kcal mol^–1^, respectively), while for the second iodide the relocation is considerably endothermic (binding energy decreases from −3.26 to only −1.00 kcal mol^–1^, respectively). Point contacts in **1·A** ^**o**^ are slightly elongated *d*(C···I) = 4.18 Å relative to the η^2^-interaction (4.04/4.06 Å), indicating that the solvation of the anion by acetone induces asymmetric binding at the π^+^ surface. In contrast, the trace metastable **1·A** ^**g**^ (*Pn*) phase displays no anion−π^+^ interactions, with the closest iodide-naphthalene core distance of 3.92 Å (Figure S20). Instead, acetone molecules occupy the space above/below naphthalene and stabilize the structure through complementary C–H­(acetone)···π^+^ interactions (2.71–3.18 Å), competing with the counterion for access to the electron-deficient aromatic surface.

**Figure 3:**
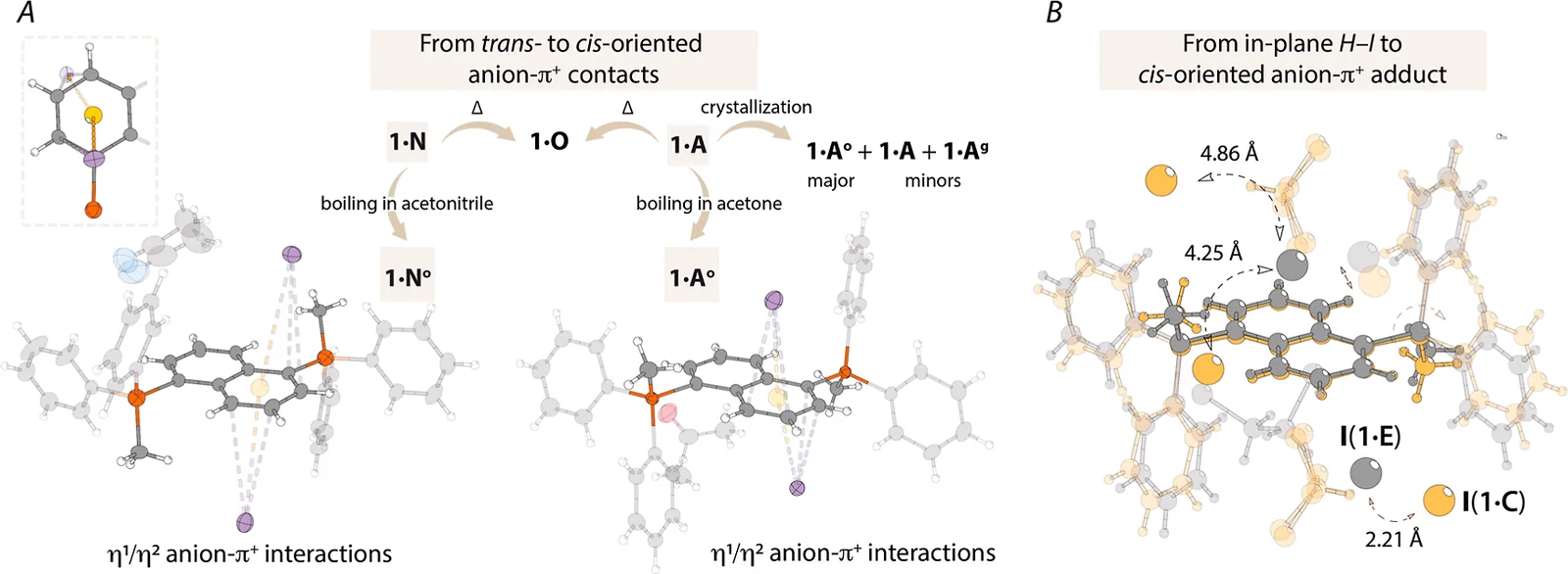
(A) Fragments of crystal structures of solvated forms **1·N** ^**o**^ and **1·A** ^**o**^ with the shortest anion−π^+^, and I-centroid­(π^+^) interactions (150 K; displacement ellipsoids are shown at the 50% probability level) and a scheme interconnecting possible phase transitions. (B) Overlay of the fragments of crystalline structures of **1·C** (yellow) and **1·E** (grey) fixed at naphthalene carbon atoms demonstrating the distance trajectory of anion­(I^–^) translocation in the solid state (ball and stick representation).

Boiling **1·N** in dry acetonitrile (5 min, inert atmosphere) also transforms the phase into solvate **1·N** ^**o**^ (*Pbca*), which is isomorphous to **1·A** ^**o**^ and displays similar *cis*-anion−π^+^ arrangement around a single naphthalene six-membered ring in an I^–^–C_6_H_4(napht)_–I^–^ fashion, formed via an asymmetrical pair of elongated η^1^ and η^2^ anion−π^+^ contacts (*d*(C···I) = 4.26 and 4.13/4.16 Å) (Figure 3). One iodide is laterally displaced relative to the ring centroid and positioned off-axis toward the π surface, indicative of a distorted anion−π^+^ interaction geometry. Unfortunately, the crystalline **1·N** ^**o**^ can be obtained only with some admixture of **1·N** microcrystals, probably due to a small energy difference between **1·N** and **1·N** ^**o**^ phases and an easy interconversion of **1·N** ^**o**^ ↔ **1·N**. Indeed, as follows from the QTAIM analysis (Figure S22 and Table S5) the total binding energy of iodides in **1·N** and **1·N** ^**o**^ is almost the same (−6.33 vs −5.46 kcal mol^–1^, respectively), and translocation of iodides during the transition **1·N** → **1·N** ^**o**^ compensates each other energetically, i.e., exothermic for one iodide (−3.17 vs −4.17 kcal mol^–1^, respectively) and endothermic for the second one (−3.17 vs −1.29 kcal mol^–1^, respectively).

The *cis*-oriented anion−π^+^ arrangement is achieved within minutes directly from the in-plane solvatomorph upon soaking **1·C** in 1,2-dichloroethane, affording the **1·E** phase isomorphous to **1·A** ^**o**^ and **1·N** ^**o**^ (Figures 3, S23 and Table S2). This irreversible transformation proceeds via asymmetric iodide relocation without additional energy input and involves one significantly longer migration pathway (ca. 4.25 Å) compared to the **1·C** ↔ **1·A**/**1·N** interconversion (Figures 1, 2, S15 and S18). The solvent confined in the lattice voids plays a decisive role by balancing steric constraints engaging secondary interactions. For instance, in **1·E**, 1,2-dichloroethane stabilizes the anion through Cl···I halogen bonding (3.68 Å), which competes with anion−π^+^ binding and partially retains the iodide in a solvent-stabilized environment. As a result, anion−π^+^ interactions are attenuated and expressed as one η^2^-interaction (4.05/4.10 Å) and another elongated weak η^1^ contact (*d*(C···I) ≈ 4.40 Å), highlighting the possibility of the soft-tunable solvent-dependent modulation of cation–anion communication in **1·A** ^**o**^/**1·N** ^**o**^/**1·E**.

Further theoretical analysis (Figure S24 and Table S6) revealed that the overall binding energy in **1·C** is greater (−16.6 kcal mol^–1^) than in **1·E** (−11.0 kcal mol^–1^). However, a substantial fraction of the stabilization in **1·C** arises from I···HCCl_3_ interactions, contributing ca. −3.3 kcal mol^–1^ per iodide. To assess the intrinsic strength of iodide pairing within the crystal lattices, we estimated the effective iodide–cation binding energies after excluding the contribution from solvent self-association. Under these conditions, the iodides interact with cations at virtually the same energies in **1·C** (−3.3 kcal mol^–1^) and **1·E** (−3.5 kcal mol^–1^). This rationalizes the facile replacement of chloroform by 1,2-dichloroethane, following conversion of the **1·C** phase into **1·E** without thermal activation. In contrast, the binding energy of the two *cis*-oriented iodides in **1·E** amounts to −6.94 kcal mol^–1^, which substantially exceeds the corresponding values computed for **1·A** ^**o**^ and **1·N** ^**o**^ (−5.24 and −5.46 kcal mol^–1^, respectively). The stronger iodide pairing in **1·E** therefore provides a plausible energetic rationale for the thermal input required for the transformation of **1·C** into the **1·A** ^**o**^ and **1·N** ^**o**^ phases.

Exposure of all aforementioned phases of **1** to methanol or water also triggers instantaneous phase transitions (Figure 4); the latter accounts for the general aging of all observed solvatomorphs (i.e., solvated molecules are exchanged by water, allowing a phase transition to **1·W**), which occurs within 4–12 h under ambient conditions and depends on crystal size. The single crystals of water (**1·W**) and methanol (**1·M**) solvates exhibit anion–naphthalene arrangements analogous to those observed in **1·C**, with the solvated iodides and solvent molecules stabilizing the dication (Figures 4, S18, S25 and Table S2). Both **1·W** and **1·M** can be reversibly converted to **1·C** upon addition of a few drops of chloroform. Although the geometric parameters required for anion relocation from **1·W**/**1·M** to **1·N**/**1·A** are comparable to those for **1·C** (Figure S18), the strong hydrogen-bonding networks formed by water and methanol likely impose a substantial energetic barrier to solid-state rearrangement. Consequently, fast phase transitions are suppressed, and **1·N**/**1·A** are generated from **1·W**/**1·M** only via slow dissolution–recrystallization pathways.

**Figure 4:**
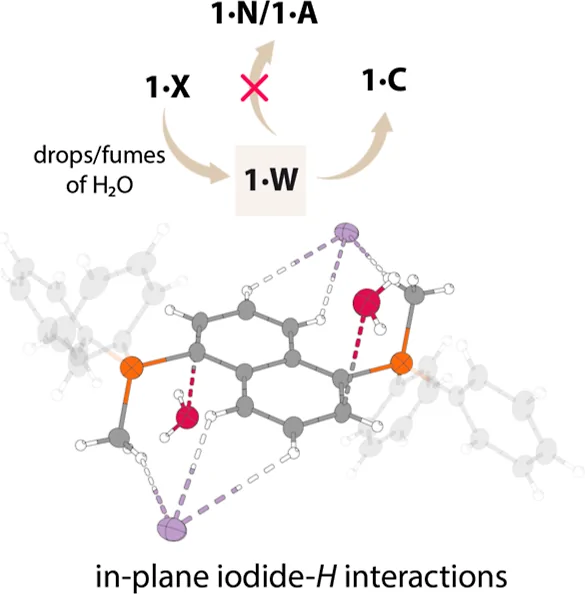
SCXRD structure representation of the water-solvated form **1·W** that occurs upon aging of **1·X** (**X** = **C**, **N**, **A**, **N** ^**o**^, **A** ^**o**^) in the air or direct water exposure.

The phases were characterized by thermogravimetric analysis (TGA) and differential scanning calorimetry (DSC), which demonstrate the high thermal robustness of the salt framework up to ca. 620–670 K (Figures S26–S28). The TGA traces of the solvates exhibit an initial mass loss accompanied by corresponding DSC signals, attributable to solvent release, which is complete by ca. 370–420 K. This process yields a solvent-free material that subsequently undergoes thermal reorganization and crystallization. At higher temperatures, all investigated solvatomorphs exhibit a small additional mass loss of approximately 2% at ca. 623 K, accompanied by a distinct DSC event, consistent with partial demethylation. At still higher temperatures, further demethylation is obscured by overlapping sublimation of **a1** and overall decomposition processes, preventing its independent quantification. Notably, exothermic DSC events assigned to thermal crystallization at 477–560 K are observed both for the solvated samples and for the pre-dried samples, indicating that solvent removal and subsequent structural reorganization, including partial amorphization, occur through distinct thermal processes. Although these thermal transitions demonstrate the formation of thermally reorganized crystalline materials, their detailed crystal structures cannot be unambiguously established from TGA/DSC data alone. Among the investigated phases, only **1·N** and **1·A** were confirmed to undergo a reproducible transformation to the same amorphous phase at 100 °C, designated as **1·O**, whereas the structural identity of the thermally treated products obtained from the other solvatomorphs remains unresolved (Figure S29).

PXRD analysis further verifies the phase purity of the solvated microcrystalline bulk materials, with experimental patterns in agreement with those simulated from single-crystal data (Figures 2, S15, S29 and S30).

### Optical Response to Anion Relocation

Solvent-mediated anion relocation in **1·C** → **1·A/1·N/1·E** and **1·C** → **1·M** can be directly visualized under UV irradiation (Figures 2A and 5A, see Supporting Information Videos S1, S2, S3, S4, S5 and S6). The parent chloroform solvate **1·C** exhibits bright green luminescence (Q.Y. = 68%) with a well-resolved vibronic emission profile featuring maxima at 491, 500, 528, and 569 nm (Δ*ν* = 1061–1427 cm^–1^, Figure 5A, Tables 1, S7 and S8). The pronounced vibronic progression, with spacings characteristic of intramolecular vibrational modes of the aromatic framework, is consistent with a localized π → π* excitation. The average lifetime of 1881.1 μs at 297 K corresponds to a relatively low radiative rate constant of *k*_r_ = 361 s^–1^. Such rates are commonly associated with ^3^ππ* dominant phosphorescence in purely organic chromophores and are generally substantially lower than those attainable for triplet states with pronounced CT character or enhanced heavy-atom-assisted SOC.^29,33,34^ TDDFT calculations identify the emissive T_1_ state as primarily localized on the polyaromatic fragment, with a ^3^LE configuration contributing >75% (Figures 5B and S31), supporting assignment of the emission to a triplet locally excited (^3^LE, π → π*) state of the naphthalene core. The low *k*_r_, together with the vibronically resolved emission profile and the calculated ^3^LE character, is consistent with phosphorescence from an emissive triplet state possessing substantial ^3^ππ* character. The magnitude of *k*_r_ reflects the overall radiative transition probability of the emissive triplet state, which is governed by its electronic character and by the extent to which the formally spin-forbidden T_1_ → S_0_ transition acquires allowed character through SOC and excited-state mixing.^34^

**Figure 5:**
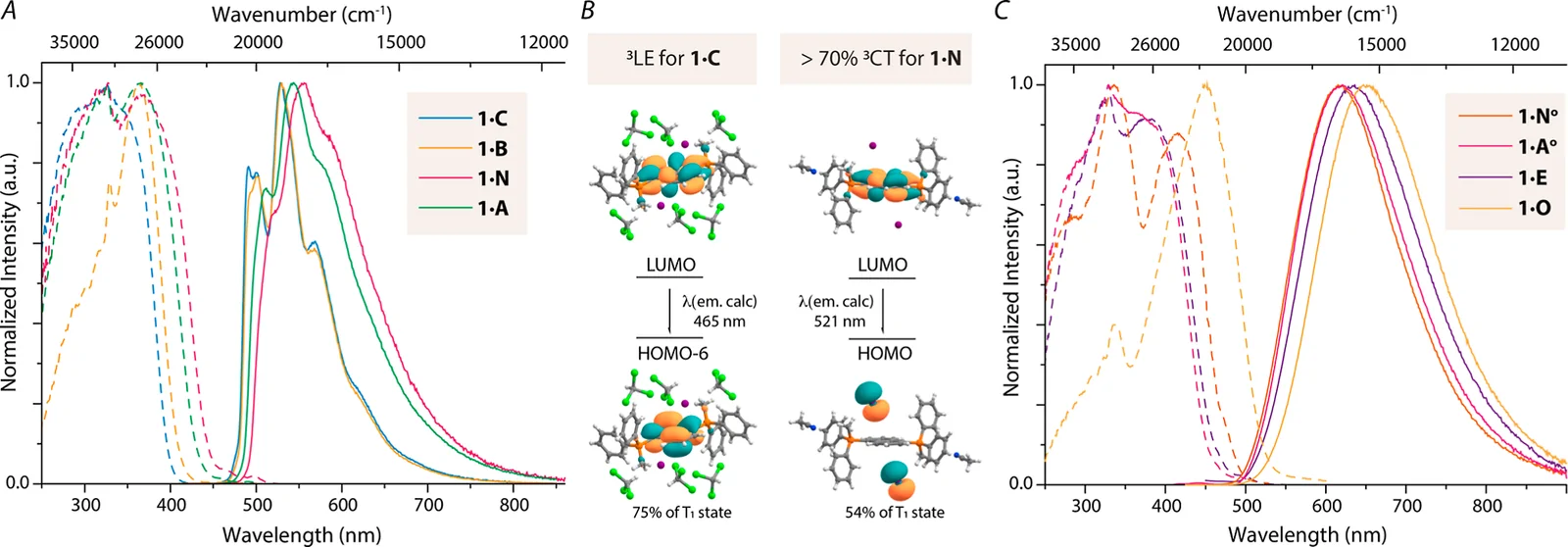
Excitation (dashed) and emission (solid) spectra of (A) **1·C**, **1·B**, **1·N**, **1·A** and (C) **1·N** ^**o**^, **1·A** ^**o**^, **1·E** and **1·O** at 297 K; (B) selected molecular orbitals of **1·C** and **1·N** representing the change of T_1_ composition upon anion relocation.

**Table 1:** Summary of Solid-State Photophysical Properties at 297 and 77 K

	λ_exc_ [nm]	λ_em_ [nm]		τ_av_ [μs]^a^		Q.Y. [%]	*k*_r_ [10^5^ s^–1^]^b^	*k*_nr_ [10^5^ s^–1^]^b^	
	297 K	297 K	77 K	297 K	77 K	297 K	297 K	297 K	excited state at 297 K
**a1**	370	505	500	0.009	0.015	46	511.1	600.0	^1^LE
**1·W**	358	415, 435	498, 519, 524, 538, 564, 610	0.009	779.2	n.d.	n.d.	n.d.	^1^LE
**1·C**	325	491, 500, 528, 569	497, 523, 538, 562	1881.1	2384.2	68	0.0036	0.0017	^3^LE
**1·B**	364	500, 529, 568	501, 526, 570, 615	115.4	1280.3	11	0.0096	0.0772	^3^LE
**1·N**	370	521, 551, 584	501, 512, 541, 550, 584, 633	22.8	89.8	23	0.1008	0.3372	^3^LE + CT
**1·A**	366	511, 544, 577	492, 509, 533, 580	23.0	138.7	13	0.0565	0.3784	^3^LE + CT
**1·N** ^**o**^	425	615	612	1.2	4.3	–^c^	–^c^	–^c^	^3^CT
**1·A** ^**o**^	366	620	585	2.6	7.8	21	0.8076	3.0385	^3^CT
**1·E**	380	634	585	1.2	17.4	6	0.5000	7.8333	^3^CT
**1·O** ^d^	450	650	650	0.5	2.5	9	1.6544	16.7275	^3^CT

a The average amplitude-weighted emission lifetime for multiexponential decays (τ_av_ = Σ*A*_*i*_τ_*i*_, where *A*_*i*_—the weight of the exponent) measured at λ_em_ maxima in MCS (TCSPC for **a1**) mode.

b *k*_r_ = Q.Y./τ_av_, *k*_nr_ = (1 – Q.Y.)/τ_av_.

c Cannot be accurately measured due to the limited trace quantity of the sample upon crystallization.

d Amorphous phase obtained from **1·N/1·A** upon drying at 100 °C *in vacuo* for 30 min. n.d.—Not determined due to low emission intensity. The solid-state photophysical measurements were performed on the phase-pure crystalline/microcrystalline forms of **1·C**, **1·B**, **1·N**, **1·W**, **1·E**, **1·A** ^**o**^, and **1·M**, with single-crystal measurements performed where crystals of sufficient quality and quantity were available (see Supporting Information for more details). Because these solvatomorphs are intrinsically susceptible to gradual solvent loss, the measurements were conducted under solvent-retaining inert conditions to preserve the corresponding crystalline phases and minimize contributions from desolvated or amorphous material.

In the absence of anion−π^+^ interactions, the noncovalently bound chloroform molecules do not act merely as spectators but may influence the excited-state dynamics through an external heavy-atom effect, enhancing SOC and facilitating triplet-state population and radiative decay while retaining the dominant ^3^LE character of the emissive state. Thus, prolonged drying of **1·C** at 320 K yields a solvent-free amorphous phase (Figure S29) that is nearly non-emissive, likely due to a substantial decrease in the radiative decay probability of the ^3^LE state. The loss of proximal CHCl_3_ is expected to diminish the chlorine-assisted heavy-atom contribution to SOC, thereby suppressing efficient triplet-state population and radiative decay.

The substitution of chloroform with bromoform affords phase **1·B** (Figure S32), which has the same overall solvent and anion arrangement as **1·C** and exhibits similar vibronically structured emission assigned mainly to the ^3^LE state (λ_em_ = 500, 529, 568 nm; Figure 5A and Table 1), in agreement with TDDFT calculations (Figure S33). The replacement of chlorine by the heavier bromine atoms is accompanied by a nearly threefold increase in the radiative rate (*k*_r_ = 960 s^–1^), consistent with enhanced heavy-atom-assisted SOC and a higher radiative transition probability of the triplet state. The above comparison further highlights the role of the local heavy-atom environment in modulating triplet-state population and relaxation.

The emergence of symmetrical anion−π^+^ interactions in **1·A** and **1·N**, resulting from structural rearrangement relative to **1·C**, shifts the emission color from green to greenish-yellow (Figure 5A and Supporting Information Videos S1, S2, S3 and S4), corresponding to a bathochromic shift of approximately 1180–1550 cm^–1^. At 297 K, the emission bands of **1·A** and **1·N** are broader (FWHM: 3335 cm^–1^ for **1·C** vs 3395/3335 cm^–1^ for **1·A**/**1·N**) yet retain partial vibronic structure that becomes more pronounced at 77 K (Figures S34, S35, Tables S7 and S8). Subtle differences in emission maxima, which are slightly red-shifted for **1·N** (λ_em_ = 521, 551, 584 nm) relative to **1·A** (λ_em_ = 511, 544, 577 nm), might reflect the discrepancy in anion hapticity (η^4^- in **1·A** vs η^2^- in **1·N**) and the sensitivity of the excited-state energy to the mode of anion−π^+^ arrangement and solvated molecules. Both **1·A** and **1·N** exhibit radiative rate constants nearly two orders of magnitude higher (*k*_r_ = 0.6–1.0 × 10^4^ s^–1^; τ_av_ = 23.0 and 22.8 μs, respectively) than that of **1·C**, evidencing enhanced SOC associated with short anion−π^+^ contacts, i.e., the heavy atom effect, established upon ion pair reconfiguration. In contrast to the nearly pure ^3^LE character of **1·C**/**1·B**, the lowest triplet states of **1·A** and **1·N** have a substantial contribution of iodide → π* charge-transfer (CT, Figure 5B). Theoretical calculations reveal that the charge-transfer (CT) character exceeds 70% in **1·A** and **1·N**, irrespective of the in-plane or out-of-plane orientation of the iodide counterions (Figures S36–S40). To further probe the coexistence of LE and CT contributions, area-normalized time-resolved emission measurements were performed for representative **1·C** and **1·N** at 297 K (Figures S41 and S42). No appreciable time-dependent change in spectral shape or emission maximum was observed. Moreover, excitation–emission maps revealed a single well-defined emission profile without evidence for a second spectrally distinguishable emissive species (Figure S41). The observed phosphorescence is therefore consistent with possible sequential population of kinetically coupled ^3^LE/^3^CT manifolds whose substantially overlapping emission bands preclude their independent resolution by spectral evolution alone. In agreement with prior reports and our theoretical findings, the broad, structureless excitation bands observed at 360–410 nm are attributed to the formation of a ground-state iodide−π CT complex.^29,30,35^ The low-intensity structured absorption bands at longer wavelengths (450–470 nm) upon cooling can be assigned to a forbidden S_0_ → T_1_ (π → π*) transition, which are only slightly red-shifted relative to that of naphthalene,^36^ confirming the ^3^LE nature of the lowest-lying triplet manifold.

To further substantiate the role of SOC in the enhanced phosphorescence, we calculated the radiative lifetimes of the T_1_ states for **1·C**, **1·N**, and **1·A** (Table S9). The calculated values reproduce the experimental trend, with substantially faster radiative decay predicted for **1·A** and **1·N** than for **1·C**. Analysis of the individual SOC-mediated intensity-borrowing pathways shows that coupling of T_1_ with low-lying singlet states, ⟨S_*n*_|H_SO_|T_1_⟩, provides the dominant contribution, whereas the corresponding T_*n*_–S_0_ coupling, ⟨T_*n*_|H_SO_|S_0_⟩, plays a comparatively minor role. In particular, the S_1_–S_3_ states constitute the main intensity-borrowing channels, with substantially larger admixture coefficients (δ_*n*_) for **1·A**/**1·N** than for **1·C**. These results provide theoretical support for enhanced SOC-mediated intensity borrowing in the reconfigured ion pairs and rationalize their substantially higher radiative rates.^34^ However, it is worth noting that, despite the increase in the *k*_r_ values for **1·A** and **1·N**, the greater contribution of CT character also markedly promotes nonradiative decay pathways by a factor of approximately 200. As a result, the phosphorescence quantum yields decrease to 13% and 23% for crystalline **1·A** and **1·N**, respectively.

Likewise, the presence of two heavy atoms in proximity to the dication in the I^–^–C_6_H_4(napht)_–I^–^ configuration has a strong impact on the excited-state properties of contact ion pairs **1·N** ^**o**^, **1·A** ^**o**^, and **1·E** (Figures 5, 6, S34, S35, Tables 1, S7 and S8). This is evidenced by (i) the steady-state broad and structureless photoemission profiles in the lower-energy orange region (λ_em_ = 615/620/634 nm for **1·N** ^**o**^/**1·A** ^**o**^/**1·E**), and (ii) the shortest phosphorescence lifetimes observed within this family (τ_av_ = 1.2/2.6/1.2 μs for **1·N** ^**o**^/**1·A** ^**o**^/**1·E**). This behavior is consistent with that of the related isomeric 1,4-(diphosphonium)­naphthalene diiodide salt showing ^3^CT­(anion−π^+^) phosphorescence^29,35^ and is also further confirmed by our TD-DFT calculations for **1·A** ^**o**^ (Figures 6C and S43), which indicate that the T_1_ state possesses an approximately 90% ^3^CT character. Hence, the nearly pure ^3^CT emission in **1·N** ^**o**^/**1·A** ^**o**^/**1·E** is finely tuned by the solvent, which is consequently modulated by the strength of anion−π^+^ interactions and thereby controls the energy of the excited state.

**Figure 6:**
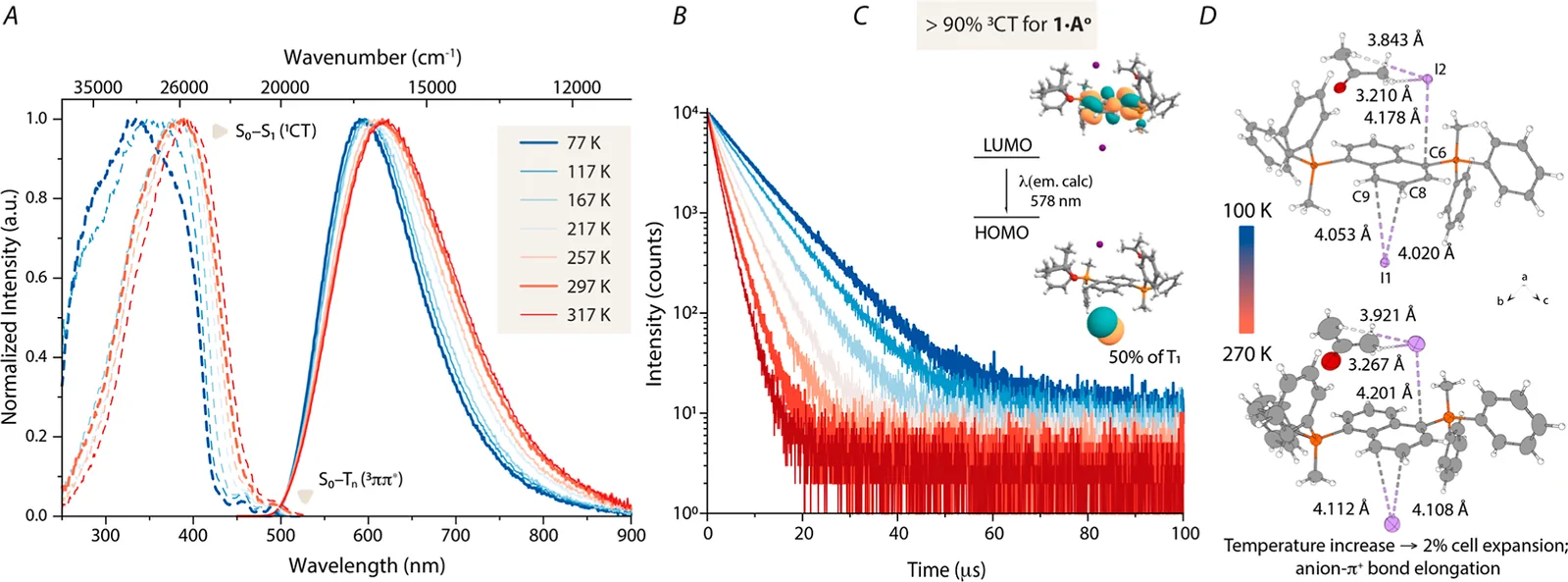
(A) Variable temperature (VT) excitation (dashed) and emission (solid), and (B) time-resolved decays of **1·A** ^**o**^; (C) selected molecular orbitals that mainly compose T_1_ state of **1·A** ^**o**^; (D) SCXRD crystal structures of **1·A** ^**o**^ with depicted shortest interactions at 100 and 270 K showing anion–π bonds contraction; displacement ellipsoids are shown at the 50% probability level.

The phase **1·O** (Figures 3 and S29), formed upon thermal treatment of **1·N**/**1·A**, exhibits photophysical characteristics consistent with the phases exhibiting substantial CT character (**1·N** ^**o**^/**1·A** ^**o**^/**1·E**). Its red-shifted structureless emission (λ_em_ = 650 nm), low-energy excitation (λ_exc_ = 450 nm), short lifetime (0.5 μs), and fast *k*_r_ (1.65 × 10^5^ s^–1^) indicate substantial CT character (Figures 5C, S34 and S35). Although the semi-amorphous nature of **1·O** precludes a definitive structural assignment, these properties suggest that thermal reconfiguration of **1·N**/**1·A** generates a species with an active anion–π^+^ CT emissive state.

The observed excited-state lifetime of crystalline **1·A** ^**o**^ is shorter by approximately a factor of nine relative to **1·A**, indicating further enhancement of SOC. As a consequence, the T_1_ → S_0_ phosphorescence occurs at a higher rate with *k*_r_ of ca. 0.81 × 10^5^ s^–1^. Similar to previously reported pyridinium and phosphonium ionic pairs, it is plausible to expect orbital compositions of S_1–3_ and T_1–3_ states to be hybrid in nature, derived from *n* → π* and π → π* configurations.^29,30,35^ Such mixing facilitates efficient SOC and accelerates ISC in accordance with El-Sayed’s rules. Importantly, conservation of orbital angular momentum required for SOC is enabled by the reorientation of the iodide p orbital participating in the electronic transition, for example, from p_*z*_ in S_1_ to p_*x*_/p_*y*_ in T_*n*_. In addition, orbital analysis of **1·A**, including the co-crystallized acetone molecules (Figure S40), reveals minor contributions from solvent lone-pair orbitals to the S_1–3_ and T_1–3_ states, suggesting that solvent-assisted *n* → π* character plays a secondary role relative to the dominant heavy-atom contribution of the iodide ions.

The variable-temperature (VT) studies of **1·A** ^**o**^ (Figure 6A,B) reveal a moderate hypsochromic shift of the emission maximum upon cooling from 620 to 585 nm (Δ*ν* ≈ 970 cm^–1^), accompanied by a slight narrowing of the emission band (ΔFWHM ≈ 643 cm^–1^). Such a change can be interpreted as a rigidochromic response of the emissive state to the suppression of lattice dynamics at low temperature. Consistent with this interpretation, variable-temperature single-crystal X-ray diffraction (VT-SCXRD) analysis (Figures 6D and S44) reveals a unit-cell contraction of approximately 2.7% upon cooling from 270 to 100 K. Concurrently, the anion−π^+^ interactions become stronger, as reflected by the shortening of the anion−π^+^ contacts, which decrease from η^1^–4.20/η^2^–4.11/4.12 Å at 270 K to η^1^–4.18/η^2^–4.05/4.02 Å at 100 K. Thus, iodide anion relocation toward the electron-deficient π^+^ surface shortens the anion−π^+^ contact and thereby strengthens through-space electronic and electrostatic coupling. The closer I^–^···π^+^ interaction increases mixing between occupied iodide-centered orbitals and low-lying orbitals of the aromatic cation, resulting in a greater CT contribution to the emissive excited state. The closer proximity of iodide may additionally enhance the external heavy-atom contribution to spin–orbit coupling, while the stronger electronic coupling and greater structural constraint within the ion pair may suppress structural fluctuations that contribute to nonradiative relaxation. Thus, anion relocation provides a structural means of simultaneously modulating the excited-state energy, CT character, and relaxation dynamics. Importantly, absolute quantum yield measurements reveal an increase from 21% at 297 K to 71% at 77 K (Table S10). Analysis of the corresponding experimental decay rate constants shows that cooling results in only a modest increase in the radiative decay rate constant (*k*_r_ = 8.1 × 10^4^ to 9.1 × 10^4^ s^–1^), whereas the nonradiative decay rate decreases by approximately eightfold (*k*_nr_ = 3.0 × 10^5^ to 3.7 × 10^4^ s^–1^). These observations indicate that the enhanced emission efficiency at low temperature arises predominantly from the suppression of nonradiative relaxation. The modest increase in *k*_r_ may additionally be associated with the cooling-induced shortening of the anion−π^+^ contact, which strengthens electronic coupling and increases the CT contribution to the emissive state. Cooling also leads to a hypsochromic shift of the ^1^CT absorption bands in the excitation spectra (λ_exc_ = 370–400 nm), resulting in improved resolution of low-energy vibronically structured features in the 450–500 nm region, attributed to direct population of the triplet ^3^(π → π*) state.

In contrast to the intense phosphorescence observed for **1·C**, **1·B**, **1·N**, and **1·A**, the highly solvated phases **1·W** and **1·M** exhibit weak fluorescence at 297 K (λ_em_ = 415, 436 nm). This behavior is attributed to extensive solvation of the iodide anions, which inhibits the formation of close cation–anion contacts (Tables 1, S7, S8, Figures S45–S47, Videos S5 and S6). Upon cooling, both phases display vibronically resolved, long-lived ^3^LE emission characteristic of the naphthalene chromophore, closely resembling the low-temperature behavior of **1·C/1·B** (Tables 1, S7 and S8). These results suggest that the heavy-atom effect remains operative in **1·W** and **1·M**; however, at room temperature, it is insufficient to compete effectively with rapid nonradiative deactivation pathways. This interpretation is consistent with the spatial separation of the iodide counterions from the π^+^ surface imposed by solvent coordination.

The optical readouts arising from selective phase transitions **1·W/1·M** → **1·C** provide the basis for sensing systems. To prove the selectivity of this response toward chloroform, the behavior of **1·W/1·M** was examined upon exposure to other chlorinated solvents. Treatment with dichloromethane, carbon tetrachloride, or 1,2-dichloroethane produced no emission enhancement, and the samples remained essentially non-emissive. Only exposure of **1·C** to dichloromethane generated transient emissive states (1·D*), and this metastable phase was found to be prone to a rapid conversion within 5–10 min into non-emissive form **1·D** in the presence of excess solvent. Its luminescence signatures (Tables S7, S8 and Figure S48) suggest that 1·D* adopts an anion−π^+^ arrangement similar to that found in **1·N/1·A**, whereas a non-emissive stable solvatomorph **1·D** reveals no significant iodide−π^+^ contacts, as was confirmed by SCXRD (Figure S25).

Thus, among the examined chlorinated solvents, only chloroform triggers a structural transformation that results in bright ^3^LE phosphorescence, underscoring the high specificity of solvent–cation interactions and highlighting the potential of such dynamic ion pairs as selective solid-state sensing platforms.

The concept demonstrated herein is expected to inspire the development of a wider family of electron-deficient polyaromatic ionic materials. In our ongoing studies we are exploring the extension of this molecular design strategy by replacing the naphthalene core with larger π-conjugated electron-deficient frameworks while preserving the underlying structural principles of solvent-promoted anion translocation over long ranges beyond 4.3 Å demonstrated for **1**. We anticipate that such structurally analogous systems will enable systematic tuning of their structural and electronic properties and provide versatile platforms for molecular recognition, guest-responsive assemblies, and photocatalysis.

## Conclusions

In summary, this work presents the first example of solvent-fueled solid-state anion relocation within an ion-paired crystal, whereby solvent exchange and uptake induce reversible rearrangement of the crystalline lattice, accompanied by pronounced changes in photophysical properties. The naphthalene-derived diphosphonium–diiodide salt serves as a versatile platform in which solvent exchange/uptake triggers multiple phase transitions associated with the migration of the anion around the polyaromatic motif over distances of approximately 2.2–4.3 Å. Such guest solvent-dependent motion reversibly modulates the anion−π^+^ interactions and affects the nature of the excited state and its relaxation, allowing the controlled switching among deep-blue fluorescence, green-to-yellow locally excited phosphorescence, and orange-red charge-transfer phosphorescence. Combined structural and theoretical investigations reveal that the size and hydrogen-bonding capability of the guest solvent molecules govern a delicate balance among ion solvation, hydrogen bonding, and anion−π^+^ interactions, thereby directing both the phase behavior and emission characteristics. Importantly, the observed ion migration is not merely a structural response but actively dictates the nature of the emissive excited state, providing a direct link between solid-state ionic motion and optical function.

Altogether, these findings establish dynamic ion relocation as a previously unexplored design principle for functional crystalline materials. More broadly, they demonstrate how directional ionic motion in the solid state can be translated into programmable luminescent outputs, offering new opportunities for the development of adaptive photonic materials, responsive sensors, information-storage systems, and stimuli-responsive optoelectronic devices.

## Supplementary Material















## Notes

Experimental and computational details, X-ray structure analyses, copies of NMR and HPLC data, photoluminescence data, and computational details (PDF)
Video S1 of phase transition (**1·C** → **1·N**) upon solvent fueling (MOV)
Video S2 of phase transition (**1·N** → **1·C**) upon solvent fueling (MOV)
Video S3 of phase transition (**1·C** → **1·A**) upon solvent fueling (MOV)
Video S4 of phase transition (**1·A** → **1·C**) upon solvent fueling (MOV)
Video S5 of phase transition (**1·M** → **1·C**) upon solvent fueling (MOV)
Video S6 of phase transition (**1·C** → **1·M**) upon solvent fueling (MOV)
