# Supplementary material for: Solvent-Fueled Anion Translocation in Crystalline Phosphonium Salt
Source: J Am Chem Soc. 2026 Sep 16;148(38):41151–60. doi: 10.1021/jacs.6c12003 (PMC13632557; doi:10.1021/jacs.6c12003)
Supplement: Supplementary file 1 [file ja6c12003_si_001.pdf]

Supporting information

Solvent-Fueled Anion Translocation in Crystalline Phosphonium Salt

Eetu Hakkarainen,[a] Pei-Ying Huang,[b] Chen-Yu Lin,[b] Pi-Tai Chou,[b]* Igor O. Koshevoy,[a]* Glib V. Baryshnikov,[c]* Andrey Belyaev[a]*

[a] Department of Chemistry and Sustainable Technology, University of Eastern Finland, Yliopistokatu 7, Joensuu, 80101, Finland

[b] Department of Chemistry, National Taiwan University, Taipei, 10617 Taiwan, Republic of China

[c] Laboratory of Organic Electronics, Department of Science and Technology, Linköping University, Norrköping, 60174, Sweden

E-mail: andrei.beliaev@uef.fi; igor.koshevoy@uef.fi; glib.baryshnikov@liu.se; chop@ntu.edu.tw

Table of Contents

**Experimental Procedures / Characterization methods**	**3**
**Figures S1-10.** Copies of ^{1}H, ^{31}P{^{1}H}, and ^{13}C{^{1}H} NMR spectra.	**8**
**Figure S11.** HPLC chromatographs of **1**.	**15**
**Tables S1-3.** Crystal data and structure refinement.	**16**
**Figures S12-S13.** Packing representations of **1·C**.	**19**
**Figure S14.** Discrete ion pairs **1·C**, **1·A**, **1·N**, **1·W**, **1·A^{o}** and **1·N^{o}** and their packing fragments.	**20**
**Figure S15.** Cyclic PXRD data of the phase transitions **1·C**↔**1·A** and **1·C**↔**1·N**.	**21**
**Figures S16-S17**. Packing representations of **1·A** and **1·N**.	**22**
**Figure S18.** Overlays of the fragments of **1·C/1·A^{o}**, **1·C/1·N^{o}**, **1·C/1·A**, **1·C/1·W**, and **1·C/1·M**.	**24**
**Figure S19.** Fragments of the cations **1·C**, **1·B**, **1·N**, **1·A**, **1·N^{o}**, **1·A^{o}**, and **1·E**.	**25**
**Figure S20.** Crystal structure of **1·A^{g}**.	**25**
**Table S4.** Bond lengths, energies, and topological characteristics of the electron density distribution at the (3; -1) critical points of the I---X bonds (X=C,H) of **1·A** and **1·A^{o}** crystal structures.	**26**
**Figure S21.** QTAIM analysis of **1·A** and **1·A^{o}**.	**26**
**Table S5.** Bond lengths, energies, and topological characteristics of the electron density distribution at the (3; -1) critical points of the I---X bonds (X=C, H) of **1·N** and **1·N^{o}** crystal structures.	**27**
**Figure S22.** QTAIM analysis of **1·N** and **1·N^{o}**.	**27**
**Figure S23.** Crystal structure of **1·E**.	**27**
**Table S6.** Bond lengths, energies, and topological characteristics of the electron density distribution at the (3; -1) critical points of the I---X bonds (X=C, H, Cl) of **1·C** and **1·E** crystal structures.	**28**
**Figure S24.** QTAIM analysis of **1·C** and **1·E**.	**28**
**Figure S25.** Crystal structures of **1·M** and **1·D**.	**29**
**Figure S26-S28.** TGA and DSC curves of the solvated forms of **1**.	**30**
**Figure S29–S30.** Simulated and experimental PXRD patterns for **1·X**.	**33**
**Figure S31.** Molecular orbitals corresponding to the T_1 excited state of the **1·C** crystal structure fragment calculated by the wB97XD/DGDZVP method.	**35**
**Figure S32.** Crystal structure of **1·B** with in-plane I–cation arrangement and solvation of cationic core with molecules of bromoform.	**35**
**Figure S33.** Molecular orbitals corresponding to the T_1 excited state of **1·B** crystal structure fragment calculated by wB97XD/DGDZVP method.	**36**
**Figure S34.** Excitation and emission spectra of **1·C**, **1·B**, **1·N**, **1·A**, **1·N^{o}**, **1·A^{o}**, **1·E** at 77 K.	**37**
**Figure S35.** Decay lifetimes of the excited state and their fits for **1·N**, **1·A**, **1·C**, **1·B**, **1·N^{o}**, **1·A^{o}**, and **1·E** at 297 and 77 K.	**38**
**Tables S7-8.** Summaries of the photophysical properties of **1** in the solid state at 297/77 K.	**39**
**Figures S36-40.** Molecular orbitals corresponding to the T_1 excited states of **1·N**, **1·A** crystal structure fragments with out-of-plane or in-plane orientations of iodide counterions.	**41**
**Figures S41-S42.** Excitation-emission mapping and time-resolved emission spectra of the **1·C** and **1·N.**	**43**
**Table S9.** Experimental and calculated T_1-state radiative lifetimes, singlet- and triplet-state spin–orbit coupling matrix elements, and admixture coefficients (δ_n) for **1·C**, **1·A**, and **1·N**.	**45**
**Figure S43.** Molecular orbitals corresponding to the T_1 excited states of **1·A^{o}**.	**45**
**Figure S44.** Variable temperature SCXRD measurements of **1·A^{o}**.	**46**
**Table S10.** Summary of the variable temperature time-resolved measurements for **1·A^{o}**.	**46**
**Figure S45.** Excitation and emission profiles of **1·W** and **a1** at 297 and 77 K.	**47**
**Figure S46.** Time-resolved data for **1·W** and **a1** at 297 and 77 K.	**48**
**Figure S47.** Excitation and emission profiles, time-resolved data of **1·M**.	**49**
**Figure S48.** Excitation and emission profiles of **1·D*** at 297 and 77 K; Time-resolved emission decays and their fits for solid **1·D*** at 297 and 77 K.	**49**
**References**	**50**

Experimental Procedures

General Comments: The synthesis of 1,5-bis(diphenylphosphino)naphthalene was adapted from a previously published method.[1] Tetrahydrofuran (THF), diethyl ether (Et_2O), and toluene were distilled over Na-benzophenone ketyl under a nitrogen atmosphere before use. Other reagents and solvents were used as received. The reaction was carried out under an inert nitrogen atmosphere. The solution ^{1}H, $^{13}C\{^{1}H\}$, and $^{31}P\{^{1}H\}$ NMR spectra were recorded on a JEOL JNM-ECZ500 500 MHz spectrometer. Microanalyses were carried out in the analytical laboratory of the University of Eastern Finland.

Single-Crystal X-ray Structure Determinations (SCXRD): The single crystals were immersed in a film of NVH mounted on a polyimide microloop (MiTeGen), transferred to a stream of cold nitrogen (Cryostream 700 cooling system by Oxford Cryosystems), and measured at a temperature of 100/150 K or at variable temperatures (100–320 K). The X-ray diffraction data were collected on a Rigaku XtaLAB Synergy R (Cu) or SuperNova (Mo) diffractometers with a HyPix-Arc 100 detector using mirror-monochromated Cu Kα (λ = 1.54184 Å) or MoKα (λ = 0.71073 Å) radiation (INCOATEC microfocus sealed tube). The frames were integrated with the CrysAlisPro software package using a narrow-frame algorithm. The CrysAlisPro program package was used for cell refinements and data reductions. The structure was solved using the intrinsic phasing method (SHELX)[2,3] refined with OLEX2-1.5, and visualized with the Mercury 2024.3.1 and Diamond 4.0 programs. A semiempirical absorption correction (SADABS) was applied to all data.

In **1·N°**, the acetonitrile co-solvent molecule was modeled as disordered over two positions with refined occupancies of 0.59/0.41, constrained to sum to unity. Corresponding atoms of the two disorder components (N1/C37/C38 and N1A/C37A/C38A) were restrained to have similar geometries using distance similarity restraints. Selected bond length distances were restrained to chemically reasonable values. Anisotropic displacement parameters of the disordered atoms were restrained using SIMU and RIGU restraints to maintain physically reasonable displacement ellipsoids. The minor disorder component was additionally restrained to have displacement parameters comparable to the equivalent isotropic values because of its lower occupancy. The occupancies of all atoms belonging to the two disorder components, including their hydrogen atoms, were linked through a free variable such that the total occupancy was unity.

In **1·M**, the crystal structure contains four methanol solvent molecules. One methanol molecule was modeled as disordered over two positions 0.42/0.58 with refined occupancies constrained to sum to unity using a free variable. The occupancies of the corresponding oxygen, carbon, and hydrogen atoms were linked during refinement. The occupancies of the two methanol molecules could not be refined reliably because of weak and diffuse electron density associated with the highly disordered solvent sites. Their occupancies were therefore fixed at 0.50 and 0.333, respectively, to obtain a stable and chemically reasonable refinement. Selected anisotropic displacement parameters of the solvent atoms were restrained to maintain physically meaningful displacement ellipsoids.

In **1·C** and **1·B**, the crystal structures contain independent chloroform (1 molecule within 0.43/0.57 occupancies)/bromoform (2 molecules with 0.29/0.71 and 0.43/0.57 occupancies) solvent molecules exhibiting positional disorder. Solvent molecules were modeled over two alternative orientations with refined occupancies constrained to sum to unity using separate free variables. The occupancies of the corresponding bromine, chlorine carbon, and hydrogen atoms within each disorder model were linked during refinement. Similarity restraints were applied to corresponding C–Cl/Br bond lengths and selected Cl···Cl/Br···Br distances of the disorder components to maintain chemically reasonable geometries. Equivalent carbon atoms of one disordered bromoform molecule in **1·B** were restrained to have identical anisotropic displacement parameters. These restraints resulted in stable refinement and physically reasonable displacement ellipsoids for the disordered solvent molecules.

All non-hydrogen atoms were refined anisotropically. Hydrogen atoms were included in the structure factor calculations. All hydrogen atoms were assigned to idealized geometric positions. The omitted reflections were limited to statistical outliers (bad HKL reflections) identified during refinement, where the disagreement between observed and calculated intensities ($|F^2_o - F^2_c|$/esd) exceeded ten times the estimated standard deviation. The crystallographic details are summarized in Tables S1–3. CCDC entries **2542718-2542730** and **2542732** contain supplementary crystallographic data for this paper.

Powder X-ray Diffraction Analysis (PXRD): Data were collected from bulk polycrystalline material (three averaged replicas from ground crystalline material) using a Rigaku XtaLAB Synergy-S with Cu K_α radiation (λ = 1.54184 Å, Microfocus beam, Divergence 2.3, 600 sec exposure time) with Gandolfi strategy for data collection. Calculated data were generated using Mercury 2024.3.1 (Build 428092) software.

Thermogravimetric Analysis (TGA) and Differential Scanning Calorimetry (DSC): TGA measurements were carried out using a Mettler Toledo TGA 2 instrument in the temperature range of 30–600 °C with a heating rate of 10 °C min^{-1}, under a nitrogen atmosphere (flow rate 50 ml min^{-1}). 70 µl aluminum oxide pans were used for the experiments. Prior to the experiments, the pans were tempered at 1000 °C for 10 minutes and kept in an exicator to remove and protect from humidity. DSC measurements were carried out using a Mettler Toledo DSC 823^e instrument in the temperature range of 25–500 °C with a heating rate of 10 °C min^{-1}, under a nitrogen atmosphere (flow rate 50 ml min^{-1}). 40 µl aluminum pans were used for the experiments, with an empty pan serving as a reference.

High-Performance Liquid Chromatography (HPLC): The HPLC chromatograms were obtained from an Agilent 1260 Infinity II liquid chromatograph equipped with a diode array detector (G71115A, monitored at 260, 290 and 320 nm), binary pump, heated column compartment at 25 °C, and autosampler controller. Phenomenex Gemini 3µm C18 column (3.5-micrometer diameter particle size, 4.6×150 mm) was used for all analytical experiments. 2 µl aliquot (5 mM in 2:1 H_2O:DMSO) was injected and eluted with H_2O/NCMe solution (TFA 0.1%) in a gradient mode (5-30% of NCMe) within 16 minutes.

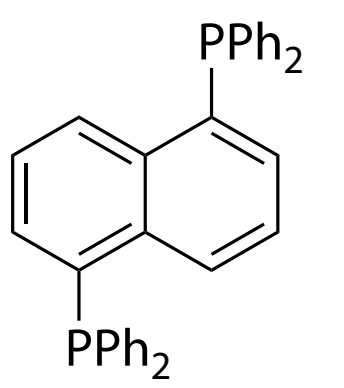

1,5-Bis(diphenylphosphino)naphthalene (a1). A 1.6 M solution of n-BuLi in hexanes (8.7 ml, 14 mmol, 2 equiv.) was added dropwise to a solution of 1,5-dibromonaphthalene (2.0 g, 7 mmol, 1 equiv.) in THF (40 ml) at -70 °C, and the resulting mixture was stirred at this temperature for 1.5 hours. Chlorodiphenylphosphane (3.1 g, 14 mmol, 2 equiv.) was added dropwise at -70 °C, and the mixture was allowed to reach room temperature. Stirring was continued for 16–24 hours, after which a white precipitate was collected and washed with cold THF (2×15 ml). The resulting solid was dried *in vacuo* to give a white crystalline powder (1.9 g, 55 %). Spectroscopic data matched previously reported data.[4]

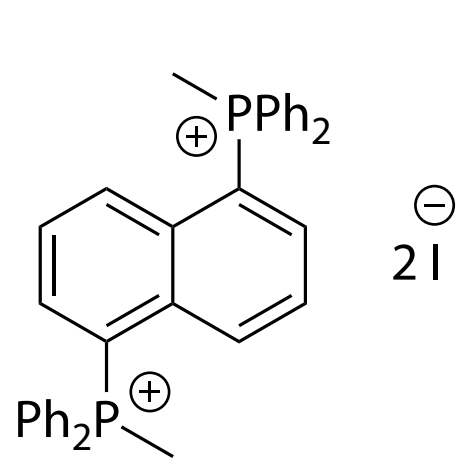

1,5-Naphthalene-diylbis(methyldiphenylphosphonium) diiodide (1).

Methyl iodide (193 mg, 1.35 mmol, 5 equiv.) in 2 ml of $CHCl_3$ was added dropwise to a degassed solution of 1,5-bis(diphenylphosphino)naphthalene (135 mg, 0.27 mmol, 1 equiv.) dissolved in $CHCl_3$ (15 ml). The reaction mixture was stirred at room temperature for 16 hours, and then cooled to 0 °C. The resulting precipitate was centrifuged and washed with Et_2O (3×10 ml). The product was dried in vacuo at 80 °C for 2 hours and collected, giving **1** as a pale-yellow powder (188 mg, 89%). ^{1}H NMR (acetonitrile-*d*$_3$, 297 K, 500 Hz) δ: 8.18 (d, 8.6 Hz, $–C_{10}H_6–$, 2H), 7.90 (t, 7.4 Hz, *p*-PPh_2, 4H), 7.76 (m, $–C_{10}H_6–$, *o*+*m*-PPh_2, 18H), 7.66 (t, 8.0 Hz, $–C_{10}H_6–$, 2H), 3.05 (d, 13.4 Hz, CH_3, 6H). ^{1}H NMR (DMSO-*d*$_6$ 297 K, 500 Hz) δ: 8.29 (d, 8.5 Hz, $–C_{10}H_6–$, 2H), 7.89 (m, $–C_{10}H_6–$, PPh_2, 12H), 7.80 (m, $–C_{10}H_6–$, PPh_2, 10H), 7.70 (dd, 16.8 Hz, 7.4 Hz, $–C_{10}H_6–$, 2H), 3.37 ppm (overlap with H_2O signal, CH_3, 6H). $^{13}C\{^1H\}$ NMR (DMSO-*d*$_6$, 297 K, 127 Hz) δ: 138.6 (d), 135.1 (s), 133.4 (d), 132.6 (m), 130.4 (d), 128.3 (d), 119.5 (d), 118.5 (d), 79.2 (s), 8.8 (d). $^{31}P\{^1H\}$ NMR (DMSO-*d*$_6$, 297 K,

202 Hz) δ: 23.9 ppm (s). Anal. Calc. for $C_{36}H_{32}P_2I_2$: C, 55.41; H, 4.13. Found: C, 55.10; H, 4.28. ESI-MS (*m/z*): found for $[M]^{2+}$ 263.0991 (calculated 263.0984).

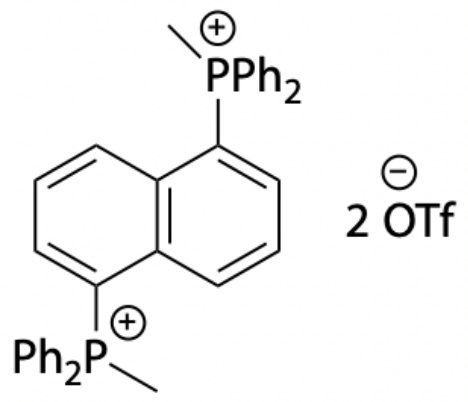

1,5-Naphthalene-diylbis(methyldiphenylphosphonium) trifluoro-methanesulfonate (1^{OTf}). Methyl triflate (0.07 mL, 0.60 mmol, 2.0 equiv) was added to a degassed solution of 1,5-bis(diphenylphosphino)naphthalene (150 mg, 0.30 mmol, 1.0 equiv) in $CHCl_3$ (15 mL). The reaction mixture was stirred for 30 min at room temperature, and the resulting precipitate was collected by filtration, washed with Et_2O (3 × 20 mL), and dried *in vacuo* to afford $\mathbf{1^{OTf}}$ as a white powder (144 mg, 58%). ^{1}H NMR (CD_3CN, 297 K, 500 MHz) δ: 8.13 (d, 8.6 Hz, $-C_{10}H_6-$, 2H), 7.88 (t, 7.3 Hz, *p*-PPh$_2$, 4H), 7.73 (m, $-C_{10}H_6-$, *o+m*-PPh$_2$, 18H), 7.63 (m, $-C_{10}H_6-$, 2H), 2.97 (dd, 13.6 Hz, 3.9 Hz, CH_3, 6H). $^{31}P\{^1H\}$ NMR (CD_3CN, 297 K, 202 MHz) δ: 22.9 ppm (s).

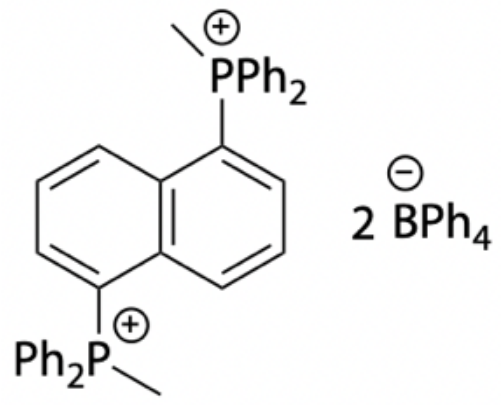

1,5-Naphthalene-diylbis(methyldiphenylphosphonium) tetraphenyl borate (1^{BPh4}). Synthesized from **1** (30 mg, 0.04 mmol, 1.0 equiv) and $AgBPh_4$ (34 mg, 0.08 mmol, 2.05 equiv) in acetonitrile (5 mL). The reaction mixture was stirred at room temperature, and the resulting AgI precipitate was removed by filtration and washed with acetonitrile (2 mL). The combined filtrate and washings were concentrated to dryness under reduced pressure. The resulting beige solid was redissolved in acetonitrile (5 mL), followed by precipitation with diethyl ether to afford $\mathbf{1^{BPh_4}}$ as a beige solid (44 mg. 50%).^{1}H NMR (CD_3CN, 297 K, 500 MHz) δ: 8.13 (d, 8.5 Hz, $-C_{10}H_6-$, 2H), 7.86 (s, PPh$_2$, 4H), 7.66 (m, $-C_{10}H_6-$, PPh$_2$, BAr^F_4, 44H), 2.95 (dd, 13.6 Hz, 3.8 Hz, CH_3, 6H). $^{31}P\{^1H\}$ NMR (CD_3CN, 297 K, 202 MHz) δ: 22.8 ppm (s).

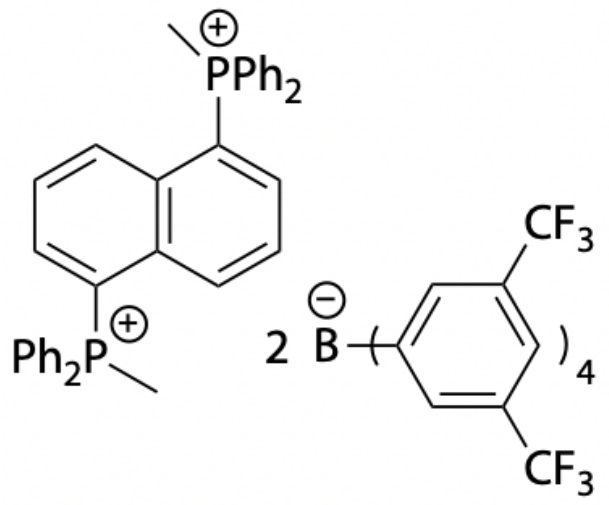

1,5-Naphthalene-diylbis(methyldiphenylphosphonium) tetrakis(3,5-bis(trifluoromethyl)phenyl) borate (1^{BARF}). ·BArF was synthesized by mixing **1** (23 mg, 0.03 mmol, 1.0 equiv) with NaBArF (52 mg, 0.06 mmol, 2.0 equiv) in DCM (5 mL). The resulting suspension was stirred for 45 min at room temperature, after which the precipitate was removed by filtration. The filtrate was concentrated to dryness under reduced pressure, and the resulting solid was extracted with Et_2O (3 × 15 mL). The combined extracts were concentrated to afford $\mathbf{1^{BARF}}$ as a white solid (24 mg, 36%). ^{1}H NMR (DMSO-*d$_6$* 297 K, 500 MHz) δ: 8.23 (d, 8.5 Hz, $-C_{10}H_6-$, 2H), 7.87 (t, 7.7 Hz, *p*- PPh$_2$, 4H), 7.81 (m, $-C_{10}H_6-$, PPh$_2$, 8H), 7.74 (m, $-C_{10}H_6-$, PPh$_2$, 10H), 7.64 (m, $-C_{10}H_6-$, 2H) 7.13 (s, BPh$_4$, 16H), 6.88 (q, 6.3 Hz, BPh$_4$, 16H), 6.74 (q, 6.4 Hz, *p*-BPh$_4$, 8H), 3.28 ppm (overlap with H_2O signal, CH_3, 6H). $^{31}P\{^1H\}$ NMR (DMSO-*d$_6$*, 297 K, 202 MHz) δ: 24.0 ppm (s).

Solubility: Solubility measurements were performed at room temperature using 10 mg of the compound suspended in the respective pure solvent. For acetonitrile, 20 mg of the compound was used because of its comparatively higher solubility. The suspensions were allowed to equilibrate for 12 h at room temperature under constant conditions without access to air. After equilibration, the undissolved material was separated by centrifugation, and the recovered solid residue was isolated and weighed. The amount dissolved was calculated from the difference between the initial sample mass and the mass of the recovered undissolved residue, and the solubility was expressed accordingly.

Solvent	$CHCl_3$	DCM	MeOH	H_2O	Acetone	MeCN
Solubility (mg/ml)	0.3	1.8	8.7	6.2	3.0	11.7

Crystallization details: Single crystals suitable for X-ray analysis were obtained by slow evaporation of a chloroform solution of **1**, yielding pale-yellow crystals of **1·C**. Alternatively, crystals of **1·C** can

also be obtained from a hot chloroform solution. Yellow crystals of the bromoform solvate **1·B** were prepared analogously using bromoform.

Recrystallization of **1** from hot methanol or gas-phase diffusion of diethyl ether into methanol afforded transparent crystals of the methanol solvate **1·M**. Soaking either **1** or **1·C** in acetonitrile produced selectively yellow crystalline material of **1·N**. Alternatively, gas-phase diffusion of diethyl ether into acetonitrile solution of **1** afforded yellow crystals of the acetonitrile solvate **1·N**. Boiling **1·N** in acetonitrile for 5 minutes yielded the **1·N^{o}** phase, typically with residual **1·N** present (ca. 5–10 %). For spectroscopy measurements, the **1·N^{o}** phase was manually separated by illuminating polycrystalline material (approx. 5 mg). Alternatively, single crystals of **1·N^{o}** can be obtained as a minor component (ca. 1–5%) alongside **1·N** via gas-phase diffusion of diethyl ether into a diluted acetonitrile solution of **1**, followed by manual separation.

Recrystallization of **1** from hot water yielded transparent crystals of the hydrate **1·W**. Direct boiling of **1** in acetone afforded the **1·A^{o}** phase selectively. In contrast, gas-phase diffusion of diethyl ether into an acetone solution of **1** produced a mixture of acetone solvates: predominantly **1·A**, with minor amounts (ca. 1–5%) of **1·A^{o}** and **1·A^{g}**. Soaking **1** or **1·C** in acetone selectively yielded the **1·A** form.

Exposure of **1·C** to small amounts of dichloromethane or 1,2-dichloroethane for 1–2 minutes resulted in emissive phases **1·D*** and **1·E**, appearing as a yellowish precipitate. Prolonged exposure to excess dichloromethane produced the non-emissive **1·D** form. This phase was also obtained via gas-phase diffusion of diethyl ether into a dichloromethane solution of **1**. Single crystals of **1·E** were prepared by recrystallization of **1** from hot 1,2-dichloroethane.

Bulk (micro)polycrystalline materials **1·C**, **1·N**, **1·A**, **1·E**, **1·W**, **1·M** can be obtained by dropping the corresponding solvent onto a dry diphosphonium salt **1**.

Solvent-free form **1·O** can be obtained by drying **1·N** and **1·A** at 100 °C for 30 minutes. Further heating under vacuum leads to thermally induced crystallization of the sample of unknown structure without any noticeable photoluminescence properties.

Photophysical Measurements: All solid-state photophysical measurements were performed under an inert atmosphere of nitrogen to minimize oxygen-induced quenching and, importantly, to suppress solvent loss from the solvent-containing crystalline phases. Particular care was taken during sample preparation and handling because the investigated solvatomorphs exhibit an intrinsic tendency toward gradual desolvation under ambient conditions.

For measurements of crystalline/microcrystalline powders, the samples were first treated by quick evaquation (10^{-3} mbar) – to nitrogen cycles (<2 min per 3 cycles) to remove adsorbed oxygen and then re-equilibrated with a few drops of the corresponding solvent under a nitrogen atmosphere to suppress possible solvent loss. The solvent was introduced immediately before the measurements, and the samples were maintained under the solvent vapor-containing atmosphere during the photophysical characterization. This procedure was adopted to minimize slow desolvation and the concomitant formation of solvent-deficient or amorphous material during sample preparation and measurement. For phases for which phase-pure single crystals (i.e., **1·N**, **1·W**, **1·E**, **1·A^{o}**, **1·M**) of sufficient quality and quantity were available, individual single crystals were used directly without vacuum drying. The use of solvent-containing conditions is particularly important for the present system because prolonged solvent removal or exposure to humidity can induce structural reorganization. TGA/DSC measurements show that desolvation is accompanied by subsequent thermal crystallization, while PXRD measurements demonstrate that prolonged drying can also result in the loss of the characteristic diffraction pattern for certain phases. Solvent-free form **1·O** was measured under a nitrogen atmosphere without solvent added.

Both excitation and emission spectra of the samples were recorded on an Edinburgh Instruments FLS1000 spectrometer, equipped with a 450 W ozone-free Xenon arc lamp, double monochromators for the excitation and emission pathways, and red-sensitive photomultipliers (PMT-980) as detectors. The excitation and emission spectra were corrected using the standard corrections supplied by the manufacturer for the spectral power of the excitation source and the sensitivity of the detector. The emission was collected at right angles to the excitation source with the emission wavelength selected using a double-grated monochromator and detected by the respective PMT. The quantum yields of the solid samples were measured by use of an integrating sphere in the presence of several drops of the solvent to keep the form homogeneous. Quantum yields at 77 K of crystalline **1·A⁰** and **1·N** were measured using Qunataurus-QY Hamamatsu. The luminescence lifetimes and time-resolved emission spectra were measured using an EP-LED pulsed laser diode (377 nm) with the multichannel scaling (MCS) method. A pulsed xenon microsecond flashlamp (60 W) was used to obtain long-lived (> 1 ms) lifetimes. Variable-temperature data measurements were performed with an Optistat DN cryostat at 77 K. Fluoracle and FAST spectrometer operating software and OriginPro 2025 10.2.0.188 were used for data analysis and processing.

Computational Details

All the calculations were performed at experimental geometries extracted from the single-crystal X-ray analysis data, with the position of solvent molecules and counterions the same as in the obtained crystals. The complete analysis of the electron density distribution function ρ(*r*) was performed by using Bader's *Atoms In Molecules* (AIM) theory[5] realized in AIMAll software. The reference electronic structure for AIM analysis was obtained by the single-point B3LYP/DGDZVP[6–8] method (DGDZVP is a full-electron basis set). The basic parameters we have analysed by AIM theory are: 1) electron density ρ(*r*) in the (3, –1) critical points of non-covalent interactions involving I^- counterions; 2) Laplacian $\nabla^2\rho(r)$ in these critical points; 3) potential *v*(*r*), kinetic *g*(*r*) and electron $h_e(r)$ energy densities in these critical points; 4) ellipticity (ε) of corresponding non-covalent interactions indicating the stability of the (3, –1) with respect to bifurcation; 5) delocalization index of electron density (DI) between the interacting atoms which indicates the number of electrons in the interatomic space; 6) energy of these intermolecular interactions based on Espinosa relation:[9] $E[\text{au}] = 1/2v(r) \sim E[\text{kcal/mol}] = 313.754v(r)$. For all detected and analysed non-covalent interactions, we have confirmed that $\nabla^2\rho(r) > 0$, and $|v(r)| < g(r)$, which results in $h_e(r) > 0$ and overall confirms all these bonds as interactions of closed electronic shells in terms of AIM formalism. This is also in agreement with relatively small DI values (typically below 0.1). Most of the analysed bonds demonstrate small ellipticity (below 0.3 in general), indicating dynamic stability of these interactions.

The relative energies of **1·A** and **1·A⁰** were calculated based on crystal structures obtained by single-crystal diffraction, without minimization of their energy. The excited state calculations were performed using the TDDFT method with the wB97XD/DGDZVP[8,10]method realized in Gaussian16 software. The spin-orbit coupling (SOC) matrix elements were calculated as root mean squares, *i.e.*, as the square root of the sum of squares of spin-orbit coupling matrix elements of all triplet state sublevels of the uncoupled states. The spin-orbit coupling operator was considered in our calculations within the zeroth-order regular approximation (ZORA).[11,12] These calculations were carried out using the ADF2026.104 package (ADF 2026.1, SCM, Theoretical Chemistry, Vrije Universiteit, Amsterdam, The Netherlands, https://www.scm.com.)[13]

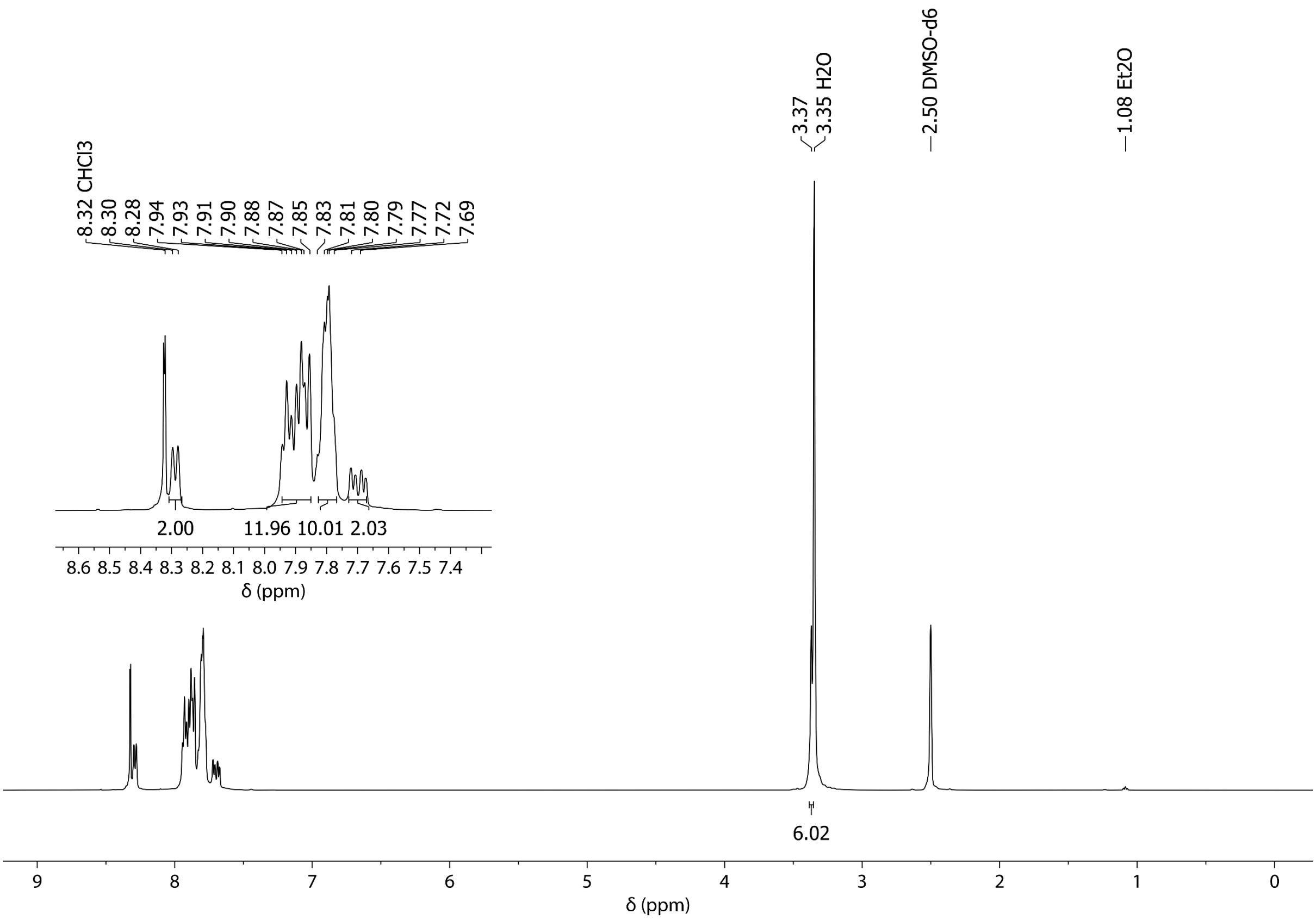

Figure S1. ^{1}H NMR spectrum of **1·C** (DMSO-d_6, 297 K, 500 Hz). Water and methyl protons are submerged at 3.30 ppm.

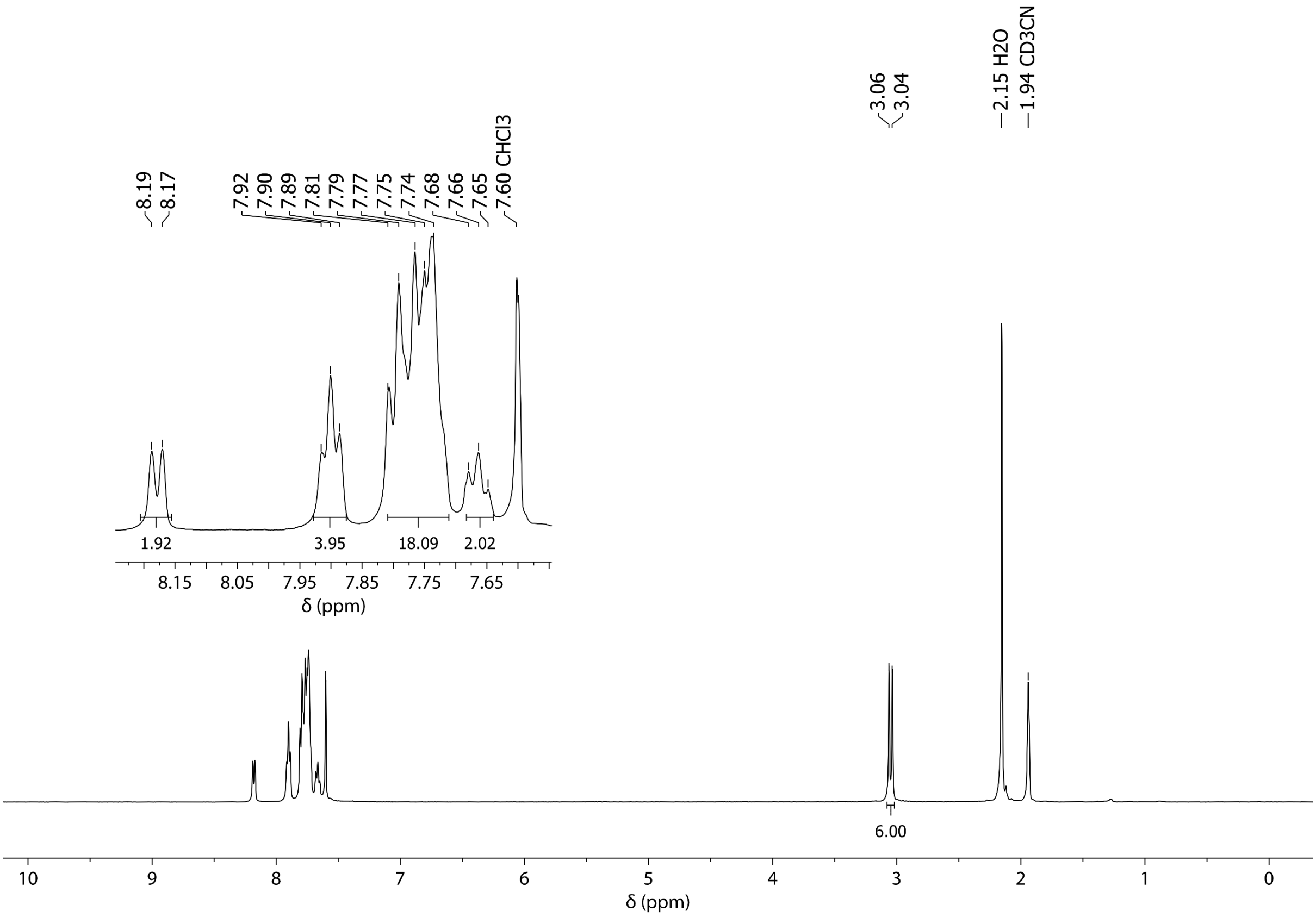

Figure S2. ^{1}H NMR spectrum of **1·C** (acetonitrile-d_3, 297 K, 500 Hz).

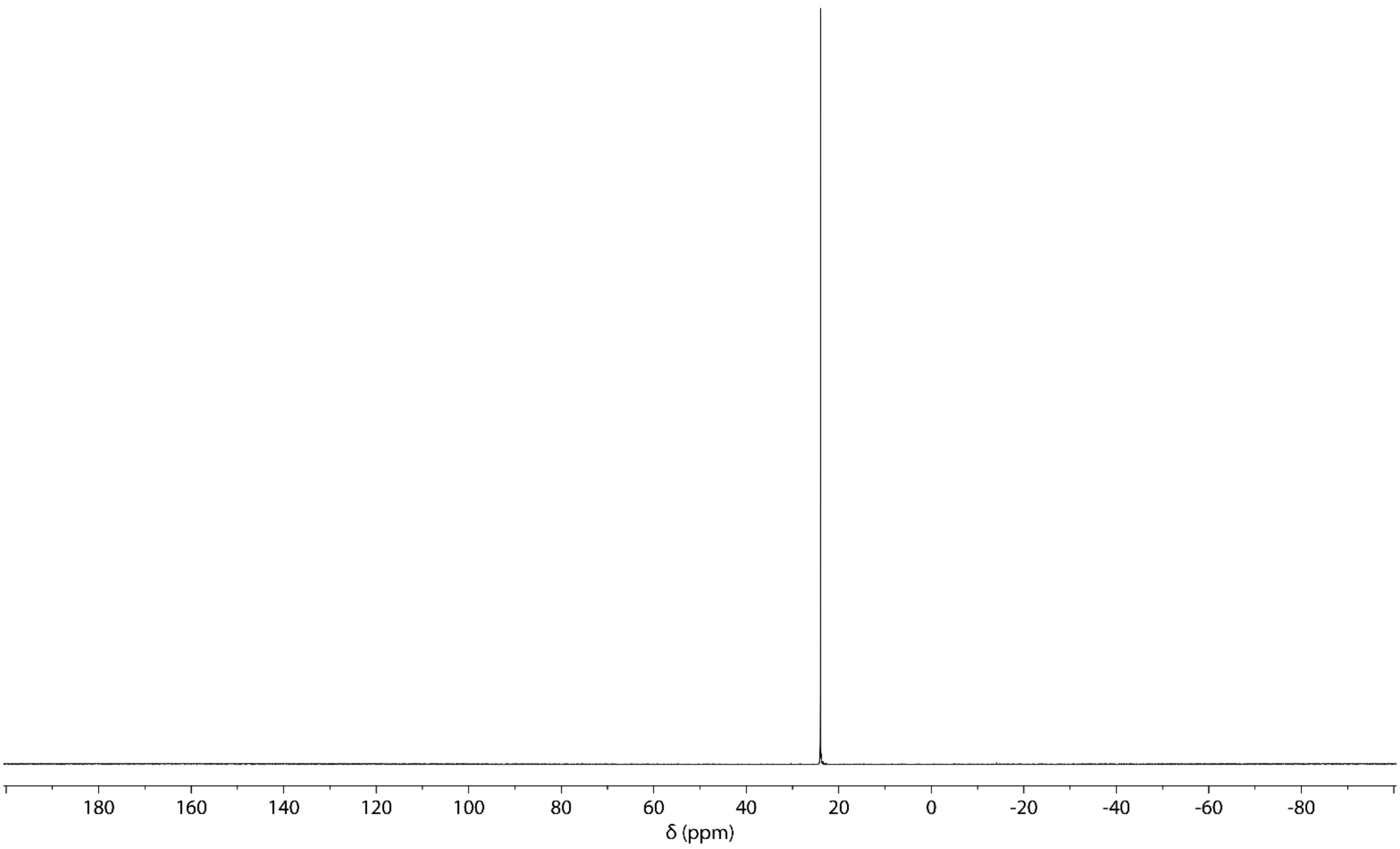

Figure S3. $^{31}P\{^1H\}$ NMR spectrum of **1·C** (DMSO-d_6, 297 K, 202 Hz).

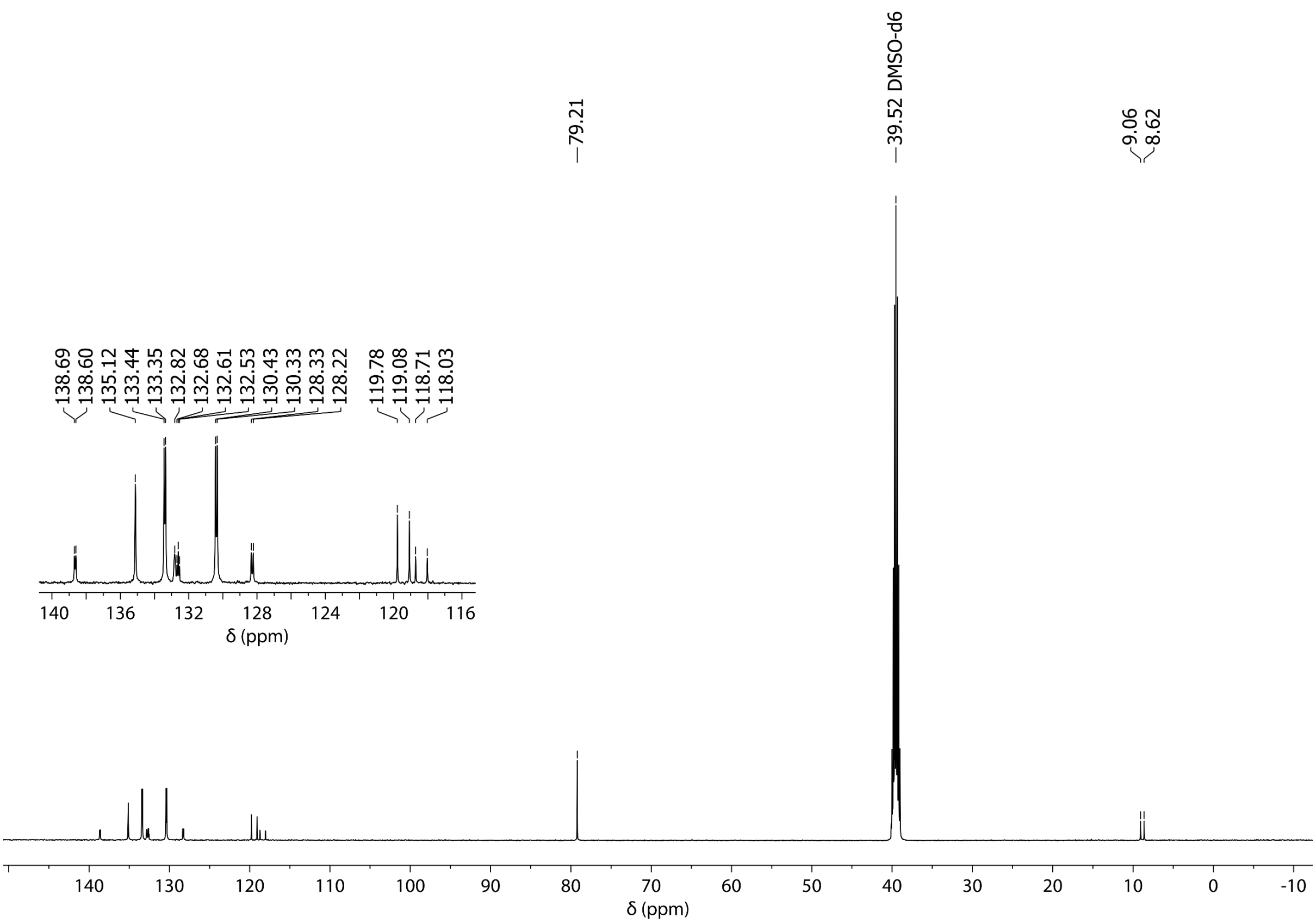

Figure S4. $^{13}C\{^1H\}$ NMR spectrum of **1·C** (DMSO-d_6, 297 K, 127 Hz). Solvent at 39.52 ppm.

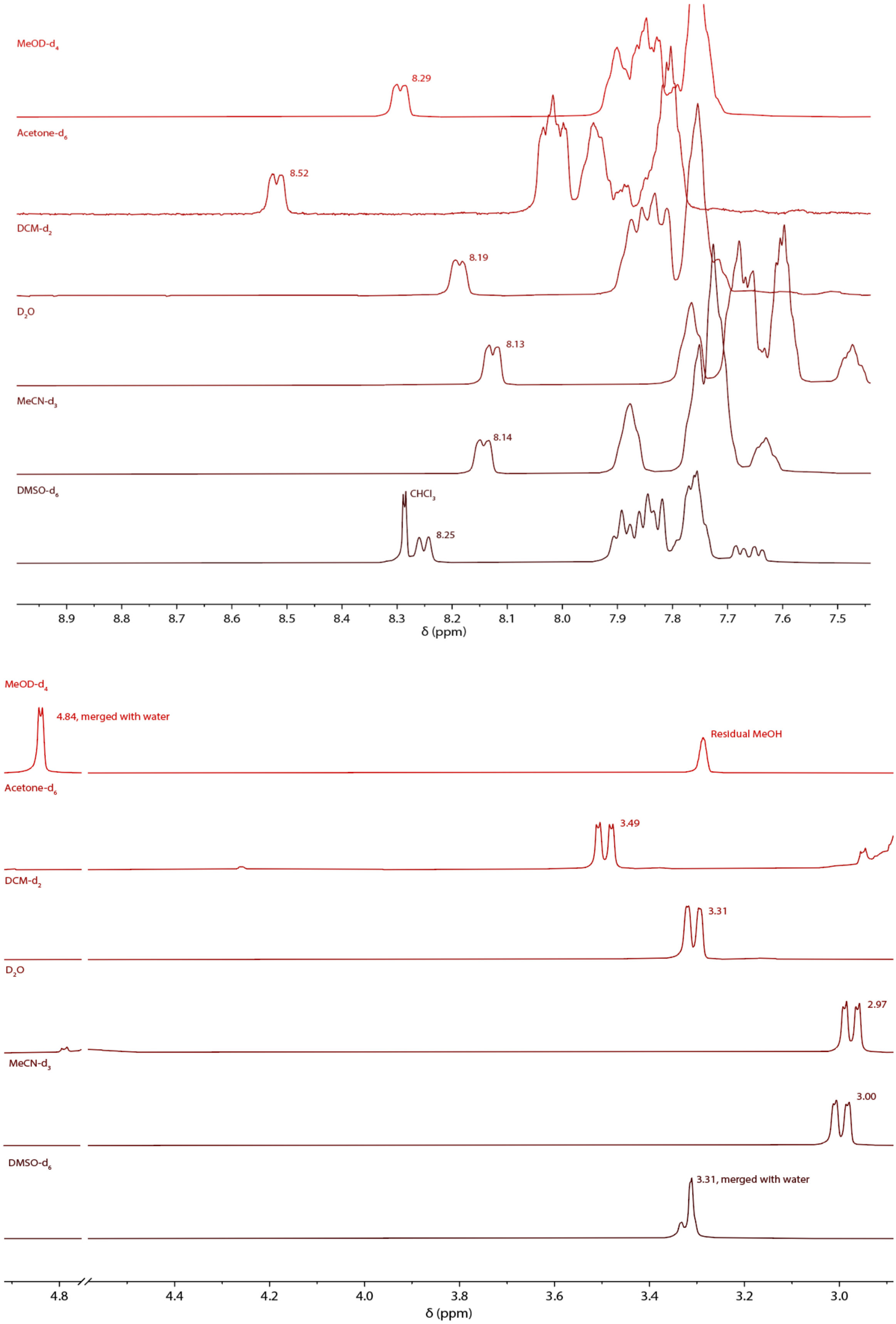

Figure S5. ^{1}H NMR spectra of **1** in different deuterated solvents.

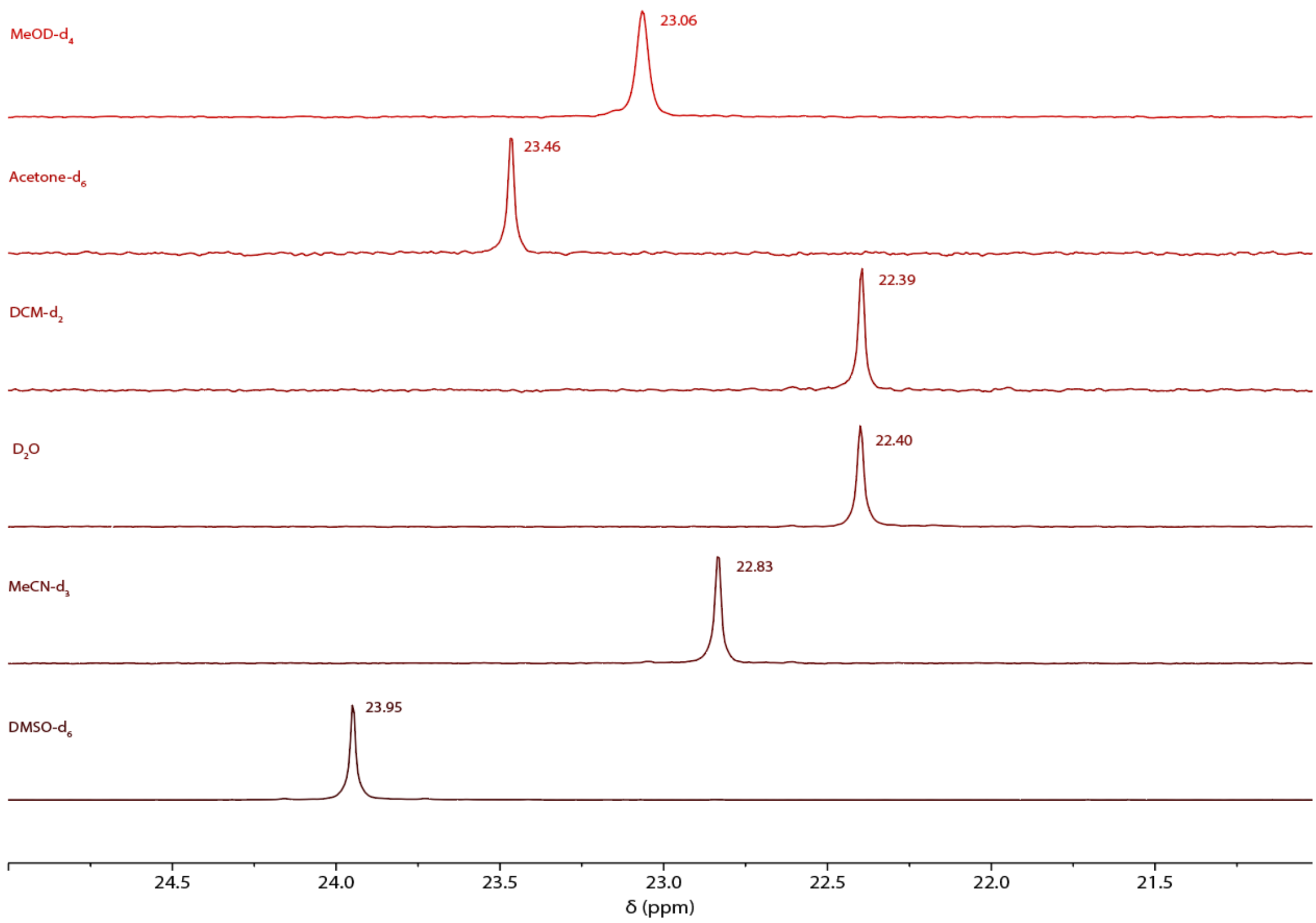

Figure S6. $^{31}P\{^1H\}$ NMR spectra of **1** in different deuterated solvents.

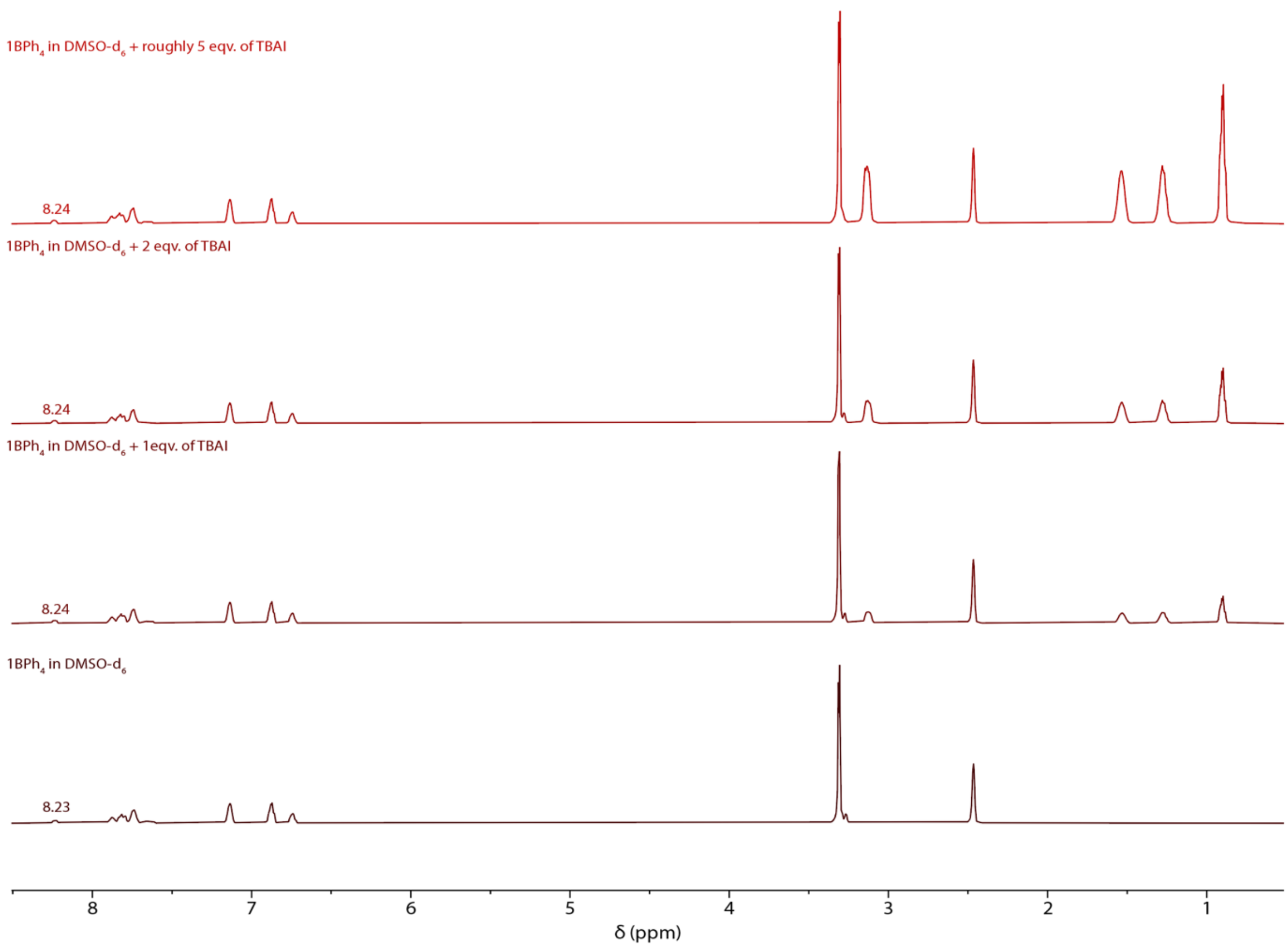

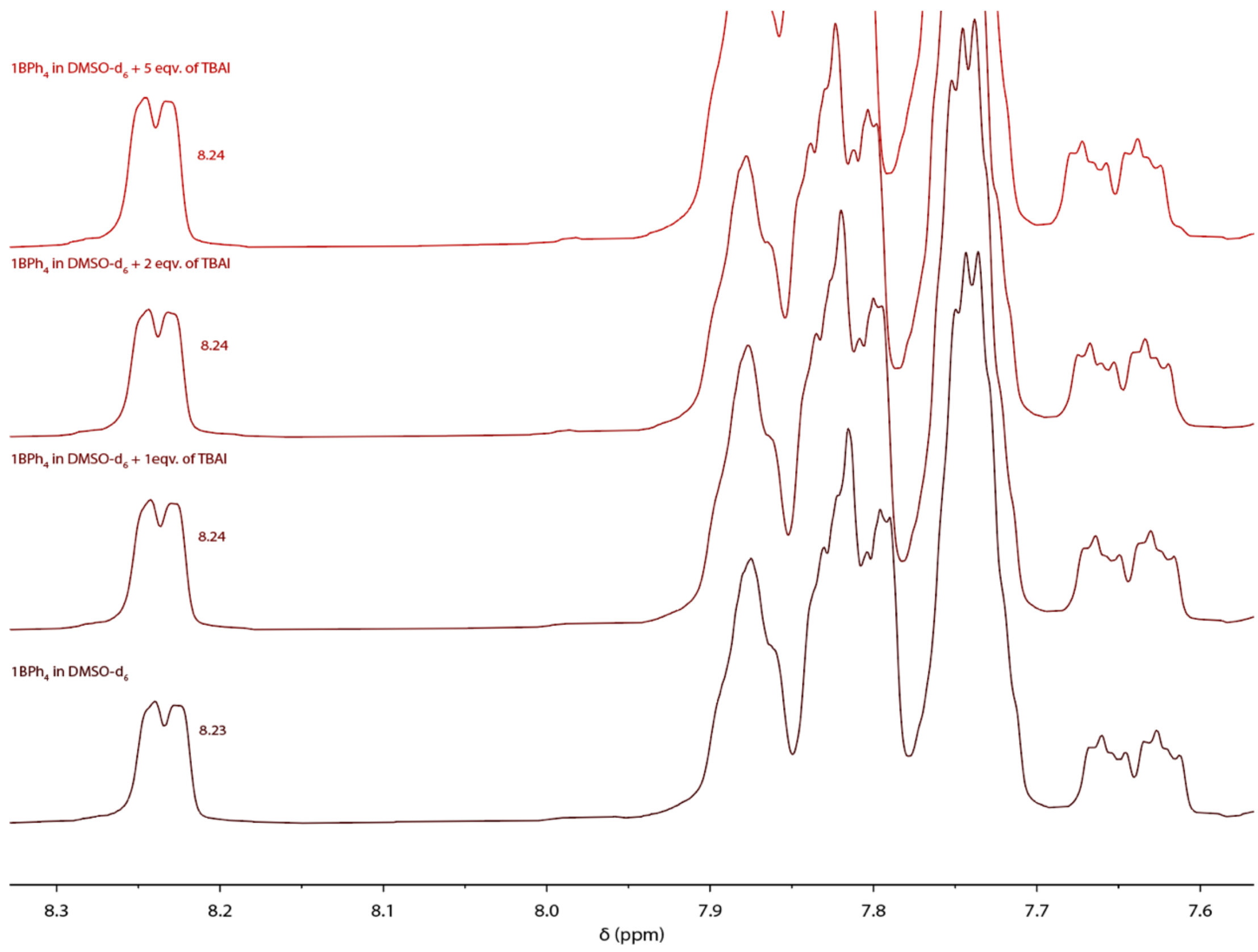

Figure S7. ^{1}H NMR titration experiment of $\mathbf{1}^{\mathbf{BPh4}}$ with tetrabutylammonium iodide(TBAI).

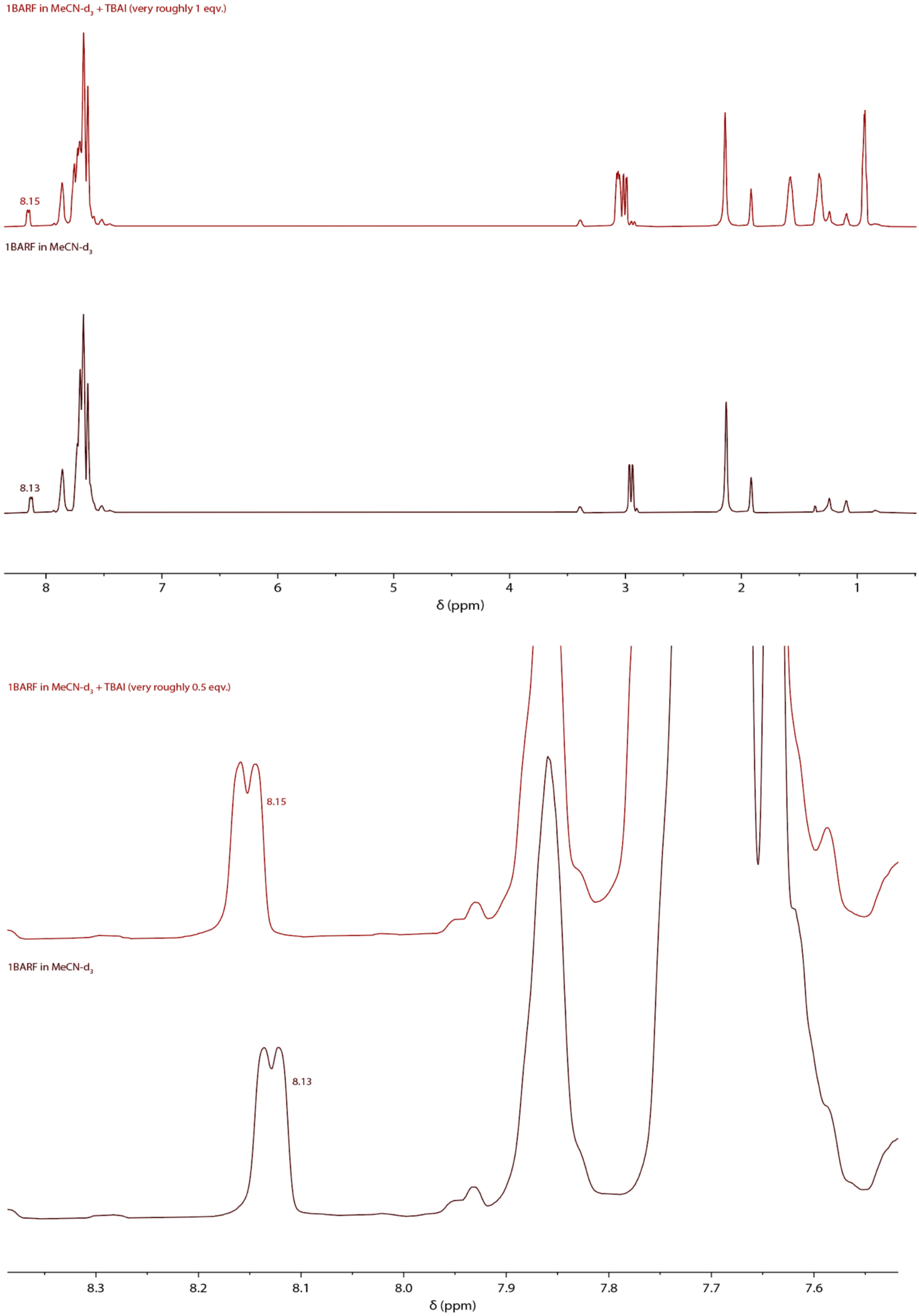

Figure S8. ^{1}H NMR titration experiment of $\mathbf{1}^{\mathbf{BARF}}$ with tetrabutylammonium iodide(TBAI).

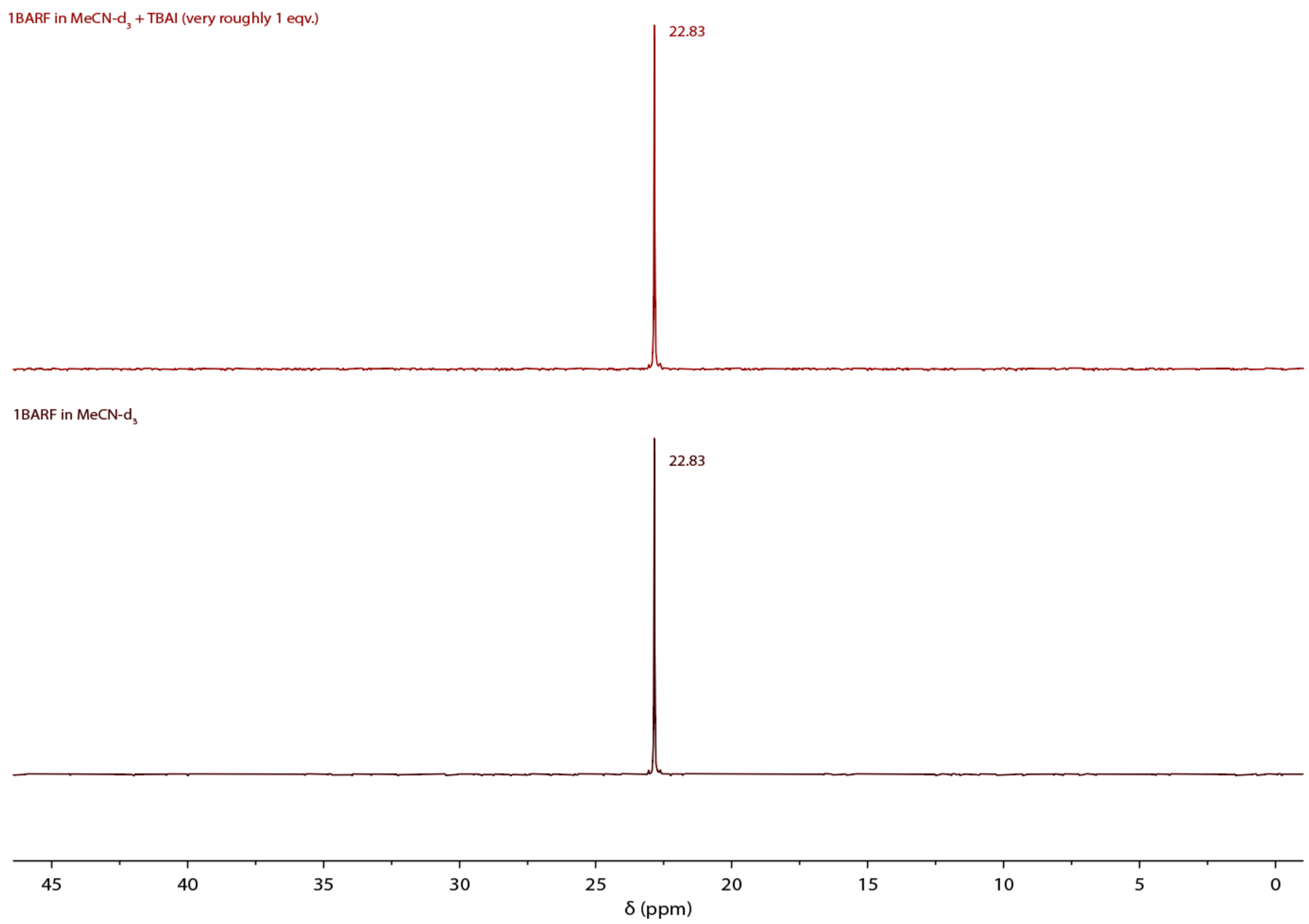

Figure S9. $^{31}P\{^{1}H\}$ NMR titration experiment of $\mathbf{1}^{\mathbf{BARF}}$ with tetrabutylammonium iodide(TBAI).

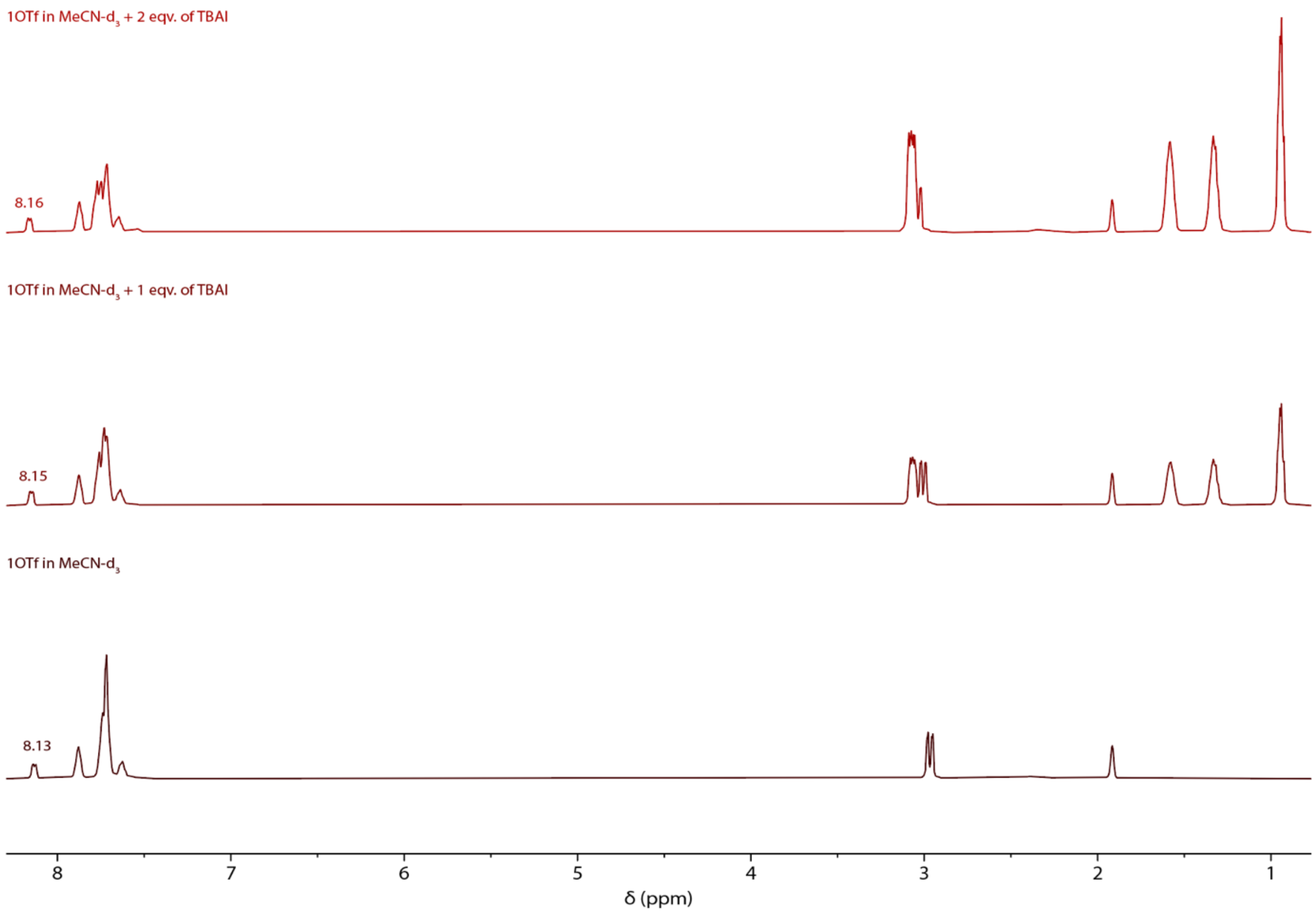

Figure S10. ^{1}H NMR titration experiment of $\mathbf{1}^{\mathbf{OTf}}$ with tetrabutylammonium iodide(TBAI).

NMR titration experiments: To probe possible solvent effects, ^1H and $^{31}P\{^1H\}$ NMR spectra of **1** were recorded in deuterated solvents corresponding to the different solvatomorphs (CD_3CN, D_2O, CD_2Cl_2, acetone-d_6, and CD_3OD; Figures S5, S6). The ^1H NMR spectra showed solvent-dependent changes, with the principal differences limited to chemical shifts of the naphthalene α-protons and methyl groups. The $^{31}P\{^1H\}$ NMR spectra exhibited primarily solvent-dependent chemical-shift variations without the appearance of new spectroscopic features. The interaction of $\mathbf{1^{OTf}}$ and $\mathbf{1^{BPh_4}}$ with iodide was further examined by titration with 1–5 equiv of TBAI in CD_3CN and DMSO-d_6, respectively. Addition of TBAI caused only a small shift (0.03 ppm) of the naphthalene α-proton resonance for $\mathbf{1^{OTf}}$, reflecting on in-plane H–I interactions, while no appreciable shift was observed for $\mathbf{1^{BPh_4}}$.

Interpretation of these solution-phase experiments is complicated by the limited solubility of **1** in weakly coordinating solvents, which decreases further upon TBAI addition and frequently results in precipitation or crystallization rather than a homogeneous titration. In more polar or coordinating solvents, rapidly exchanging contact, partially solvated, and fully solvated ion pairs may coexist, generating solvent-, concentration-, and temperature-dependent equilibria that prevent unambiguous assignment of individual chemical-shift changes to a specific ion-pairing configuration. Thus, while solvent-dependent NMR responses were observed, the combined effects of solvation, limited solubility, and TBAI-induced crystallization preclude a definitive assignment of the observed spectral changes to a specific iodide-association process.

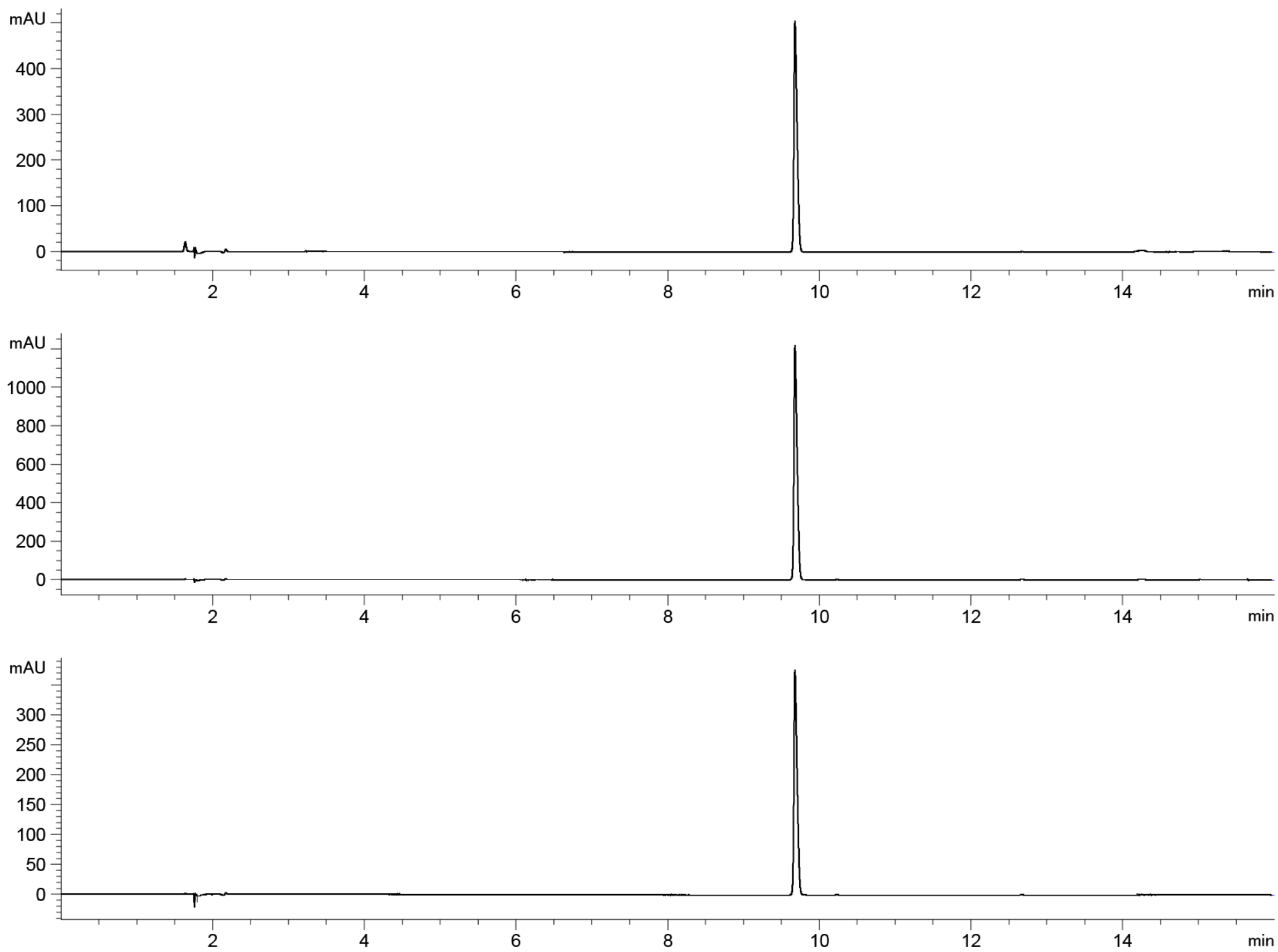

Figure S11. HPLC chromatograms of **1** (from top to bottom: monitored at 260 nm, 290 nm, and 320 nm). The injection peak is visible at 1.8 min.

Table S1. Crystal data and structure refinement for **1·C**, **1·N**, **1·A**, **1·N^o^**, and **1·A^g^**.

Identification code	**1·C**	**1·N**	**1·A**	**1·N^o^**	**1·A^g^**
CCDC number	2542718	2542719	2542720	2542721	2542722
Empirical formula	$C_{40}H_{36}Cl_{12}I_2P_2$	$C_{40}H_{38}I_2N_2P_2$	$C_{42}H_{44}I_2O_2P_2$	$C_{38}H_{35}I_2NP_2$	$C_{42}H_{44}I_2O_2P_2$
Formula weight	1257.82	862.46	896.50	821.41	896.51
Temperature [K]	150	150	150	150	150
Crystal system	monoclinic	triclinic	monoclinic	orthorhombic	monoclinic
Space group	*P*2~1~/*c*	*P*-1	*P*2~1~/*n*	*Pbca*	*Pn*
a [Å]<br>b [Å]<br>c [Å]<br>α [°]<br>β [°]<br>γ [°]	10.15711(8)<br>13.32122(12)<br>18.80962(16)<br>90<br>98.8181(8)<br>90	7.42100(10)<br>11.4431(3)<br>12.0586(3)<br>100.085(2)<br>99.268(2)<br>102.719(2)	7.5025(3)<br>23.4161(7)<br>11.5621(4)<br>90<br>101.736(3)<br>90	13.56080(9)<br>21.41071(17)<br>24.55519(18)<br>90<br>90<br>90	8.88630(10)<br>10.09020(10)<br>21.9927(2)<br>90<br>91.5820(10)<br>90
Volume [Å^3]	2514.96(4)	961.97(4)	1988.76(12)	7129.51(9)	1971.21(3)
Z	2	1	2	8	2
ρ_{calc} [g/cm^3]	1.661	1.489	1.497	1.531	1.510
μ [mm^{-1}]	16.514	13.839	13.433	14.898	13.552
F(000)	1232.0	428.0	896.0	3248.0	896.0
Crystal size [mm^3]	0.18 × 0.12 × 0.08	0.187 × 0.050 × 0.038	0.182 × 0.163 × 0.143	0.21 × 0.097 × 0.034	0.109 × 0.098 × 0.079
Radiation type	Cu Kα (λ = 1.54184)				
2Θ range for data collection [°]	8.166 to 159.928	7.612 to 159.728	7.55 to 159.092	7.2 to 159.926	8.044 to 160.284
Index ranges	-12 ≤ h ≤ 11, -16 ≤ k ≤ 15, -23 ≤ l ≤ 24	-9 ≤ h ≤ 9, -10 ≤ k ≤ 14, -15 ≤ l ≤ 14	-9 ≤ h ≤ 9, -26 ≤ k ≤ 29, -14 ≤ l ≤ 14	-7 ≤ h ≤ 16, -26 ≤ k ≤ 27, -27 ≤ l ≤ 30	-11 ≤ h ≤ 11, -12 ≤ k ≤ 12, -27 ≤ l ≤ 25
Reflections collected	21958	13683	15225	33399	78442
Independent reflections	5328 [R_{int} = 0.0482, R_{sigma} = 0.0394]	4080 [R_{int} = 0.0571, R_{sigma} = 0.0447]	4200 [R_{int} = 0.0599, R_{sigma} = 0.0485]	7553 [R_{int} = 0.0407, R_{sigma} = 0.0343]	8247 [R_{int} = 0.0588, R_{sigma} = 0.0257]
Data/restraints/parameters	5328/54/276	4080/0/211	4200/0/220	7553/56/420	8247/2/439
Goodness-of-fit on F^2 [(a)]	1.044	1.056	1.077	1.031	1.075
Final R indexes [I>=2σ (I)] [(b)]	R_1 = 0.0371, wR_2 = 0.0977	R_1 = 0.0466, wR_2 = 0.1242	R_1 = 0.0435, wR_2 = 0.1140	R_1 = 0.0316, wR_2 = 0.0788	R_1 = 0.0452, wR_2 = 0.1216
Final R indexes [all data] [(b)]	R_1 = 0.0385, wR_2 = 0.0989	R_1 = 0.0471, wR_2 = 0.1249	R_1 = 0.0454, wR_2 = 0.1154	R_1 = 0.0355, wR_2 = 0.0809	R_1 = 0.0456, wR_2 = 0.1221
Largest diff. peak/hole [e/Å^{-3}]	0.81/-2.36	2.46/-2.14	1.27/-1.44	1.19/-0.88	0.95/-0.69
Flack parameter	–	–	–	–	0.001(6)

[(a)] GooF = S = $[[\sum w(F_o^2-F_c^2)^2]/(m-n)]^{1/2}$, where m = number of reflexes and n = number of parameters; [(b)] $R_1 = \sum||F_o|-|F_c||/\sum|F_o|$; $wR_2 = [\sum[w(F_o^2-F_c^2)^2/\sum[(wF_o^2)^2]]^{1/2}$; $w = 1/[\sigma^2(F_o^2)+(aP)^2+bP]$, where $P = (F_o^2+2F_c^2)$

Table S2. Crystal data and structure refinement for **1·B**, **1·W, 1·M, 1·E**, and **1·D**.

Identification code	**1·B**	**1·W**	**1·M**	**1·E**	**1·D**
CCDC number	2542723	2542724	2542732	2542725	2542726
Empirical formula	$C_{40}H_{36}Br_{12}I_2P_2$	$C_{36}H_{36}I_2O_2P_2$	$C_{38.84}H_{43.34}I_2O_{2.83}P_2$	$C_{38}H_{36}Cl_2I_2P_2$	$C_{38}H_{36}Cl_4I_2P_2$
Formula weight	1791.23	816.39	871.19	879.31	950.20
Temperature [K]	150	150	150	100	150
Crystal system	monoclinic	triclinic	triclinic	orthorhombic	monoclinic
Space group	*P*2$_1$/*c*	*P*-1	*P*-1	*Pbca*	*P*2$_1$/*n*
a [Å]<br>b [Å]<br>c [Å]<br>α [°]<br>β [°]<br>γ [°]	10.39206(12)<br>13.60605(15)<br>18.6881(2)<br>90<br>99.8072(11)<br>90	8.7682(3)<br>10.3220(5)<br>10.4787(4)<br>72.449(4)<br>70.107(4)<br>88.086(3)	10.2273(2)<br>14.3427(5)<br>15.3087(6)<br>62.276(4)<br>85.548(2)<br>88.449(2)	13.69252(13)<br>21.17595(19)<br>24.6835(2)<br>90<br>90<br>90	10.7549(4)<br>15.2077(4)<br>12.8668(5)<br>90<br>105.333(4)<br>90
Volume [Å^3]	2603.78(5)	847.74(7)	1981.68(13)	7157.04(11)	2029.54(13)
Z	2	1	2	8	2
ρ_{calc} [g/cm^3]	2.285	1.599	1.460	1.632	1.555
μ [mm^{-1}]	21.075	15.692	13.478	16.217	15.527
F(000)	1664.0	404.0	870.0	3472.0	936.0
Crystal size [mm^3]	0.1 × 0.078 × 0.061	0.15 × 0.13 × 0.1	0.099 × 0.055 × 0.033	0.1 × 0.088 × 0.078	0.15 × 0.13 × 0.1
Radiation type	Cu Kα (λ = 1.54184)				
2Θ range for data collection [°]	8.08 to 160.278	9.012 to 158.74	6.962 to 160.422	7.162 to 153.882	9.198 to 159.13
Index ranges	-13 ≤ h ≤ 13, -16 ≤ k ≤ 17, -23 ≤ l ≤ 23	-9 ≤ h ≤ 11, -13 ≤ k ≤ 12, -13 ≤ l ≤ 13	-12 ≤ h ≤ 11, -18 ≤ k ≤ 18, -19 ≤ l ≤ 19	-7 ≤ h ≤ 17, -26 ≤ k ≤ 26, -30 ≤ l ≤ 31	-13 ≤ h ≤ 13, -18 ≤ k ≤ 19, -16 ≤ l ≤ 15
Reflections collected	22805	10398	29842	28117	15881
Independent reflections	5554 [R_{int} = 0.0423, R_{sigma} = 0.0363]	3520 [R_{int} = 0.0501, R_{sigma} = 0.0459]	8280 [R_{int} = 0.0508, R_{sigma} = 0.0482]	7299 [R_{int} = 0.0346, R_{sigma} = 0.0314]	4292 [R_{int} = 0.0597, R_{sigma} = 0.0455]
Data/restraints/parameters	5554/9/313	3520/0/194	8280/18/453	7299/0/399	4292/0/211
Goodness-of-fit on F^2 [(a)]	1.071	1.077	1.066	1.059	1.055
Final R indexes [I>=2σ (I)] [(b)]	R_1 = 0.0303, wR_2 = 0.0798	R_1 = 0.0366, wR_2 = 0.0981	R_1 = 0.0461, wR_2 = 0.1255	R_1 = 0.0304, wR_2 = 0.0816	R_1 = 0.0691, wR_2 = 0.1920
Final R indexes [all data] [(b)]	R_1 = 0.0324, wR_2 = 0.0811	R_1 = 0.0390, wR_2 = 0.1005	R_1 = 0.0526, wR_2 = 0.1305	R_1 = 0.0339, wR_2 = 0.0840	R_1 = 0.0723, wR_2 = 0.1960
Largest diff. peak/hole [e/Å^{-3}]	0.91/-0.84	1.22/-0.93	1.49/-1.21	1.22/-0.82	0.10/-0.04

[(a)] GooF = S = $[[\sum w(F_o^2-F_c^2)^2]/(m-n)]^{1/2}$, where m = number of reflexes and n = number of parameters; [(b)] $R_1 = \sum||F_o|-|F_c||/\sum|F_o|$; $wR_2 = [\sum[w(F_o^2-F_c^2)^2/\sum[(wF_o^2)^2]]^{1/2}$; $w = 1/[\sigma^2(F_o^2)+(aP)^2+bP]$, where $P = (F_o^2+2F_c^2)$

Table S3. Crystal data and structure refinement of single crystal **1·A°** at variable temperature.

Temperature	**100 K**	**150 K**	**200 K**	**270 K**
CCDC number	2542727	2542728	2542729	2542730
Empirical formula	$C_{39}H_{38}I_2OP_2$			
Formula weight	838.43			
Crystal system	orthorhombic			
Space group	*Pbca*			
a [Å]<br>b [Å]<br>c [Å]<br>α [°]<br>β [°]<br>γ [°]	13.77930(10)<br>20.6313(2)<br>24.9723(2)<br>90<br>90<br>90	13.80240(10)<br>20.6871(2)<br>25.0324(2)<br>90<br>90<br>90	13.82980(10)<br>20.7589(2)<br>25.0994(2)<br>90<br>90<br>90	13.87299(12)<br>20.8858(2)<br>25.1786(3)<br>90<br>90<br>90
Volume [Å^3]	7099.25(10)	7147.54(10)	7205.82(10)	7295.46(13)
Z	8	8	8	8
ρ_{calc} [g/cm^3]	1.569	1.558	1.546	1.527
μ [mm^{-1}]	14.987	14.886	14.765	14.584
F(000)	3328.0	3328.0	3328.0	3328.0
Crystal size [mm^3]	0.122 × 0.06 × 0.056			
Radiation type	Cu Kα (λ = 1.54184)			
2Θ range for data collection [°]	7.08 to 154.06	7.062 to 153.95	7.044 to 153.194	7.022 to 160.192
Index ranges	-12 ≤ h ≤ 17, -25 ≤ k ≤ 26, -28 ≤ l ≤ 31	-12 ≤ h ≤ 17, -25 ≤ k ≤ 26, -28 ≤ l ≤ 31	-12 ≤ h ≤ 17, -25 ≤ k ≤ 26, -27 ≤ l ≤ 31	-17 ≤ h ≤ 14, -26 ≤ k ≤ 22, -31 ≤ l ≤ 31
Reflections collected	27508	27737	28114	33740
Independent reflections	7256 [R_{int} = 0.0476, R_{sigma} = 0.0374]	7328 [R_{int} = 0.0478, R_{sigma} = 0.0395]	7336 [R_{int} = 0.0532, R_{sigma} = 0.0466]	7688 [R_{int} = 0.0593, R_{sigma} = 0.0508]
Data/restraints/parameters	7256/0/401	7328/0/401	7336/0/401	7688/24/432
Goodness-of-fit on F^2 [(a)]	1.044	1.038	1.037	1.069
Final R indexes [I>=2σ (I)] [(b)]	R_1 = 0.0291, wR_2 = 0.0732	R_1 = 0.0297, wR_2 = 0.0747	R_1 = 0.0362, wR_2 = 0.0952	R_1 = 0.0421, wR_2 = 0.1164
Final R indexes [all data] [(b)]	R_1 = 0.0315, wR_2 = 0.0747	R_1 = 0.0334, wR_2 = 0.0768	R_1 = 0.0408, wR_2 = 0.0985	R_1 = 0.0479, wR_2 = 0.1210
Largest diff. peak/hole [e/Å^{-3}]	0.60/-1.06	0.66/-1.03	0.74/-1.31	1.39/-0.84

[(a)] GooF = S = $[[\sum w(F_o^2-F_c^2)^2]/(m-n)]^{1/2}$, where m = number of reflexes and n = number of parameters; [(b)] $R_1 = \sum||F_o|-|F_c||/\sum|F_o|$; $wR_2 = [\sum[w(F_o^2-F_c^2)^2/\sum[(wF_o^2)^2]]^{1/2}$; $w = 1/[\sigma^2(F_o^2)+(aP)^2+bP]$, where $P = (F_o^2+2F_c^2)$

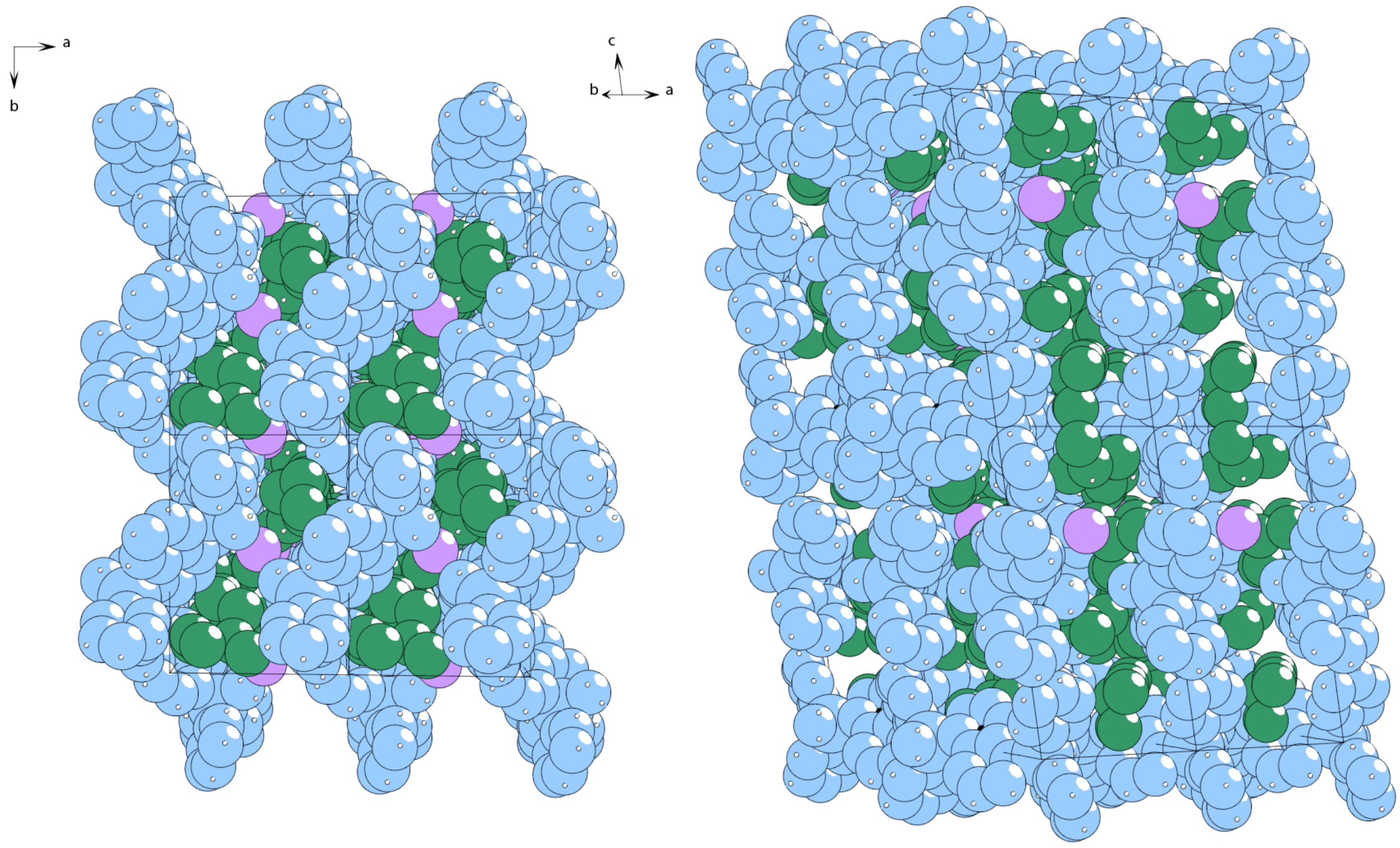

Figure S12. Packing representation of a 2×2×2-unit supercell of the **1·C** showing porous arrangement and existing voids of chloroform–iodide network around zig-zag oriented dications. Depicted in blue – diphsophonium cation, in green – chloroform molecules, in purple – iodides.

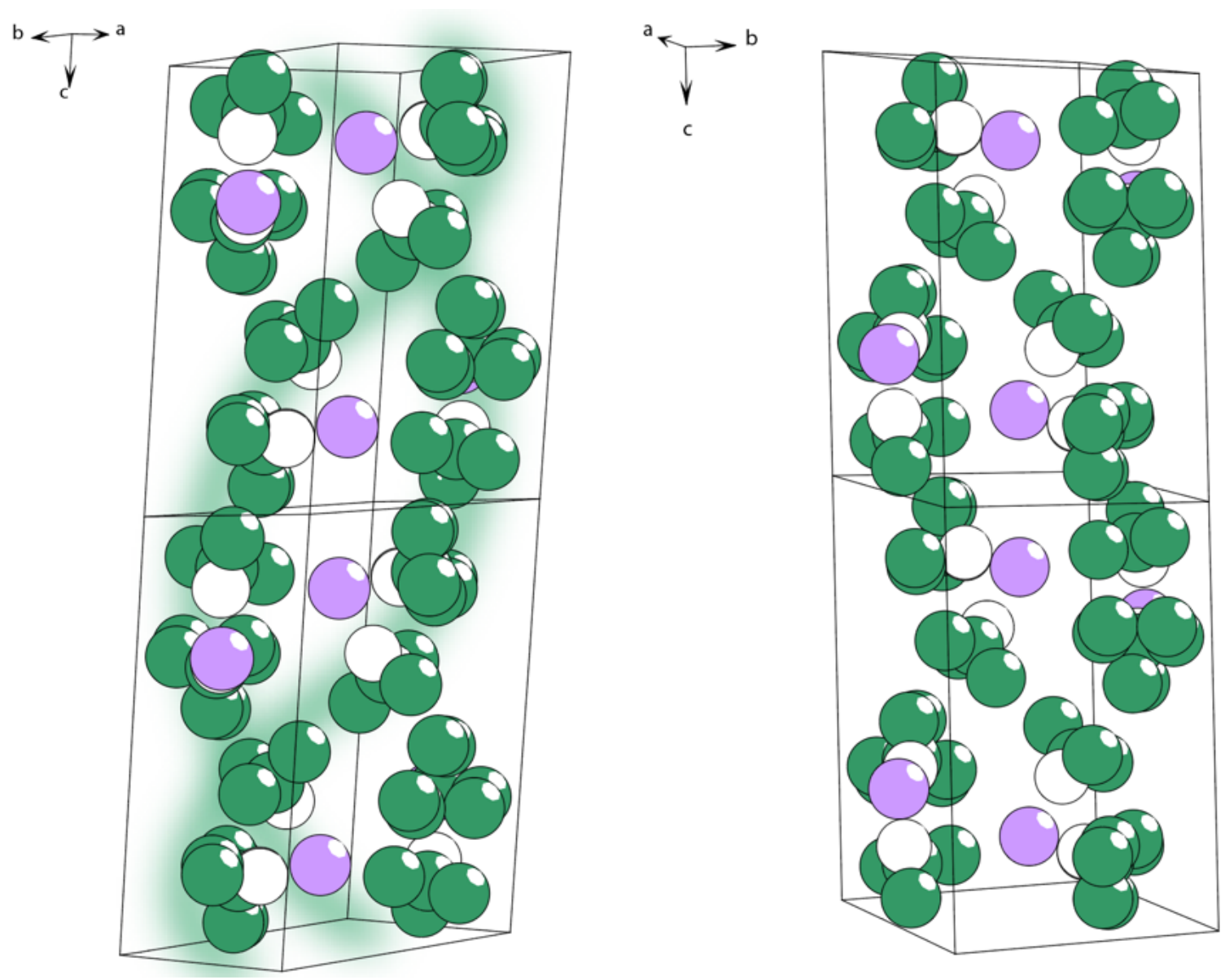

Figure S13. Packing fragment of the **1·C** (2×1×1-unit supercell) demonstrating chloroform–iodide chains formed via multiple C–H···Cl/C–H···I hydrogen bonds and Cl···Cl/Cl···I halogen contacts (diphosphonium cations are omitted for clarity).

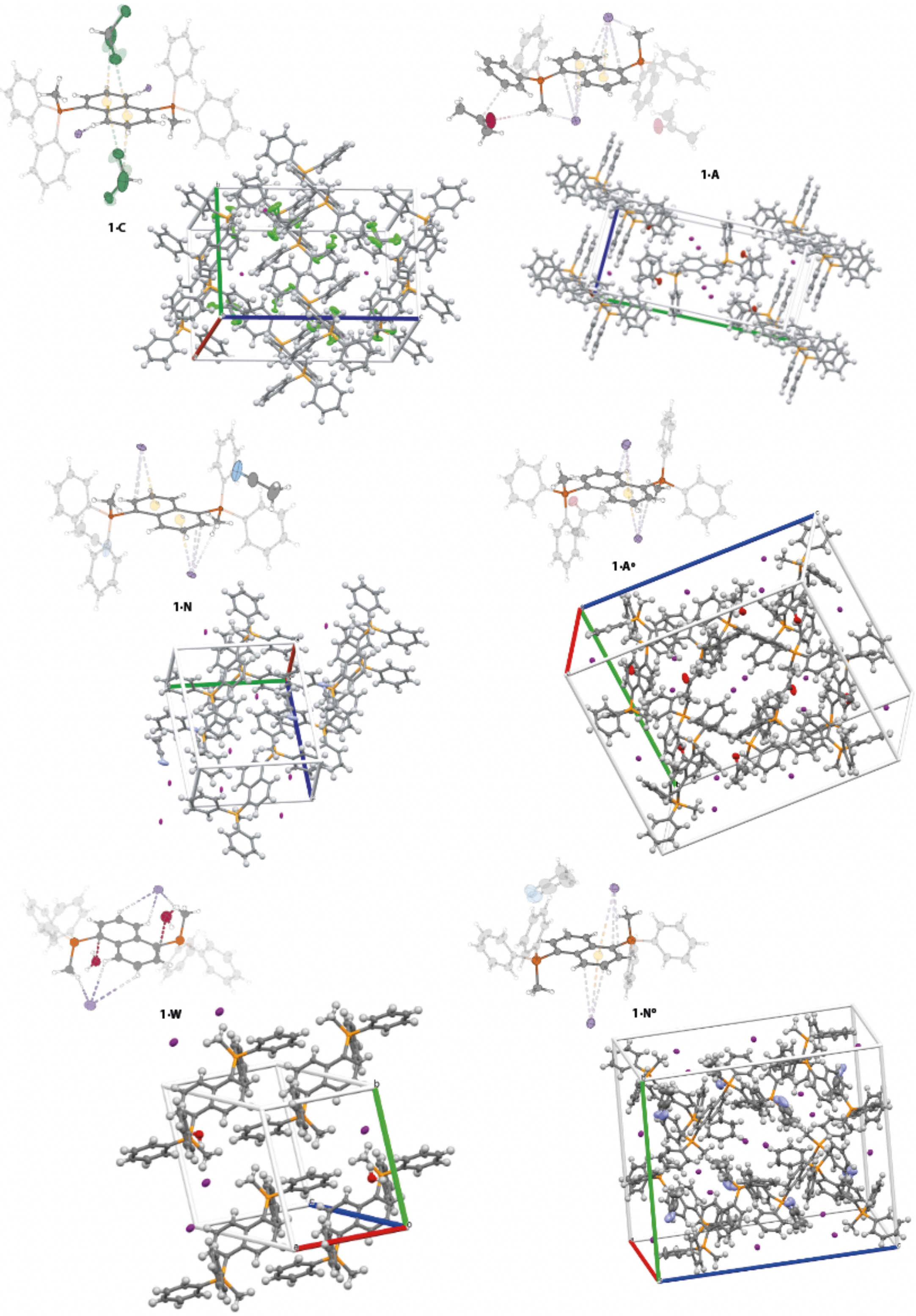

Figure S14. Discrete ion pairs and packing fragments of the **1·C**, **1·A**, **1·N**, **1·W**, **1·Aº** and **1·Nº**.

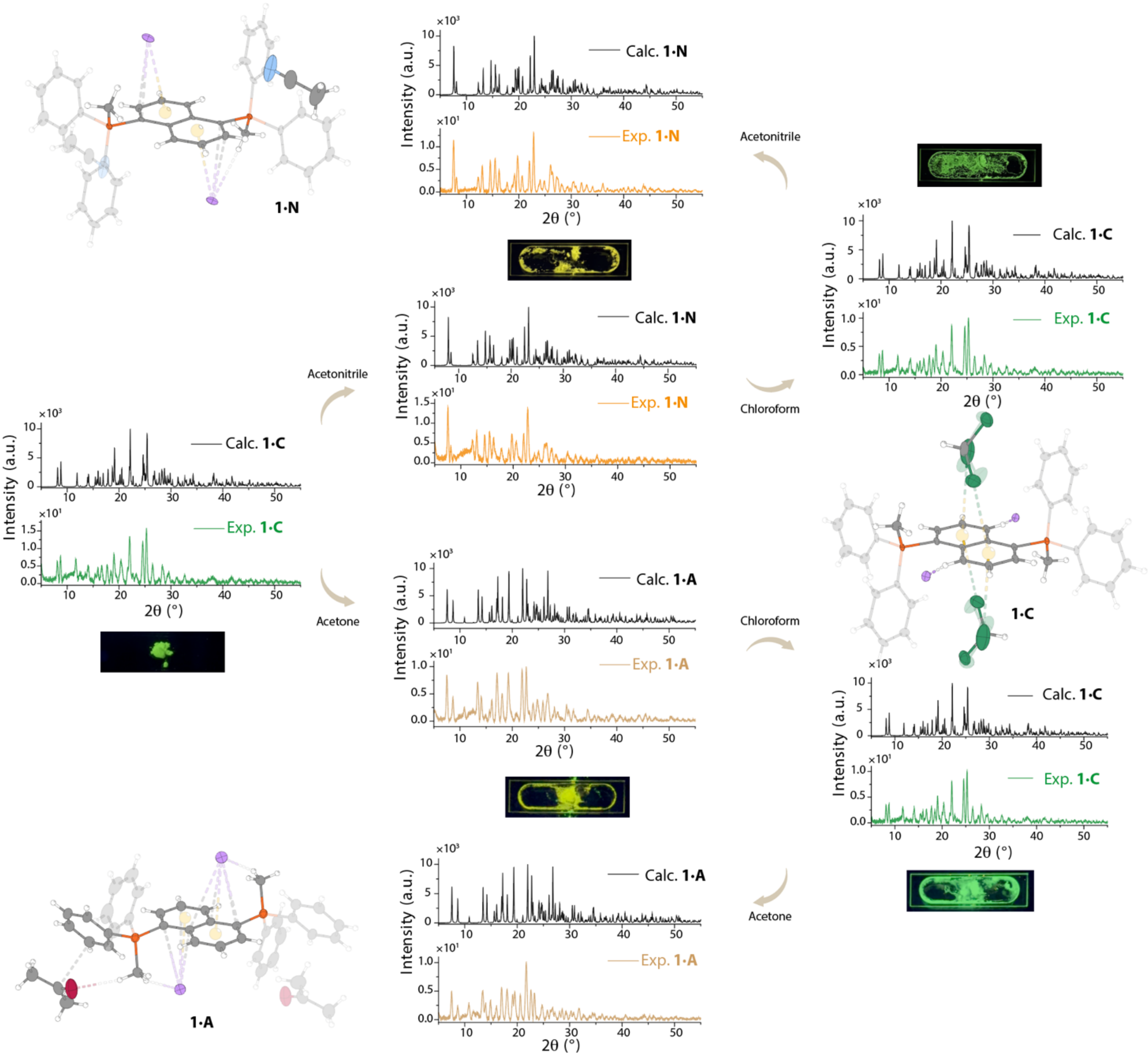

Figure S15. PXRD data of cyclic phase transition **1·C↔1·A** and **1·C↔1·N** showing reversible transformation.

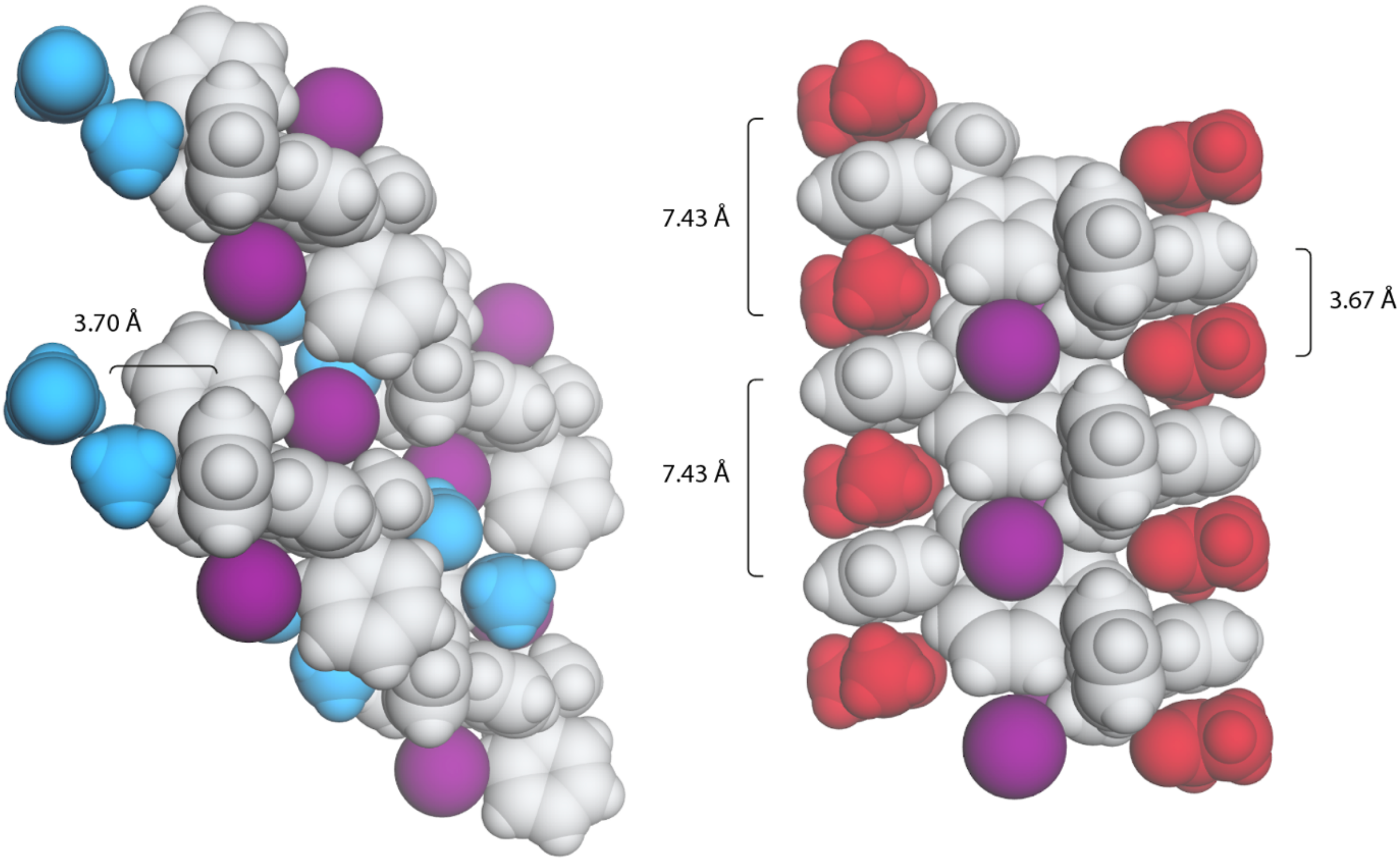

Figure S16. Packing of **1·N** (left) and **1·A** (right) demonstrating stepladder/columnar organization of diphosphonium fragment (in grey) with intercalated head-to-tail dimeric acetonitrile (blue)/acetone molecules (in red); iodides are depicted in purple.

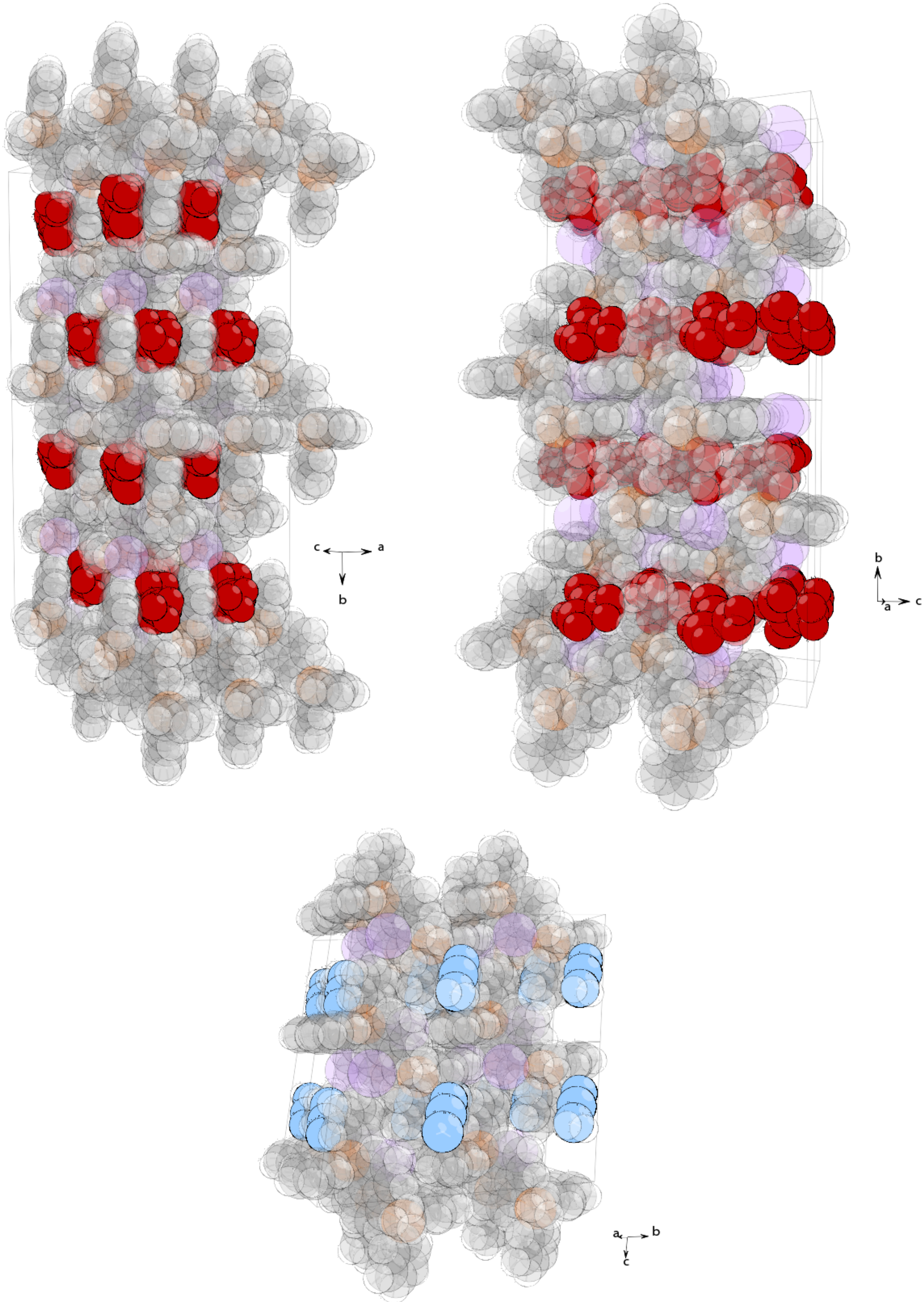

Figure S17. Packing representation of a 2×2×2-unit supercell of the **1·A** (up) and **1·N** (bottom), demonstrating solvent channels in **1·A**. Depicted in red – acetone molecules, in blue – acetonitrile molecules, and the rest with an opacity 30%.

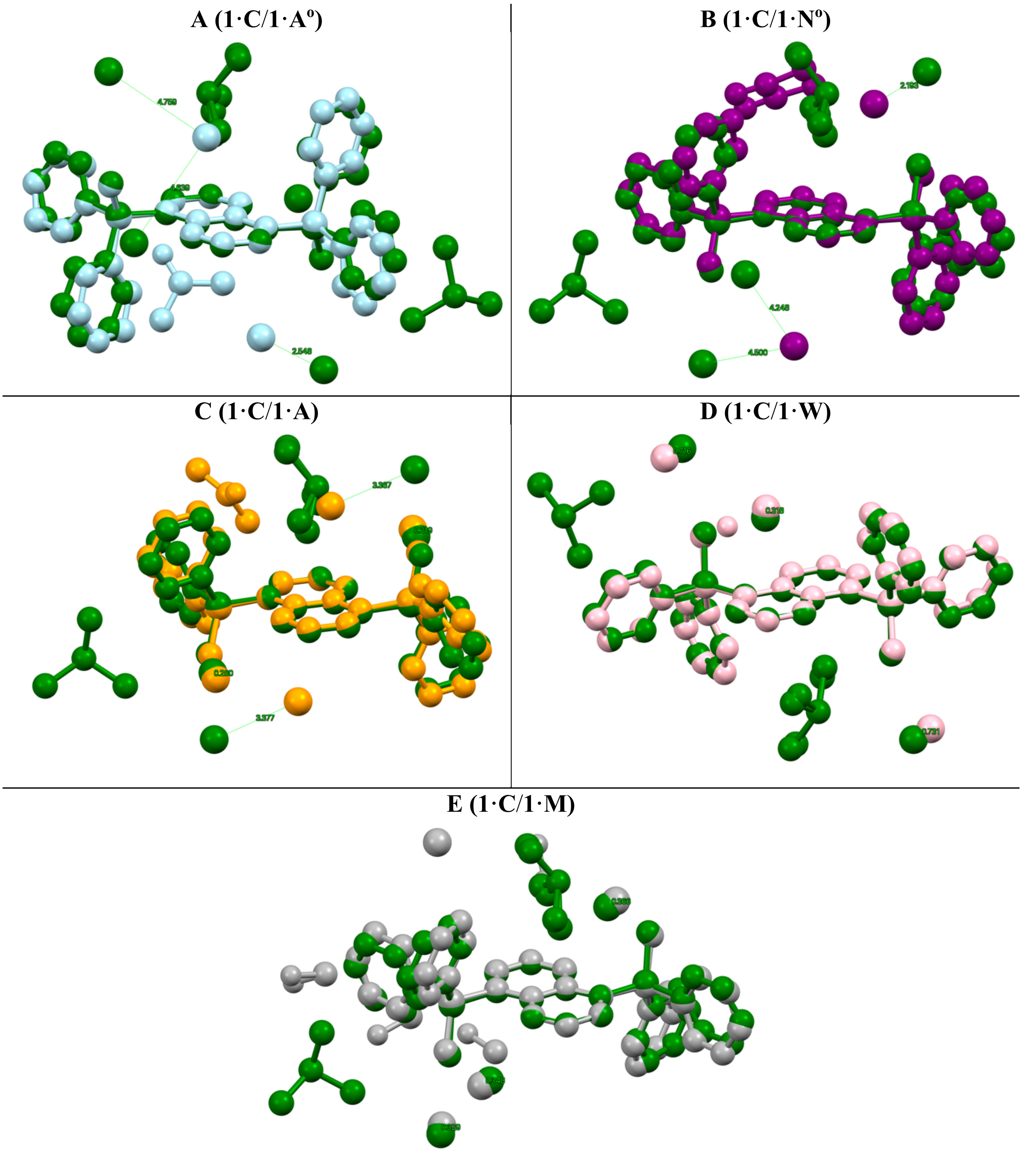

Overlay	$I^{1\cdot C}\cdots I^{1\cdot X}$ distance (1), Å	$I^{1\cdot C}\cdots I^{1\cdot X}$ distance (2), Å	$I^{1\cdot C}\cdots I^{1\cdot X}$ distance (3), Å
**1·C/1·A^o**	4.76	4.63	2.55
**1·C/1·N^o**	4.50	4.24	2.19
**1·C/1·A**	3.37/3.38	0.28/0.32	–
**1·C/1·W**	0.72	0.32	–
**1·C/1·M**	0.37	0.35	0.33

Figure S18. Overlay of the fragments of **1·C** (green)/**1·A^o** (blue) (A), **1·C** (green)/**1·N^o** (purple) (B), **1·C** (green)/**1·A** (orange) (C), **1·C** (green)/**1·W** (pink) (D), and **1·C** (green)/**1·M** (grey) (E); fixed at naphthalene atoms demonstrating possible distance trajectory of anion(I^-) translocation in the solid state (ball and stick representation).

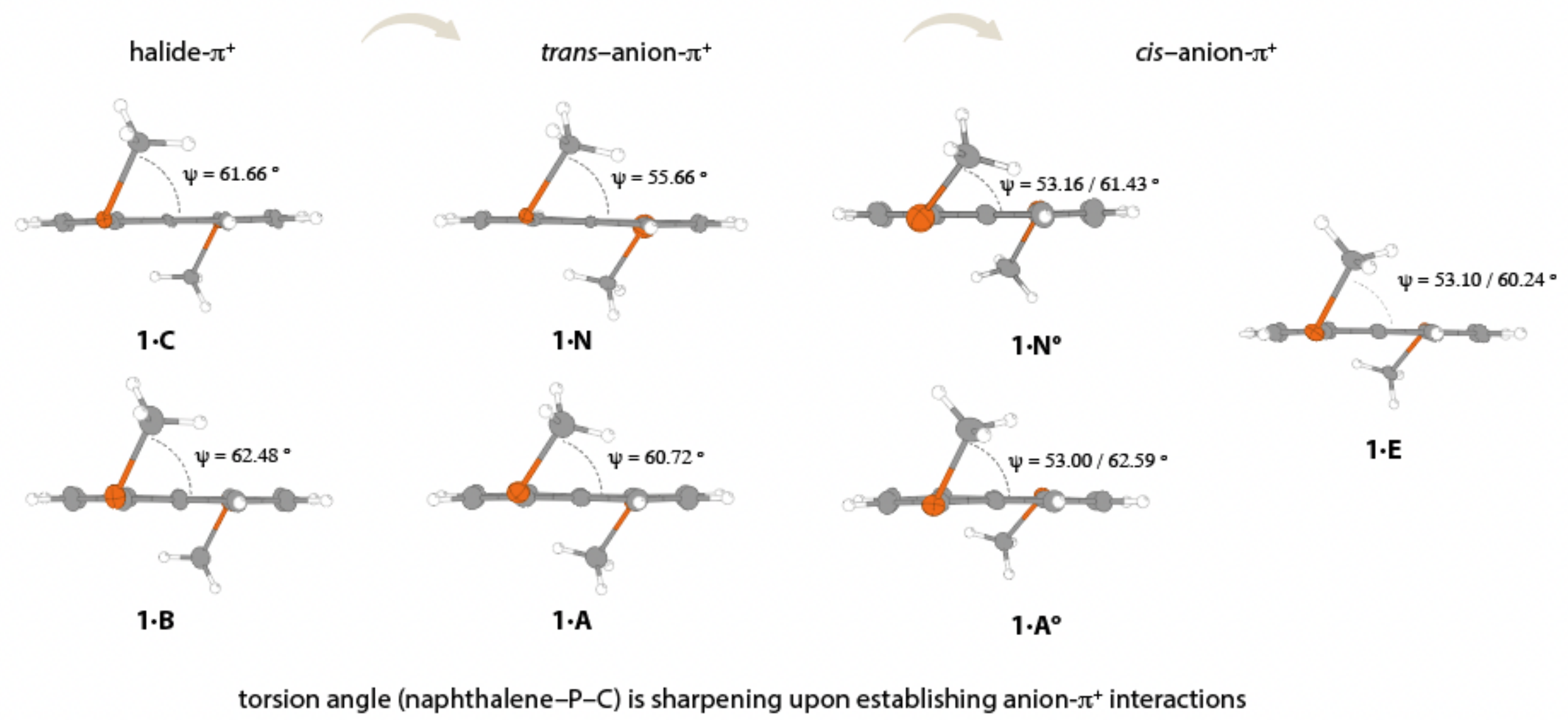

Figure S19. Fragments of the cations **1·C**, **1·B**, **1·N**, **1·A**, **1·N°**, **1·A°**, and **1·E** with depicted torsion angles between the naphthalene plane and P–CH_3 vector.

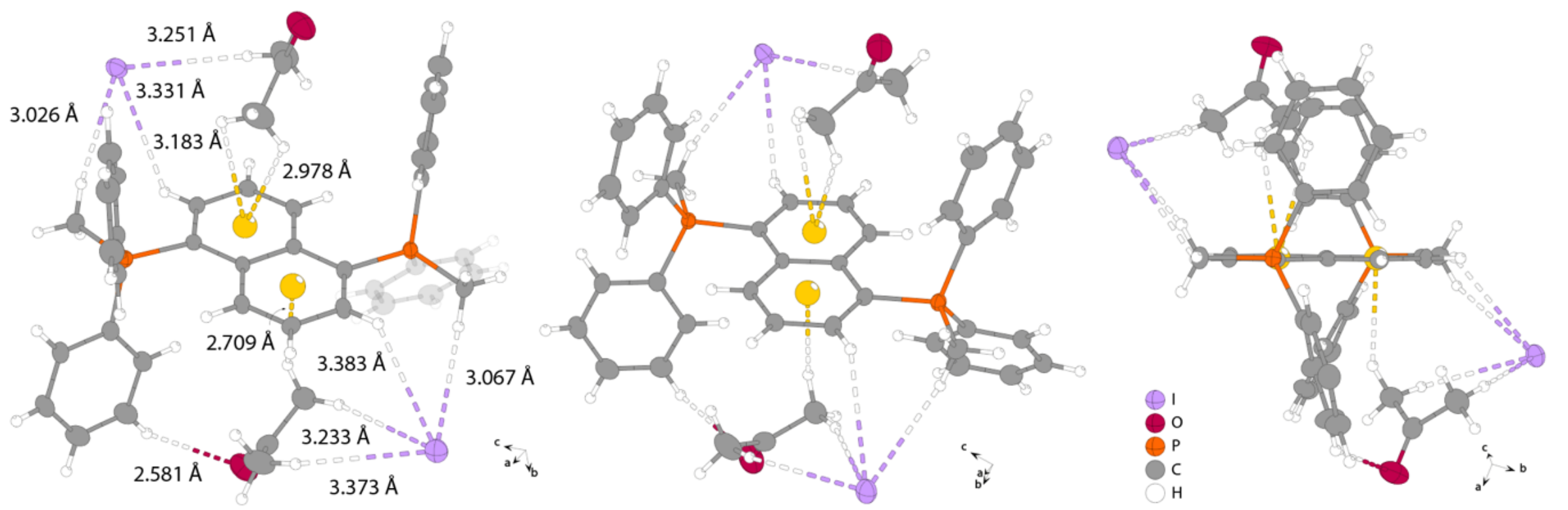

Figure S20. Crystal structure of **1·A^g^**.

Table S4. Bond lengths (d), energies (E) and topological characteristics of the electron density distribution at the (3; -1) critical points of the I---X bonds (X=C,H) of **1·A** and **1·A°** crystal structures.

	d, Å	ρ(**r**), ea_0^{-3}	ν(**r**), au	*g*(**r**), au	h_e(**r**), au	$\nabla^2\rho$(**r**), ea_0^{-5}	*E*, kcal/mol	ε	DI
**1·A**									
I1(2)---H1	3.950	0.0020	-0.0006	0.0010	0.0004	0.0057	**-0.19**	0.63	0.02
I1(2)---H2	3.042	0.0088	-0.0043	0.0051	0.0008	0.0241	**-1.35**	0.02	0.07
I1(2)---H3	3.526	0.0039	-0.0013	0.0020	0.0007	0.0105	**-0.41**	0.05	0.04
I1(2)---H4	3.283	0.0059	-0.0024	0.0032	0.0008	0.0157	**-0.75**	0.01	0.06
I1(2)---C1	4.022	0.0049	-0.0018	0.0024	0.0006	0.0118	**-0.56**	0.69	0.04
Total							**-3.26**		
**1·A°**									
I1---H1	3.315	0.0054	-0.0022	0.0030	0.0008	0.0154	**-0.69**	0.04	0.05
I1---H2	3.281	0.0057	-0.0024	0.0033	0.0009	0.0166	**-0.75**	0.03	0.05
I1---H3	3.051	0.0085	-0.0042	0.0051	0.0009	0.0243	**-1.32**	0.02	0.07
I1---H4	3.262	0.0059	-0.0025	0.0034	0.0009	0.0172	**-0.78**	0.05	0.05
I1---H5	3.874	0.0022	-0.0006	0.0010	0.0004	0.0058	**-0.19**	0.37	0.02
I1---C1	4.183	0.0039	-0.0013	0.0017	0.0004	0.0086	**-0.41**	0.16	0.03
I1---C2	4.943	0.0010	-0.0003	0.0004	0.0001	0.0023	**-0.09**	6.39	0.01
I2---H1	3.808	0.0023	-0.0006	0.0011	0.0005	0.0064	**-0.19**	0.07	0.03
I2---H2	3.621	0.0031	-0.0010	0.0016	0.0006	0.0089	**-0.31**	0.12	0.03
I2---C1	4.041	0.0048	-0.0016	0.0022	0.0006	0.0116	**-0.50**	1.90	0.04
Total							**-5.23**		

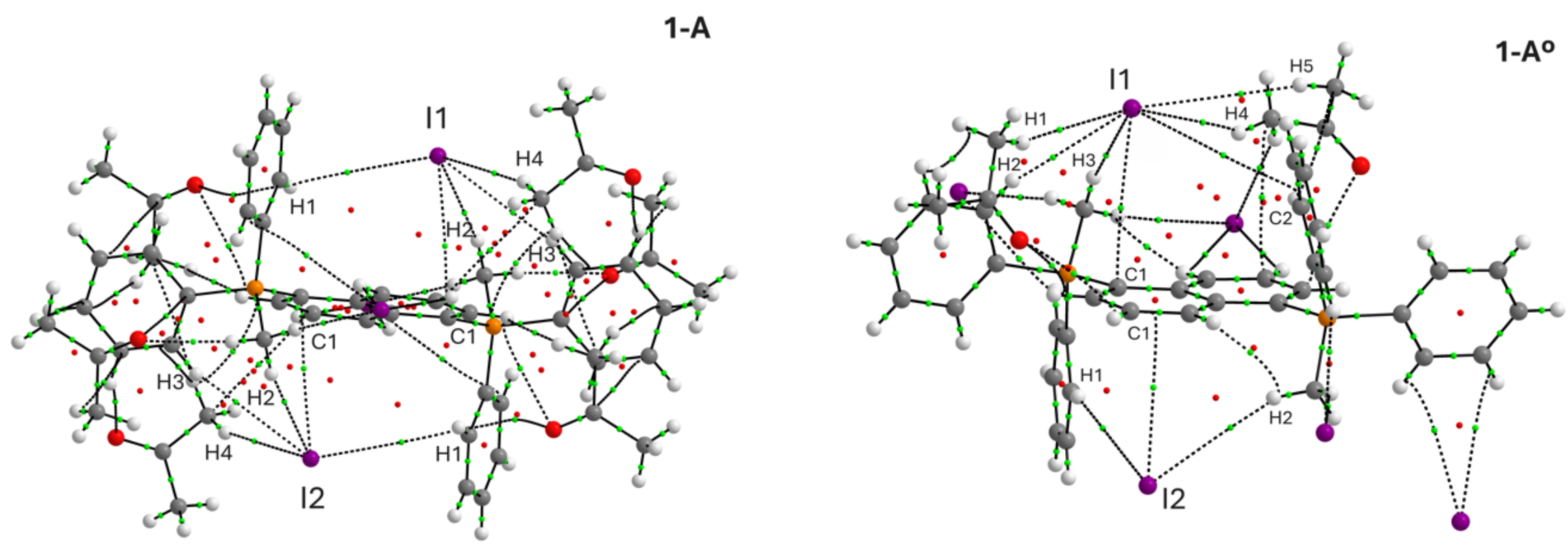

Figure S21. QTAIM analysis of **1·A** and **1·A°** crystal structures with marked bond critical points (3, -1) (green dots), ring critical points (3, +1) (red dots), covalent and non-covalent interactions (solid and dashed lines, respectively).

Table S5. Bond lengths (d), energies (E) and topological characteristics of the electron density distribution at the (3; -1) critical points of the I---X bonds (X=C, H) of **1·N** and **1·N°** crystal structures.

	d, Å	ρ(**r**), ea_0^{-3}	ν(**r**), au	*g*(**r**), au	h_e(**r**), au	$\nabla^2\rho$(**r**), ea_0^{-5}	*E*, kcal/mol	ε	DI
**1·N**									
I1(2)---H1	3.187	0.0070	-0.0031	0.0040	0.0009	0.0193	**-0.97**	0.01	0.06
I1(2)---H2	3.017	0.0093	-0.0046	0.0055	0.0009	0.0254	**-1.44**	0.03	0.07
I1(2)---H3	4.326	0.0012	-0.0003	0.0005	0.0002	0.0029	**-0.09**	0.87	0.01
I1(2)---C1	3.957	0.0056	-0.0021	0.0027	0.0006	0.0135	**-0.66**	1.00	0.04
Total							**-3.17**		
**1·N°**									
I1---H1	3.687	0.0028	-0.0008	0.0013	0.0005	0.0074	**-0.25**	0.04	0.03
I1---H2	3.164	0.0070	-0.0032	0.0041	0.0009	0.0203	**-1.00**	0.02	0.06
I1---H3	2.985	0.0096	-0.0048	0.0058	0.0010	0.0267	**-1.51**	0.02	0.07
I1---H4	3.152	0.0070	-0.0032	0.0040	0.0008	0.0192	**-1.00**	0.01	0.06
I1---C1	4.262	0.0034	-0.0011	0.0015	0.0004	0.0076	**-0.35**	0.16	0.03
I1---C2	5.167	0.0007	-0.0002	0.0003	0.0001	0.0016	**-0.06**	6.58	0.01
I2---H1	3.674	0.0031	-0.0009	0.0014	0.0005	0.0076	**-0.28**	0.02	0.04
I2---H2	4.287	0.0010	-0.0002	0.0004	0.0002	0.0026	**-0.06**	0.13	0.01
I2---H3	3.412	0.0045	-0.0017	0.0025	0.0007	0.0128	**-0.53**	0.07	0.04
I2---C1	4.127	0.0042	-0.0013	0.0019	0.0006	0.0098	**-0.41**	1.61	0.03
Total							**-5.46**		

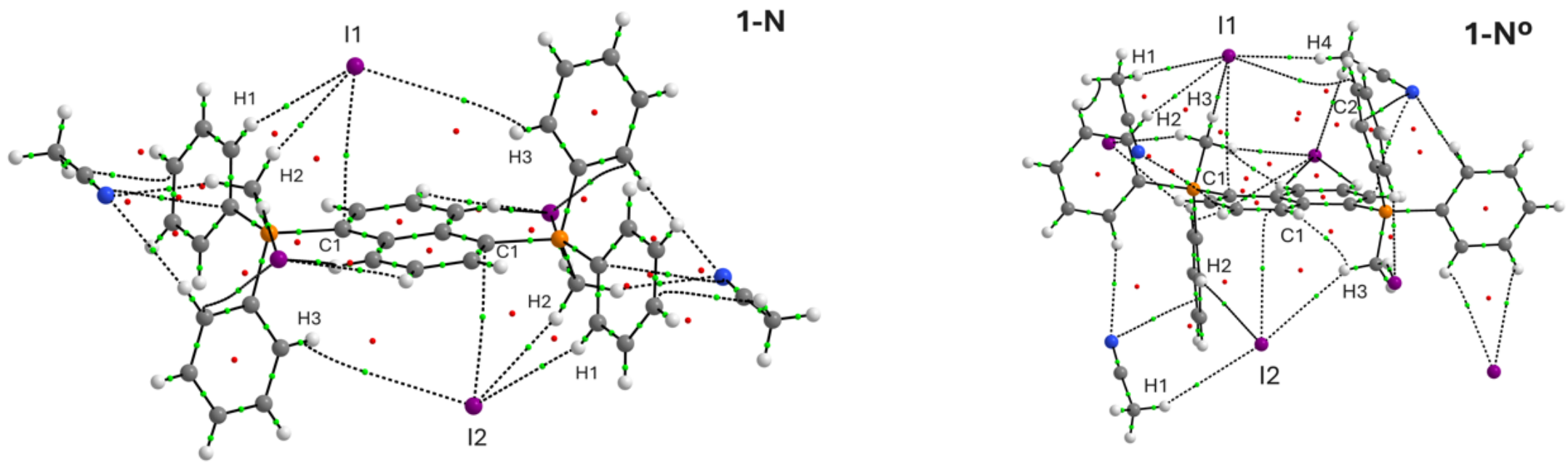

Figure S22. QTAIM analysis of **1·N** and **1·N°** crystal structures with marked bond critical points (3, -1) (green dots), ring critical points (3, +1) (red dots), covalent and non-covalent interactions (solid and dashed lines, respectively).

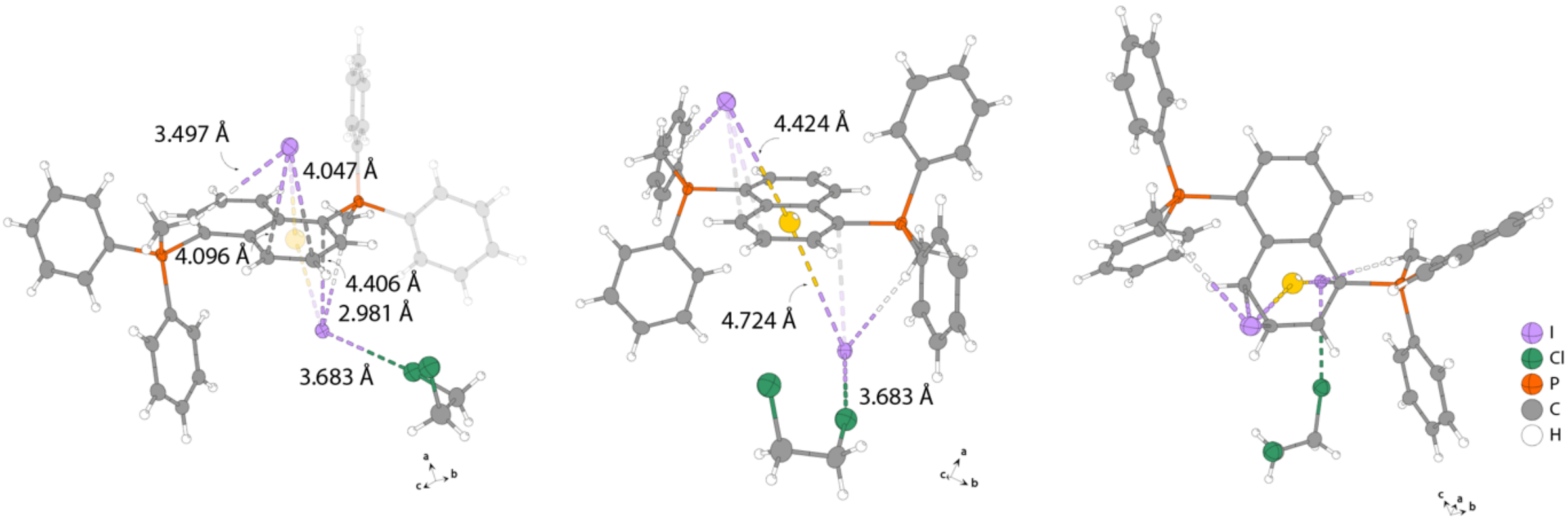

Figure S23. Crystal structure of **1·E**.

Table S6. Bond lengths (d), energies (E) and topological characteristics of the electron density distribution at the (3; -1) critical points of the I---X bonds (X=C,H,Cl) of **1·C** and **1·E** crystal structures.

	d, Å	ρ(**r**), ea_0^{-3}	ν(**r**), au	*g*(**r**), au	h_e(**r**), au	∇^2ρ(**r**), ea_0^{-5}	*E*, kcal/mol	ε	DI
					**1·C**				
I1(2)---H1	2.646	0.0179	-0.0105	0.0107	0.0002	0.0193	**-3.29**	<0.01	0.11
I1(2)---H2	3.135	0.0078	-0.0035	0.0044	0.0009	0.0210	**-1.10**	0.04	0.06
I1(2)---H3	3.045	0.0093	-0.0047	0.0058	0.0011	0.0276	**-1.47**	0.07	0.06
I1(2)---H4	3.329	0.0057	-0.0026	0.0037	0.0011	0.0191	**-0.82**	0.48	0.03
I1(2)---C1	4.101	0.0040	-0.0013	0.0018	0.0005	0.0093	**-0.41**	0.14	0.04
I1(2)---Cl1	3.683	0.0082	-0.0039	0.0049	0.0010	0.0239	**-1.22**	0.01	0.14
Total							**-8.31**		
					**1·E**				
I1---H1	3.687	0.0030	-0.0009	0.0014	0.0005	0.0073	**-0.28**	0.02	0.04
I1---H2	4.115	0.0013	-0.0003	0.0006	0.0003	0.0035	**-0.09**	0.11	0.02
I1---H3	3.497	0.0038	-0.0014	0.0020	0.0006	0.0108	**-0.44**	0.08	0.04
I1---C1	4.047	0.0046	-0.0015	0.0022	0.0007	0.0112	**-0.47**	1.64	0.04
I2---H1	3.338	0.0050	-0.0021	0.0029	0.0008	0.0147	**-0.66**	0.08	0.04
I2---H2	2.981	0.0095	-0.0048	0.0057	0.0009	0.0266	**-1.51**	0.01	0.07
I2---H3	3.337	0.0060	-0.0026	0.0036	0.0010	0.0183	**-0.82**	0.30	0.04
I2---H4	4.575	0.0007	-0.0002	0.0003	0.0001	0.0019	**-0.06**	0.31	0.01
I2---H5	3.239	0.0067	-0.0031	0.0041	0.0011	0.0203	**-0.97**	0.23	0.05
I2---Cl1	3.683	0.0084	-0.0040	0.0050	0.0010	0.0242	**-1.26**	0.01	0.15
I2---C1	4.405	0.0028	-0.0009	0.0012	0.0003	0.0061	**-0.28**	0.23	0.02
I3---H6	3.344	0.0051	-0.0021	0.0028	0.0007	0.0142	**-0.66**	0.13	0.04
I3---H7	3.230	0.0066	-0.0031	0.0041	0.0011	0.0208	**-0.97**	0.16	0.04
I3---H8	3.177	0.0071	-0.0034	0.0045	0.0011	0.0225	**-1.07**	0.09	0.05
I3---Cl2	3.683	0.0083	-0.0041	0.0052	0.0010	0.0248	**-1.29**	<0.01	0.14
I3---C2	4.557	0.0019	-0.0005	0.0008	0.0003	0.0045	**-0.16**	0.20	0.02
Total							**-10.99**		

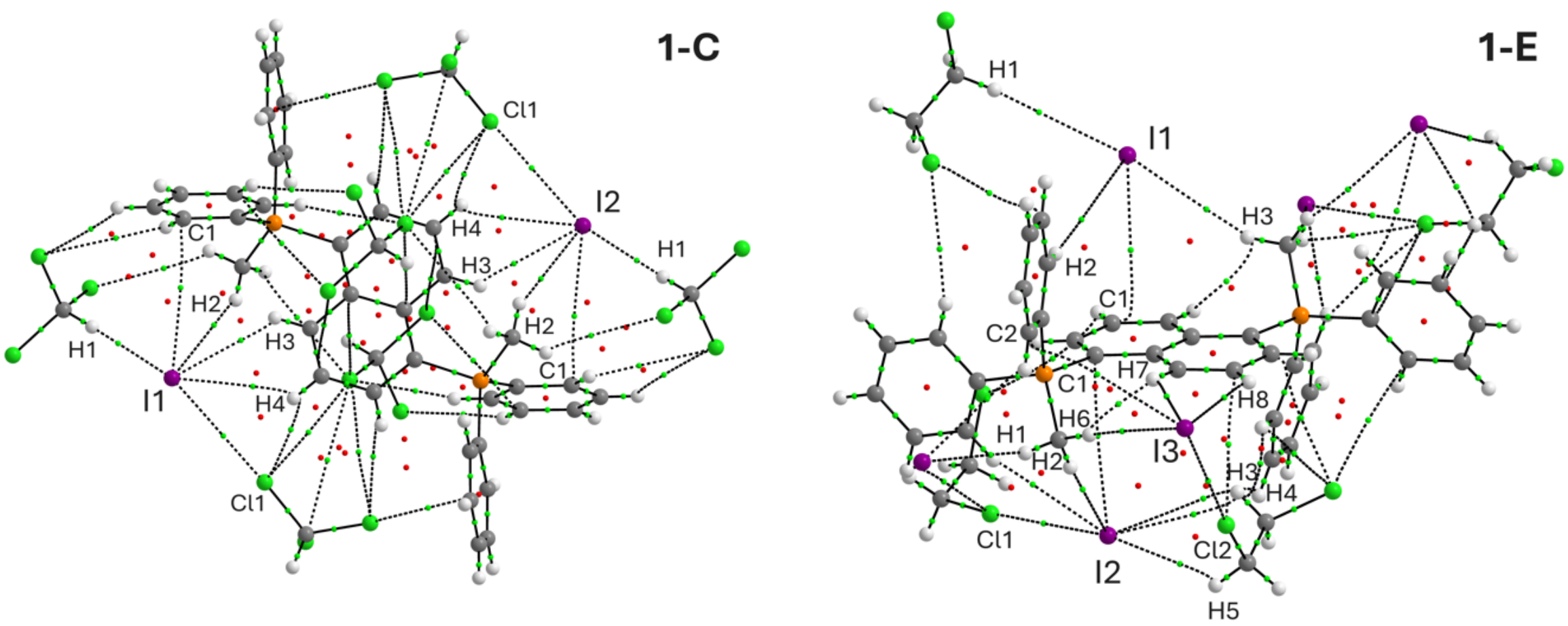

Figure S24. QTAIM analysis of **1·C** and **1·E** crystal structures with marked bond critical points (3, -1) (green dots), ring critical points (3, +1) (red dots), covalent and non-covalent interactions (solid and dashed lines, respectively).

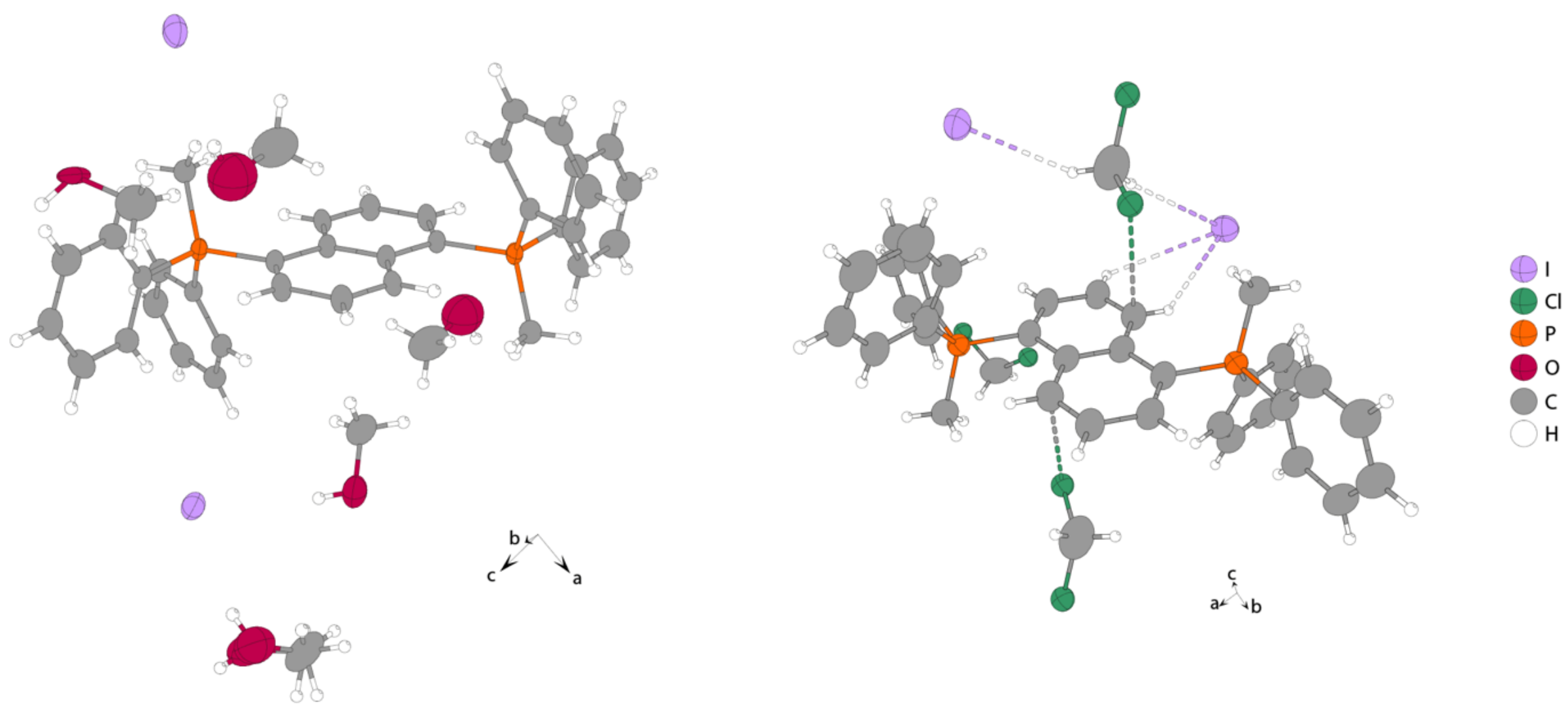

Figure S25. Crystal structures of **1·M** (left) and **1·D** (right).

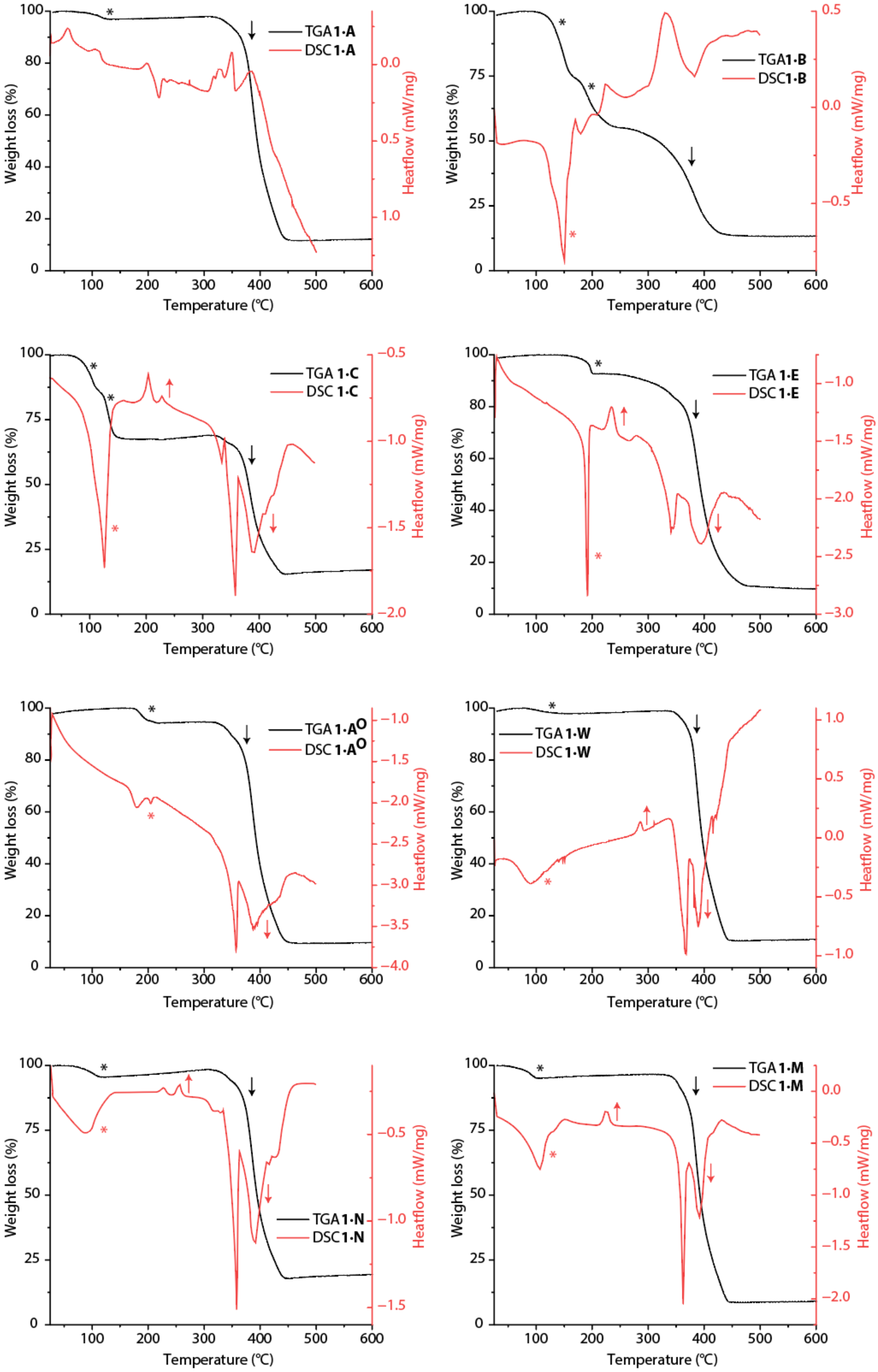

Figure S26. TGA and DSC curves of the solvated forms of **1**.

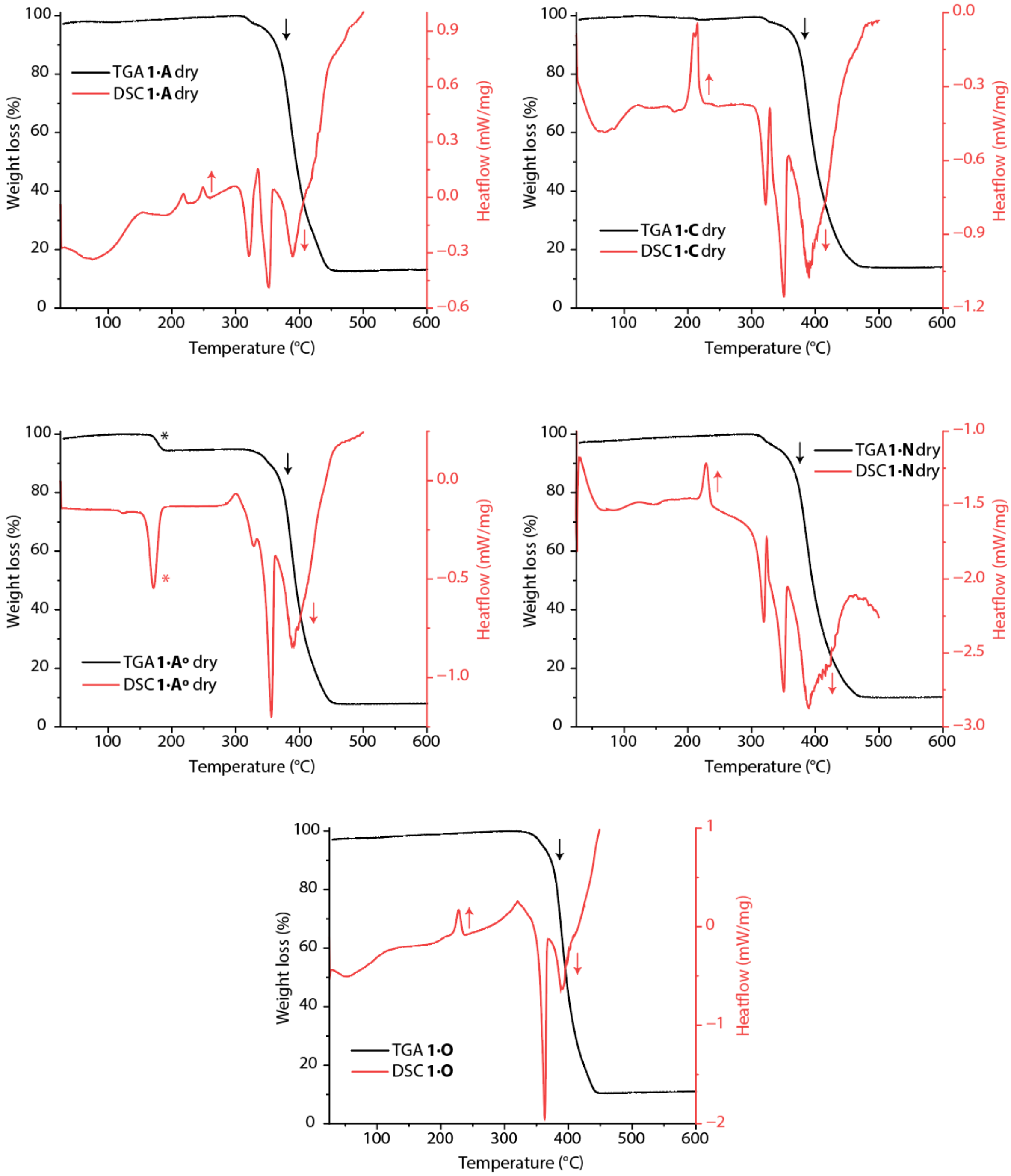

Figure S27. TGA and DSC curves of the dried forms of **1**.

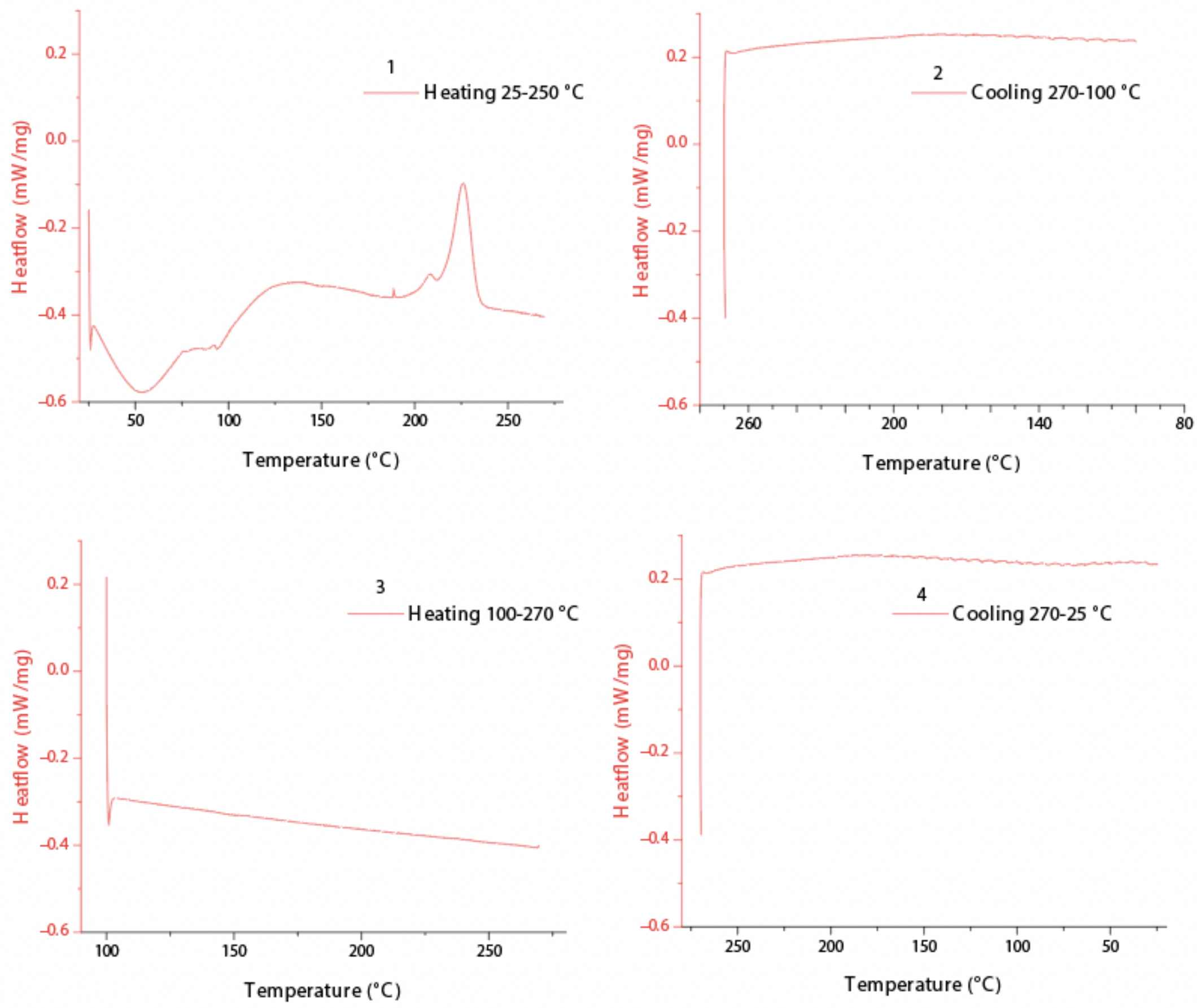

Figure S28. Cyclic DSC measurements of **1**·**N** showing formation of the amorphous phase upon solvent removal in the temperature range 0–100 ºC followed by an exothermic crystallization peak at 225 ºC. Cycles 2–4 demonstrate no phase transition.

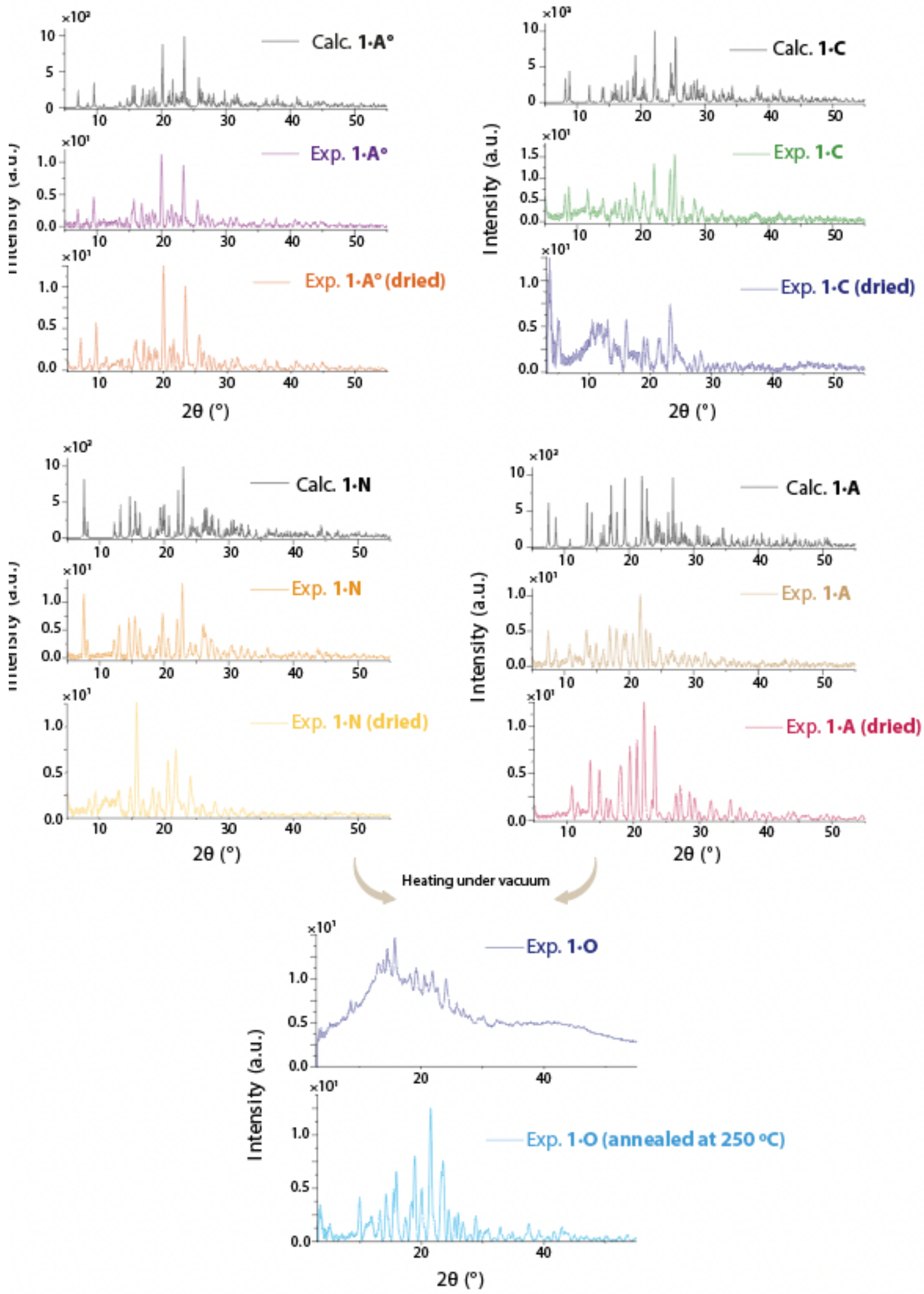

Figure S29. Simulated and experimental PXRD patterns for microcrystalline **1·C**, **1·N**, **1·A°**, and **1·A** measured from fresh phases and gently dried forms (vacuum). Thermal heating of **1·A** and **1·N** leads to amorphous phase that, upon annealing, converts into an unknown crystalline phase.

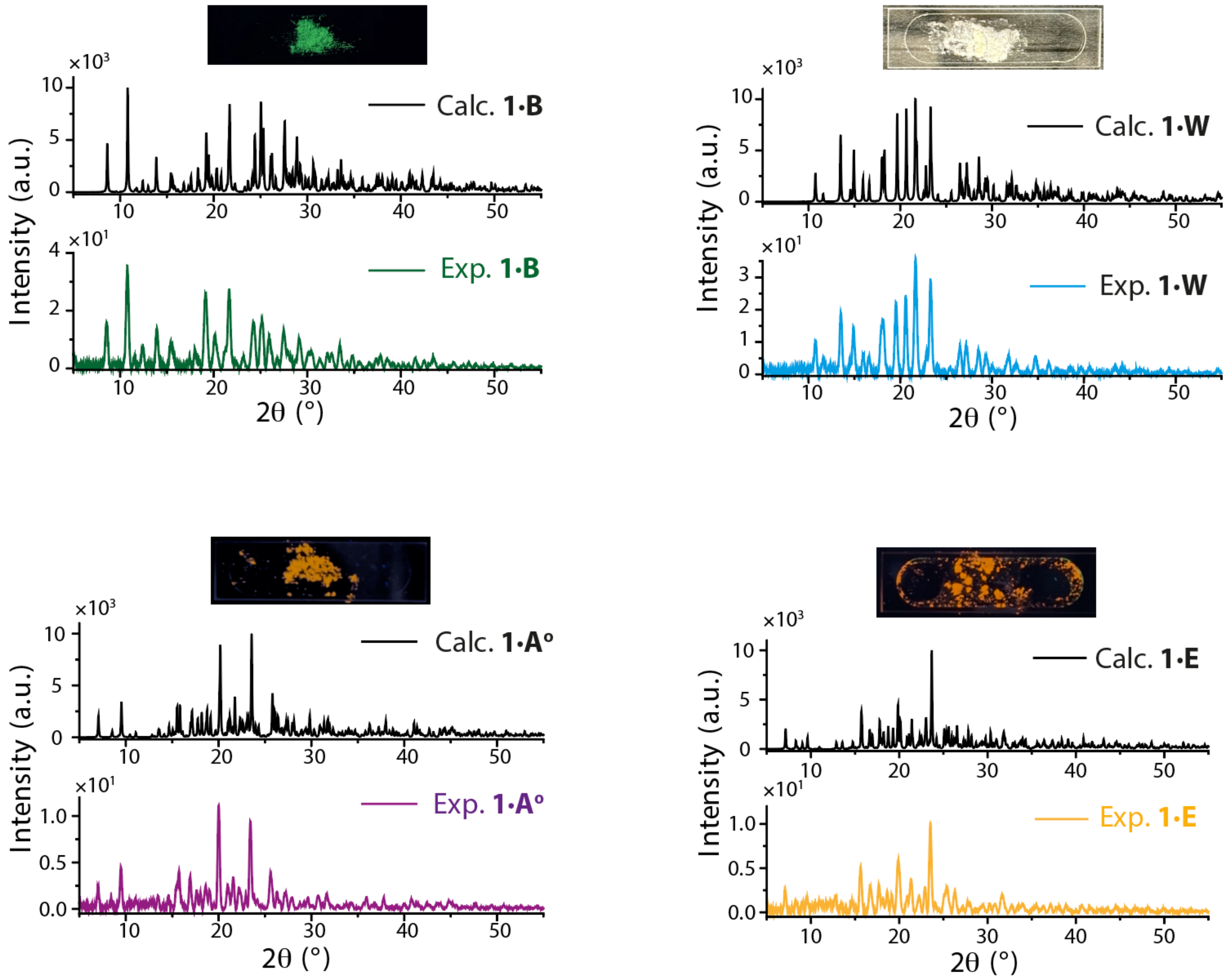

Figure S30. Simulated and experimental PXRD patterns for **1**·**B**, **1**·**W**, **1**·**A°**, and **1**·**E** (inset shows crystalline material under 385 nm irradiation).

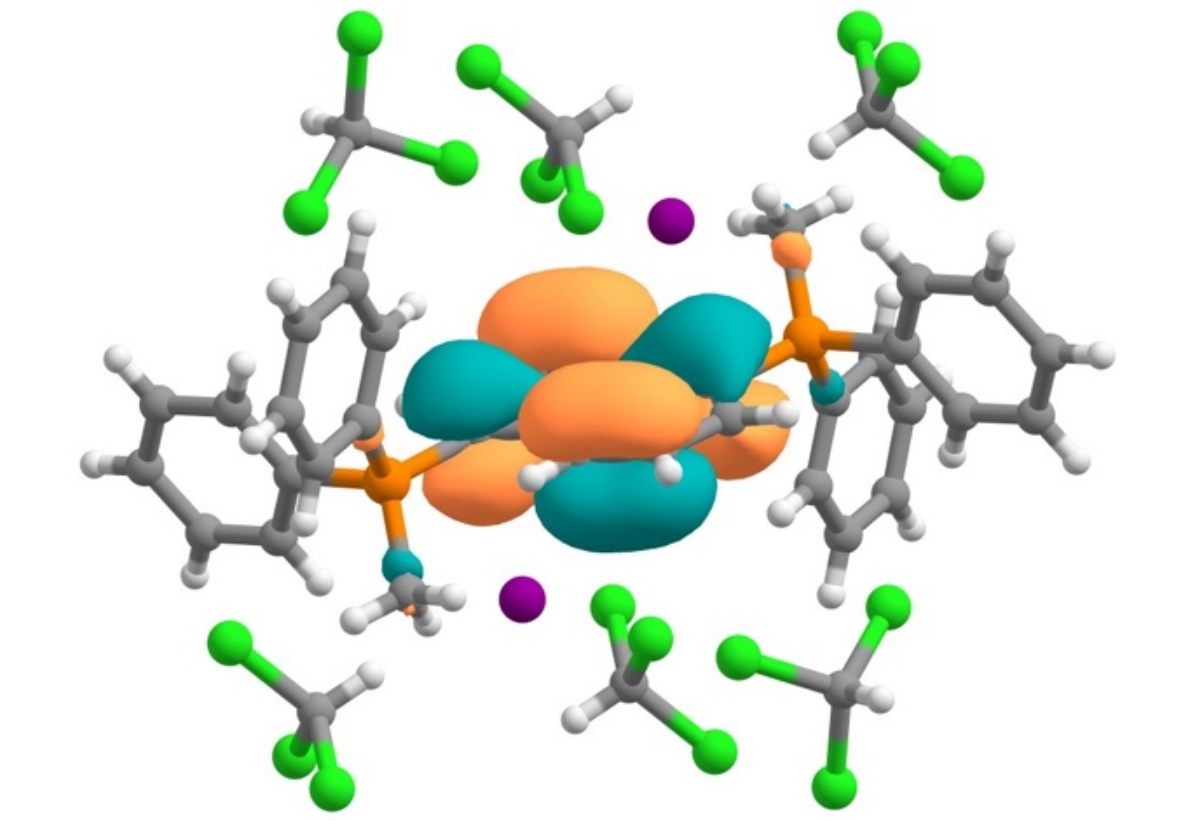	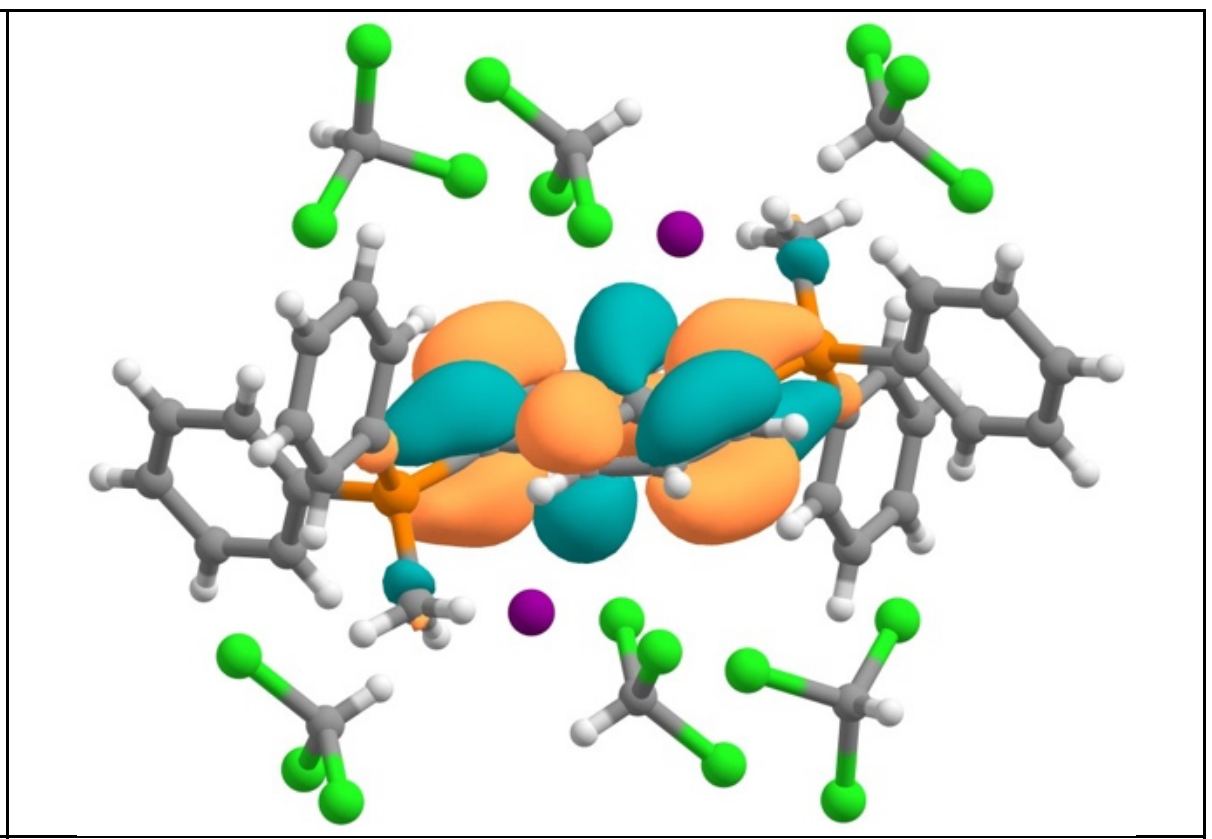
HOMO-6	LUMO

Figure S31. Molecular orbitals corresponding to the T_1 excited state of the **1·C** crystal structure fragment calculated by the wB97XD/DGDZVP method. The calculated energy of the T_1 state (465 nm) is well in agreement with the experimental phosphorescence spectrum (491 nm). The HOMO-6→LUMO configuration has a contribution of 75% in the T_1 state, while other configurations have a weight less than 5%.

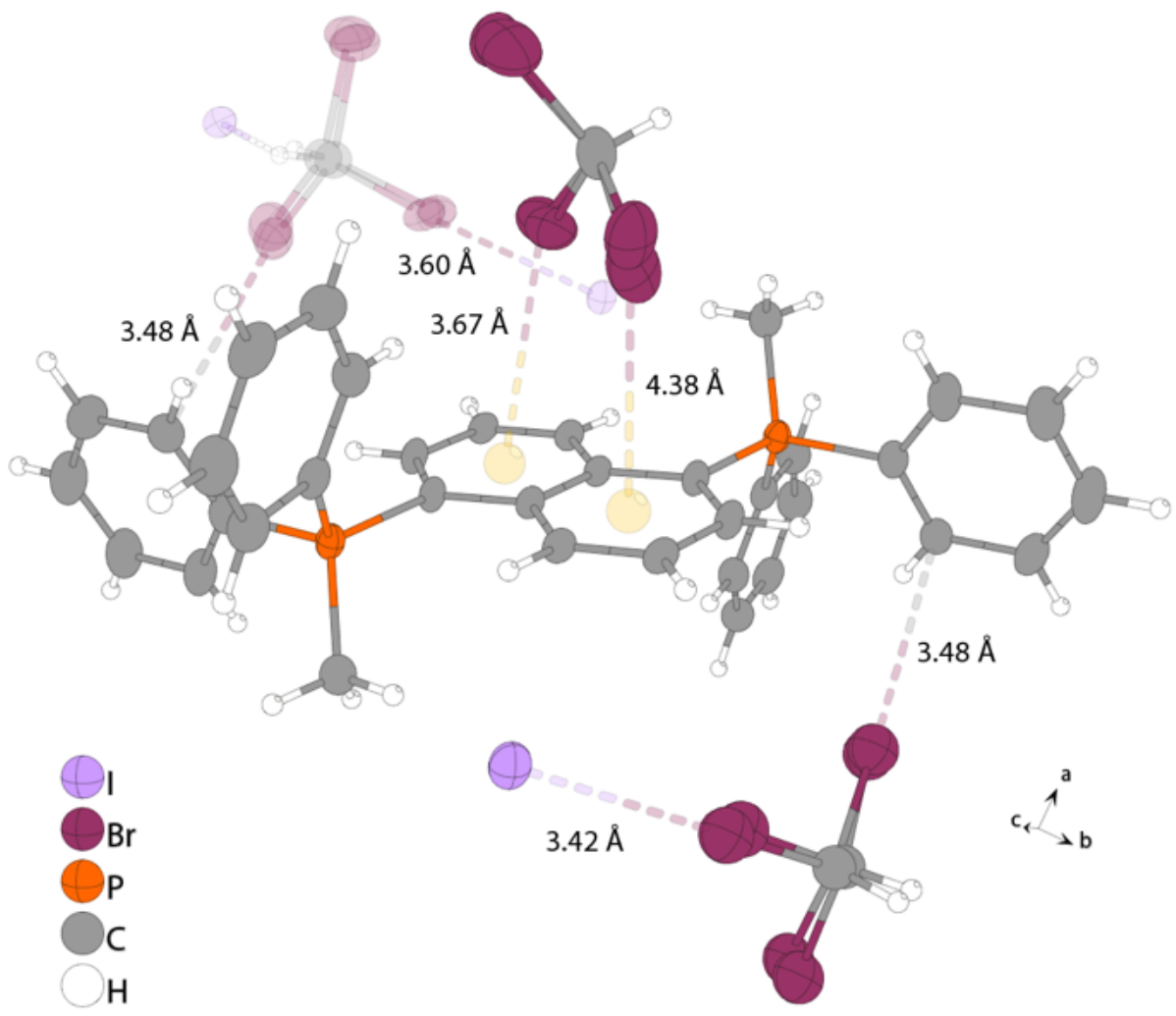

Figure S32. Crystal structure of **1·B** with in-plane I–cation arrangement and solvation of cationic core with molecules of bromoform.

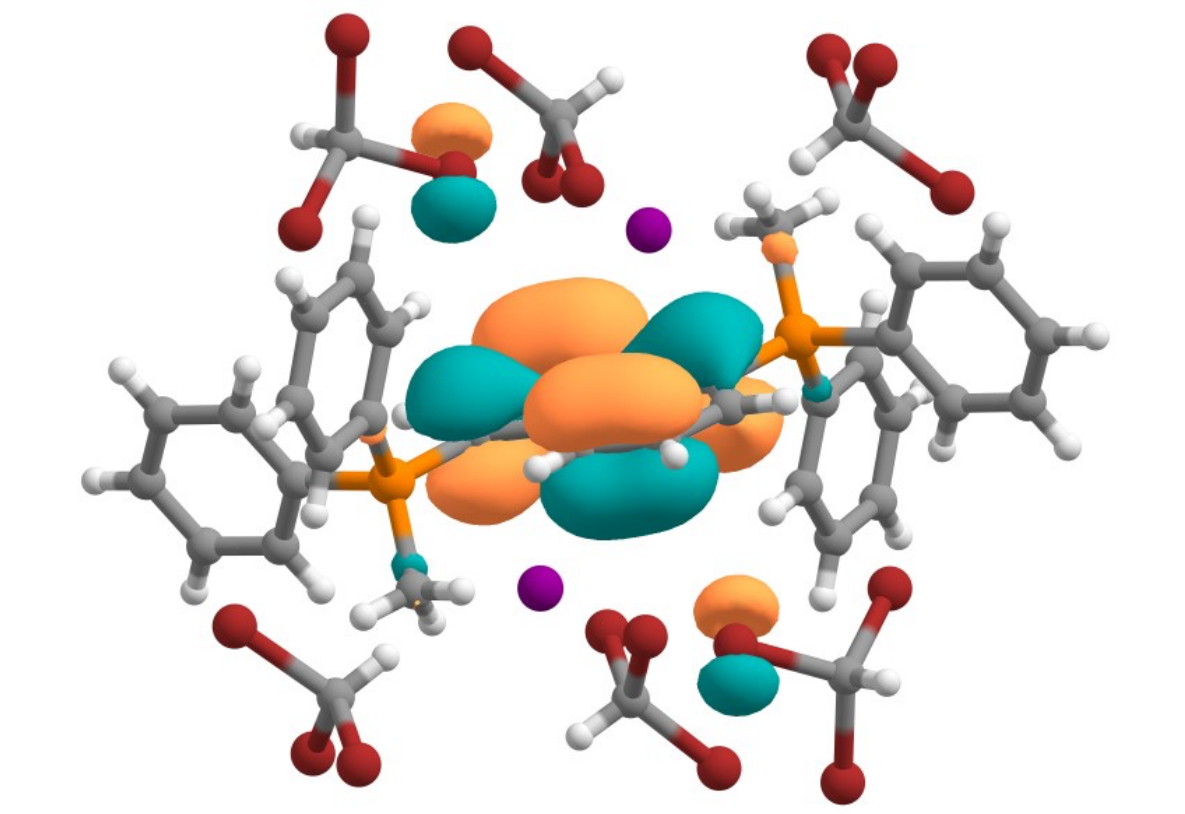	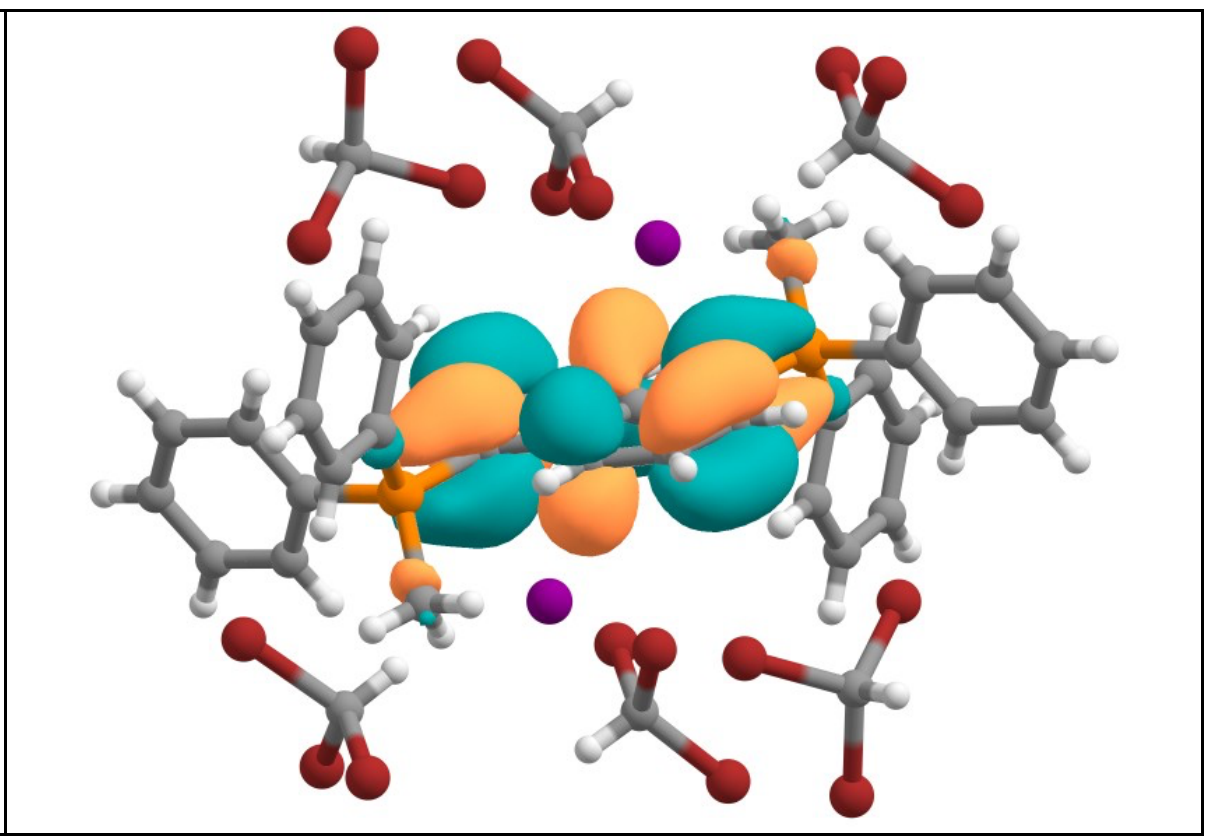
HOMO-6	LUMO

Figure S33. Molecular orbitals corresponding to the T_1 excited state of **1·B** crystal structure fragment calculated by wB97XD/DGDZVP method. The calculated energy of the T_1 state (473 nm) is well in agreement with the experimental phosphorescence spectrum (500 nm). The HOMO-6→LUMO configuration has a contribution of 74% in the T_1 state, while other configurations have a weight of less than 4%.

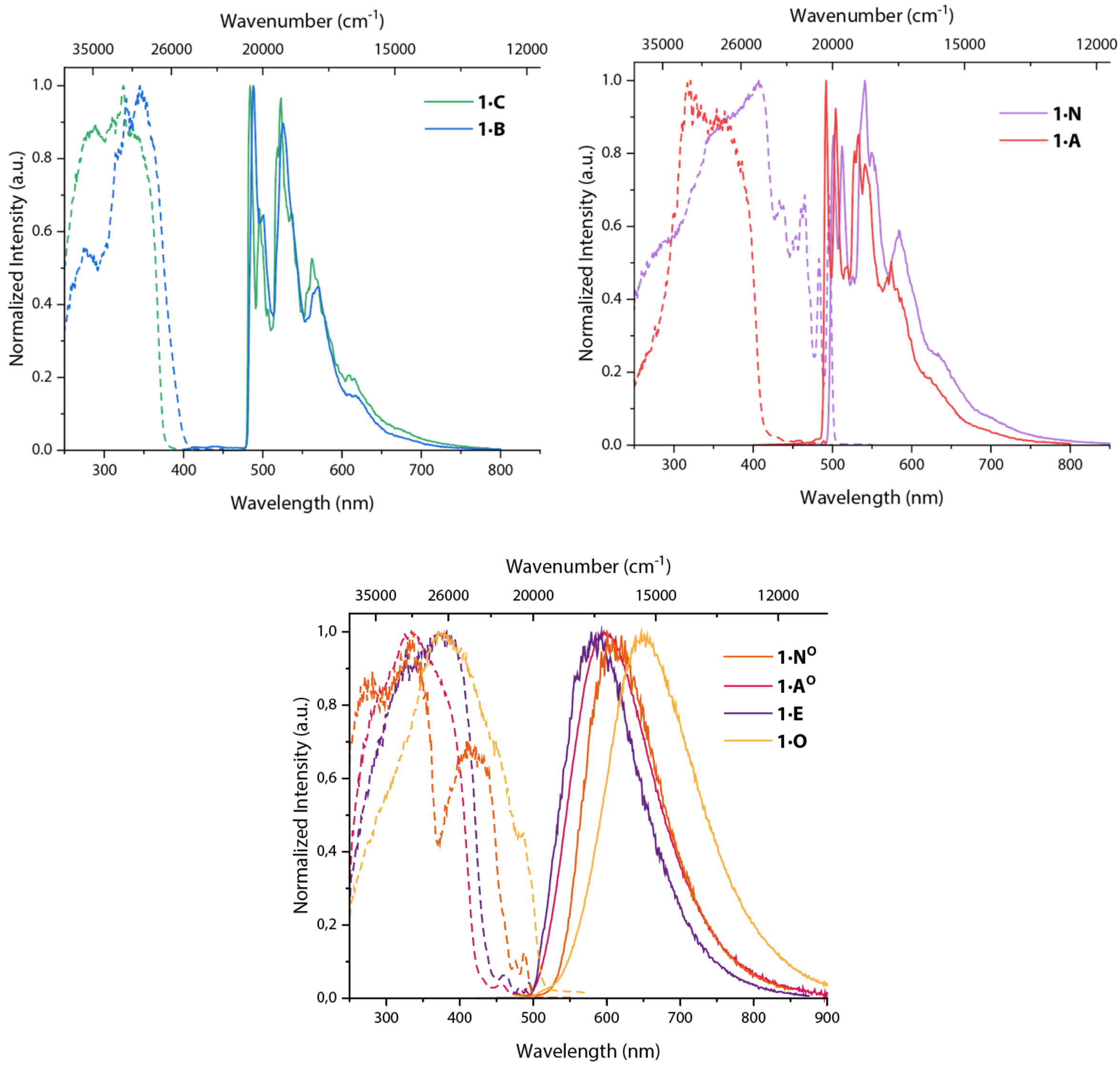

Figure S34. Excitation and emission spectra of **1·C**, **1·B**, **1·N**, **1·A** and **1·N^o**, **1·A^o**, **1·E, 1·O** at 77 K.

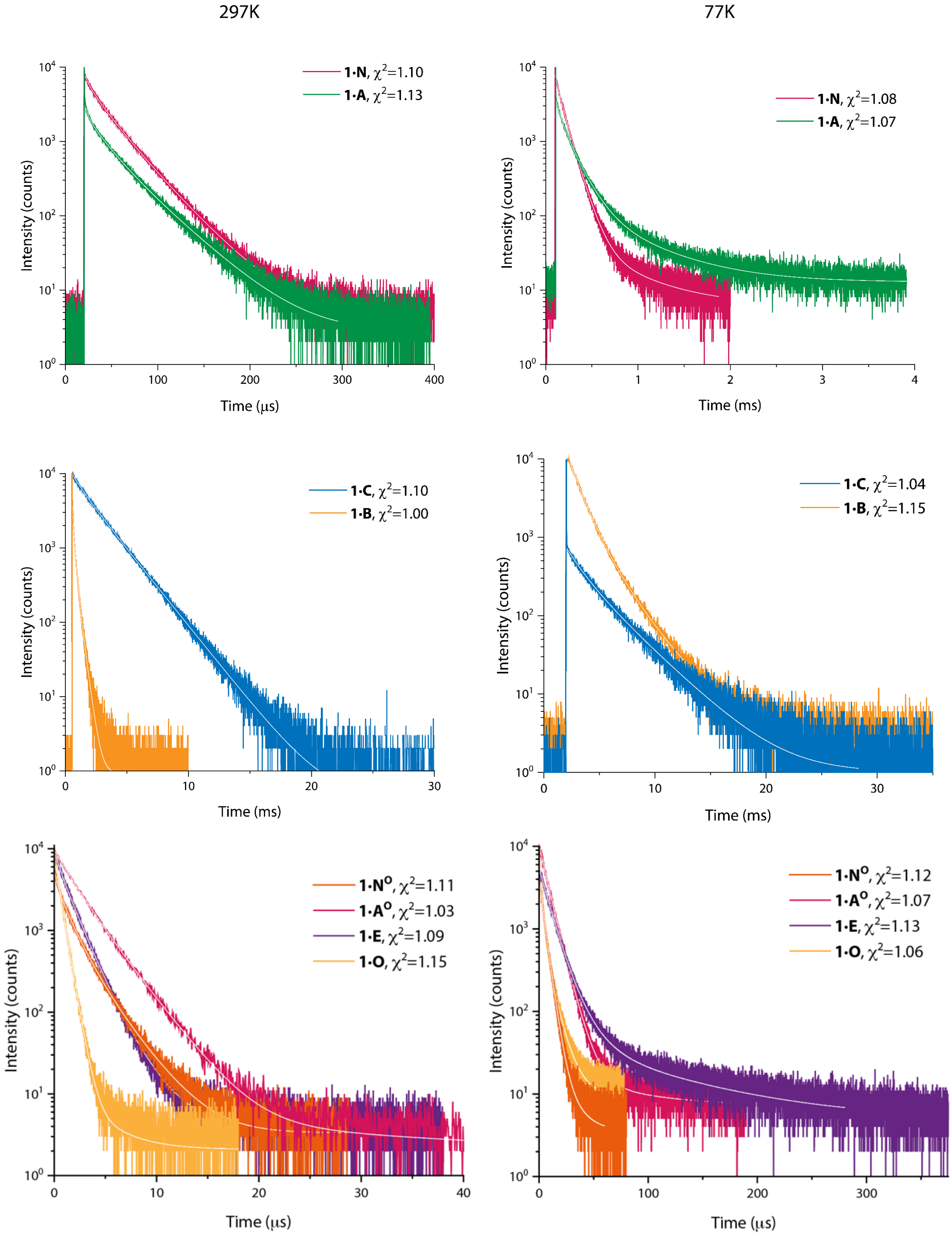

Figure S35. Decay lifetimes of the excited state and their fit of **1·C**, **1·B**, **1·N**, **1·A** and **1·N**^{o}, **1·A**^{o}, **1·E**, **1·O** at 298 (left column) and 77 K (right column).

Table S7. Summary of the photophysical properties of different solvatomorphs of **1** in the solid state at room temperature.

Compound	λ_{exc} [nm]	λ_{em} [nm]	τ (A_i) [μs]	τ_{av} [μs][a]	Φ_{em}	k_r [$10^5 s^{-1}$] [b]	k_{nr} [$10^5 s^{-1}$] [b]
**1·N**	370	521, 551, 584	5.40 (20.93 %)<br>20.56 (40.78 %)<br>34.80 (38.29 %)	22.8	-	-	-
**1·C**	325	491, 500, 528, 569	776.79 (16.59 %)<br>2100.70 (83.41 %)	1881.1	0.68	0.0036	0.0017
**1·B**	364	500, 529, 568	37.39 (47.67 %)<br>140.46 (40.38 %)<br>342.14 (11.95%)	115.4	0.11	0.0096	0.0772
**1·A°**	366	620	2.12 (50.21 %)<br>3.16 (49.79 %)	2.6	0.21	0.8076	3.0385
**1·W**	358	415, 435	2.06×10^{-3} (66.27 %)<br>8.50×10^{-3} (25.69 %)<br>59.98×10^{-3} (8.04 %)	0.009	-	-	-
**1·A**	366	511, 544, 577	3.31 (22.29 %)<br>18.02 (38.95 %)<br>39.29 (38.76 %)	23.0	-	-	-
**1·A^g**	410	482, 492, 520, 560, 610	5.60 (18.18 %)<br>33.38 (15.15 %)<br>117.46 (63.64 %)<br>825.73 (3.03 %)	105.9	-	-	-
**1·N°**	343, 428	618	0.35 (29.34 %)<br>1.23 (52.45 %)<br>2.66 (18.21 %)	1.2	-	-	-
**1·E**	380	634	0.38 (19.33 %)<br>1.32 (73.46 %)<br>2.43 (7.20 %)	1.2	-	-	-
**1·D***	365	514, 544, 670	3.47 (37.26 %)<br>7.67 (55.52 %)<br>14.59 (7.23 %)	6.6	-	-	-
**1·M**	358*	417*	0.90×10^{-3} (73.34 %)*<br>3.38×10^{-3} (21.98 %)*<br>12.66×10^{-3} (2.82 %)*<br>57.26×10^{-3} (1.85 %)*	0.03*	-	-	-
**1·O**	450	650	0.37 (23.84 %)<br>0.59 (75.98 %)<br>3.05 (0.18 %)	0.5	0.09	1.6544	1.6727

[a] Amplitude-weighted average lifetime, calculated from $\tau_{av}=\Sigma A_i\tau_i$, A_i=weight of the i-th exponent.
[b] Calculated from amplitude average lifetimes $k_r = \Phi_{em} /\tau_{av}$; $k_{nr} = (1- \Phi_{em})/\tau_{av}$. *Cannot be accurately determined due to low emission intensity.

Table S8. Summary of the photophysical properties of solvatomorphs **1** in the solid state at 77 K.

Compound	λ_{exc} [nm]	λ_{em} [nm]	τ (A_i) [μs]	τ_{av} [μs][a]
**1·N**	407	501, 512, 541, 550, 584, 633	29.46 (20.39 %)<br>102.47 (77.72 %)<br>217.95 (1.89 %)	89.8
**1·C**	359	485, 497, 523, 538, 562	1285.04 (42.95 %)<br>3211.76 (57.05 %)	2384.2
**1·B**	346	488, 501, 526, 570, 615	991.32 (76.53 %)<br>2144.78 (22.67 %)<br>4422.71 (0.80 %)	1280.3
**1·A^o**	327	584	6.80 (62.28 %)<br>9.20 (37.55 %)<br>51.78 (0.16 %)	7.8
**1·W**	366	485, 498, 519, 524, 538, 564, 610	183.47 (9.68 %)<br>582.13 (61.71 %)<br>1270.20 (26.92 %)<br>3566.19 (1.69 %)	779.2
**1·A**	364	492, 504, 533, 574, 624	52.58 (33.43 %)<br>152.25 (62.24 %)<br>609.21 (4.33 %)	138.7
**1·E**	375	585	8.72 (87.95 %)<br>16.97 (11.15 %)<br>89.17 (0.90 %)	10.4
**1·D***	369	494, 507, 533, 546, 570	9.52 (45.67 %)<br>22.25 (51.90 %)<br>59.89 (2.43 %)	17.4
**1·M**	388	487, 501, 525, 568	0.20 (49.76 %)<br>0.46 (36.58 %)<br>0.89 (13.27 %)<br>3.16 (0.40 %)	0.4
**1·O**	450	650	1.29 (61.00 %)<br>3.38 (32.57 %)<br>8.91 (6.43 %)	2.5

[a] Amplitude-weighted average lifetime, calculated from $\tau_{av}=\Sigma A_i\tau_i$, A_i=weight of the i-th exponent.

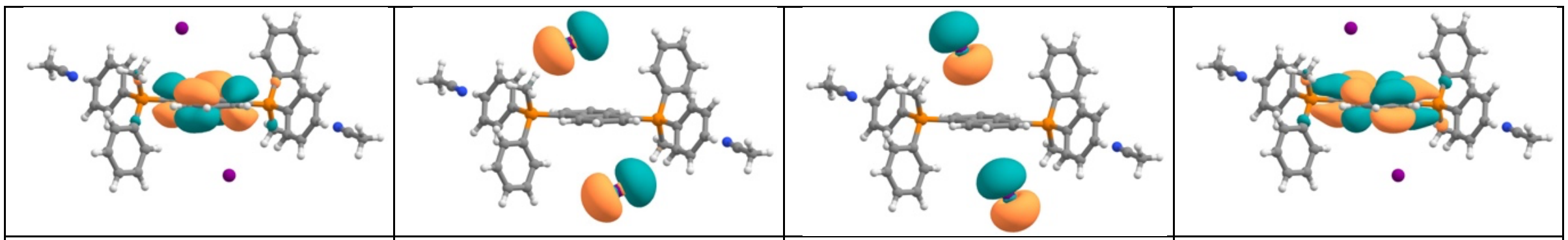

HOMO-6	HOMO-4	HOMO	LUMO
HOMO-6→ LUMO (21%) LE<br>HOMO-4→ LUMO (16%) CT<br>HOMO→ LUMO (54%) CT			

Figure S36. Molecular orbitals corresponding to the T_1 excited state of **1·N** crystal structure fragment with out-of-plane orientation of iodide counterions calculated by wB97XD/DGDZVP method. The calculated energy of the T_1 state (526 nm) is well in agreement with the experimental phosphorescence spectrum (521 nm). The contribution of the CT configuration in the T_1 state is 70%, dominating the LE contribution (21%).

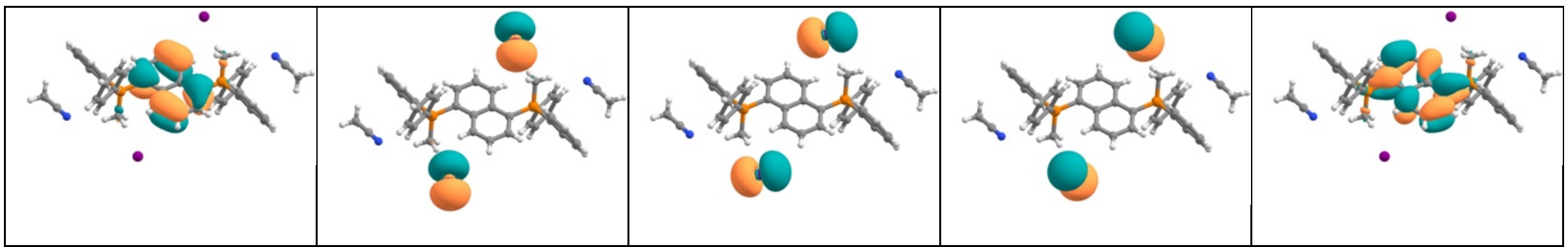

HOMO-6	HOMO-5	HOMO-2	HOMO	LUMO
HOMO-6→ LUMO (12%) LE<br>HOMO-5→ LUMO (24%) CT<br>HOMO-2→ LUMO (30%) CT<br>HOMO→ LUMO (29%) CT				

Figure S37. Molecular orbitals corresponding to the T_1 excited state of **1·N** crystal structure fragment with in-plane orientation of iodide counterions calculated by wB97XD/DGDZVP method. The calculated energy of the T_1 state (544 nm) is well in agreement with the experimental phosphorescence spectrum (521 nm). The contribution of the CT configuration to the T_1 state is 83%, dominating the LE contribution (12%).

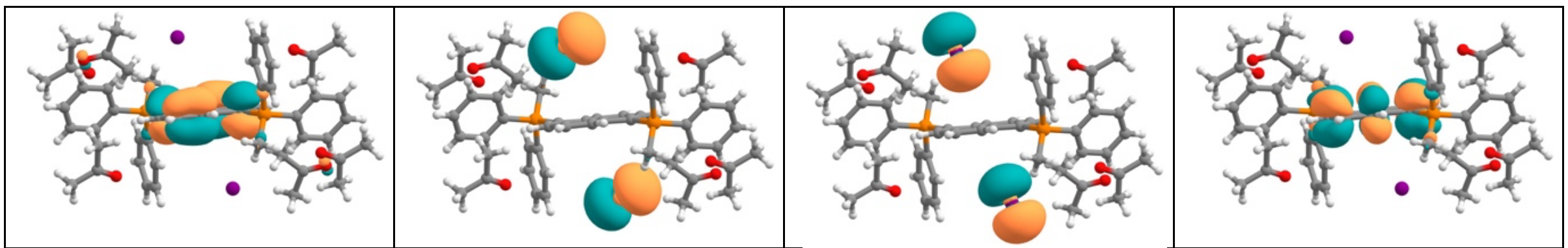

HOMO-6	HOMO-5	HOMO	LUMO
HOMO-6→ LUMO (24%) LE<br>HOMO-5→ LUMO (13%) CT<br>HOMO→ LUMO (57%) CT			

Figure S38. Molecular orbitals corresponding to the T_1 excited state of **1·A** crystal structure fragment with out-of-plane orientation of iodide counterions calculated by wB97XD/DGDZVP method. The calculated energy of the T_1 state (490 nm) is in good agreement with the experimental phosphorescence spectrum (511 nm). The contribution of the CT configuration to the T_1 state is 70%, dominating the LE contribution (24%).

<table>
<tr><td>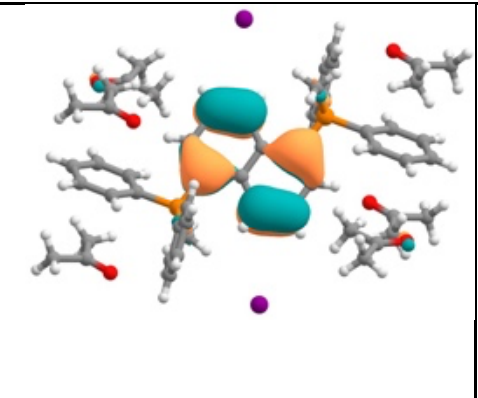</td><td>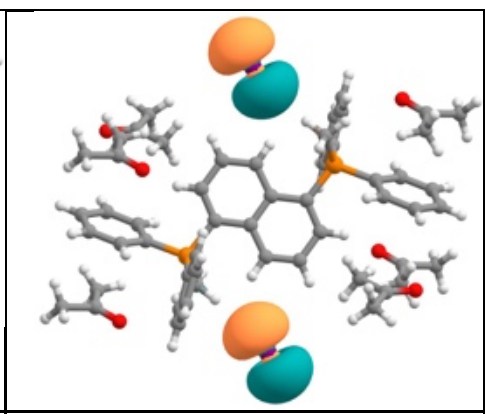</td><td>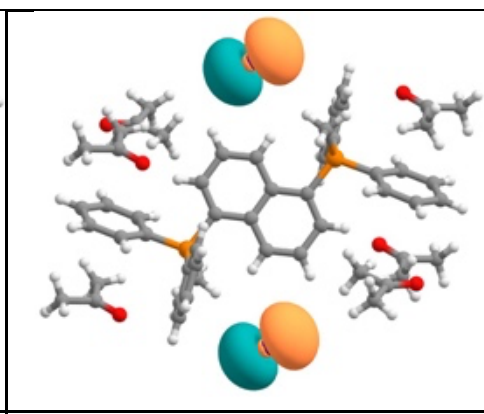</td><td>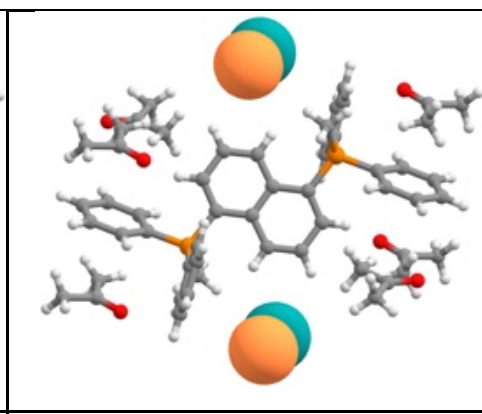</td><td>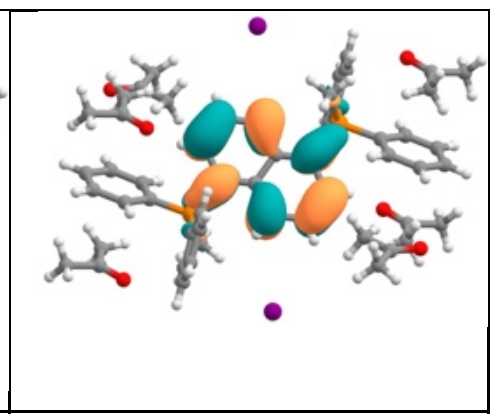</td></tr>
<tr><td>HOMO-6</td><td>HOMO-5</td><td>HOMO-3</td><td>HOMO</td><td>LUMO</td></tr>
<tr><td colspan="5">HOMO-6→ LUMO (14%) LE
HOMO-5→ LUMO (21%) CT
HOMO-3→ LUMO (20%) CT
HOMO→ LUMO (40%) CT</td></tr>
</table>

Figure S39. Molecular orbitals corresponding to the T_1 excited state of **1·A** crystal structure fragment with in-plane orientation of iodide counterions calculated by wB97XD/DGDZVP method. The calculated energy of the T_1 state (513 nm) is well in agreement with the experimental phosphorescence spectrum (511 nm). The contribution of the CT configuration in the T_1 state is 81%, dominating the LE contribution (14%).

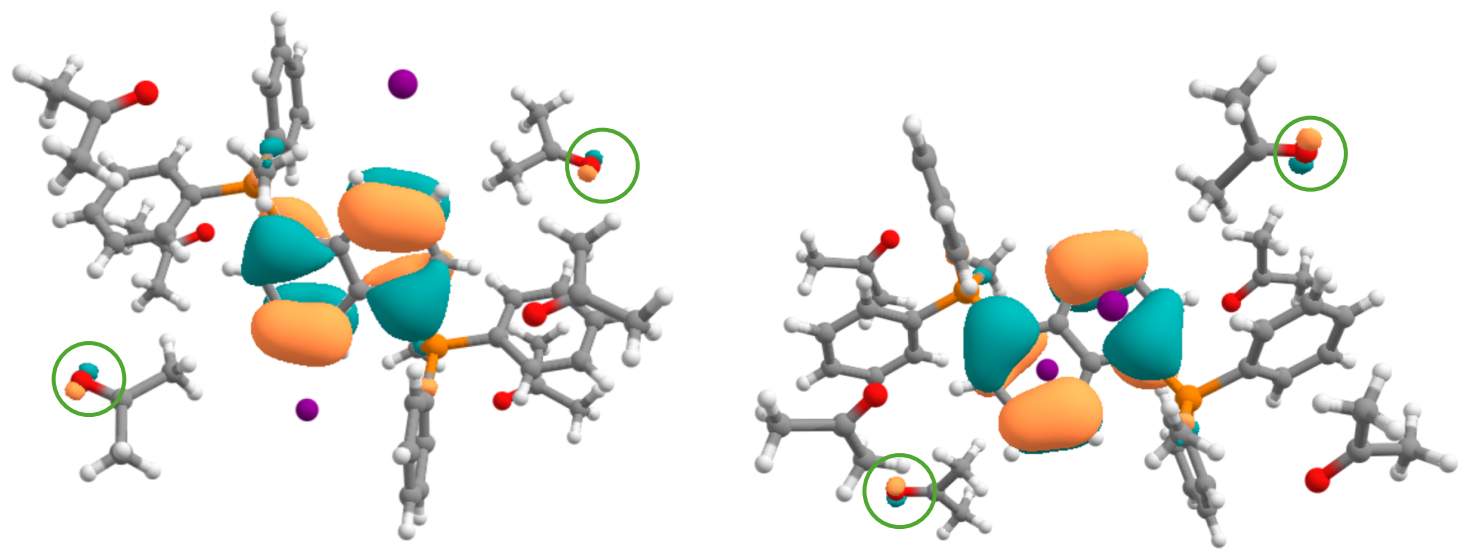

Figure S40. Shape of HOMO-6 orbital for **1·A** (in-plane orientation of I- (left) and out-of-plane orientation of I- (right)). Contribution from the lone pair of acetone solvent molecules marked by green circles.

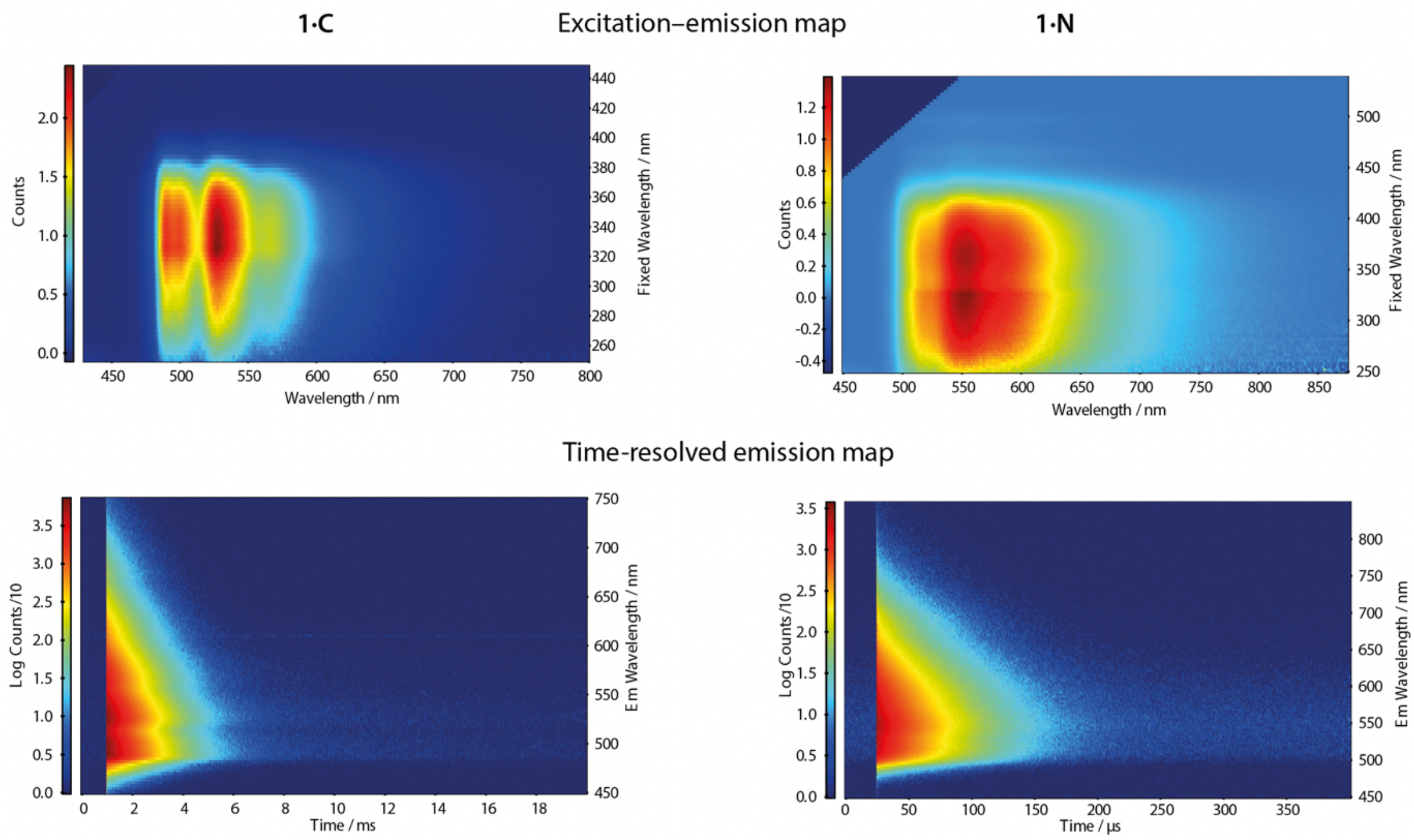

Figure S41. Excitation-emission mapping (up) and time-resolved emission spectra (bottom) of the **1·C** and **1·N**.

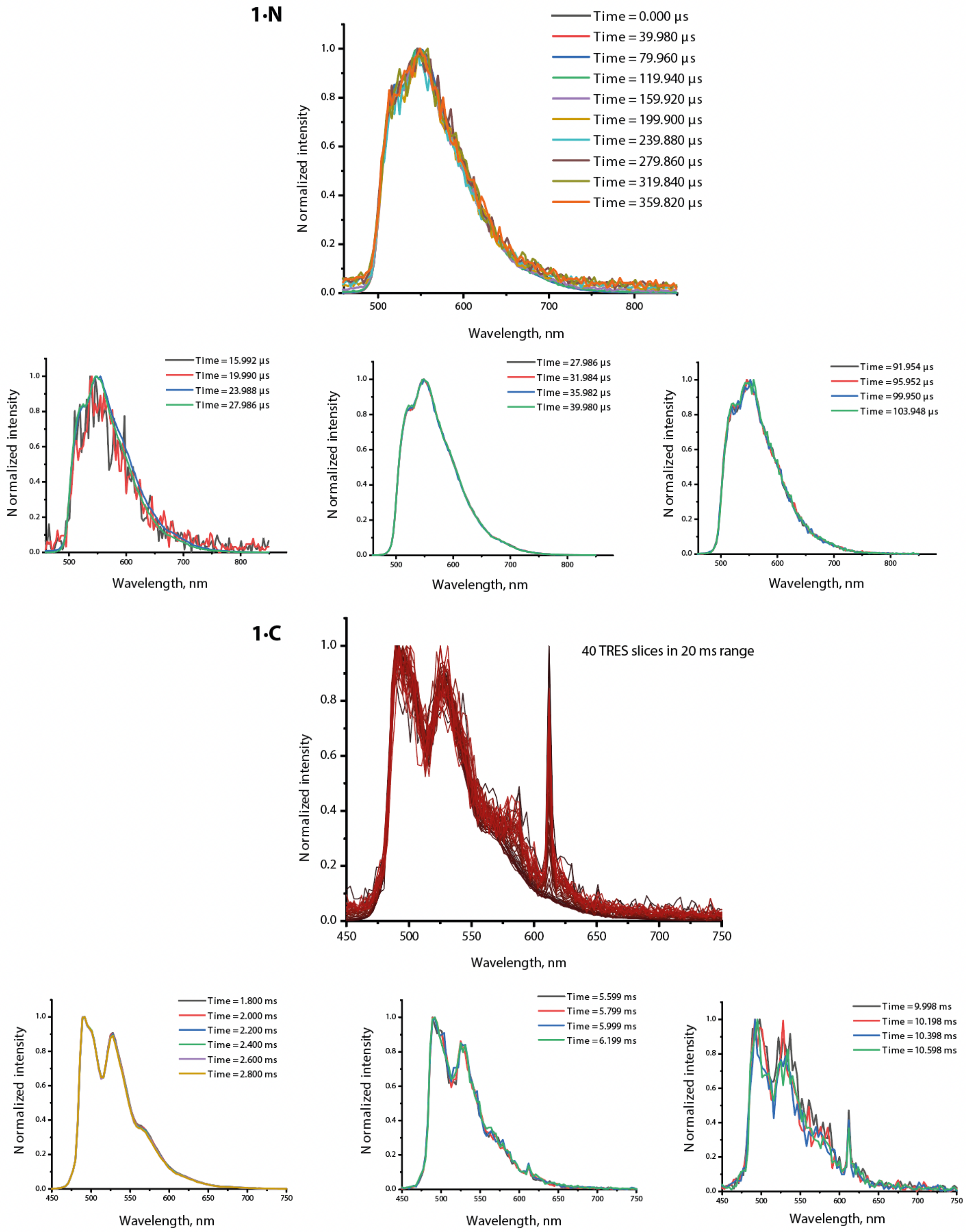

Figure S42. Slices of the TRES spectra of the **1·C** and **1·N**.

Table S9. Experimental and calculated T_1-state radiative lifetimes, singlet- and triplet-state spin–orbit coupling matrix elements, and admixture coefficients (δ_n) for **1·C**, **1·A**, and **1·N**.

	τ_{av}, exp, µs (77K)	τ_{av}, calc, µs	δ_n	$\langle S_n\|H_{SO}\|T_1\rangle$, cm^{-1}	$\langle T_n\|H_{SO}\|S_0\rangle$, cm^{-1}
**1·C**	2384.2	3990	0.063 (n=2)	410	87 (n=2)<br>80 (n=3)
**1·A**	138.7	305<br>(in-plane I^-)	0.031 (n=1)<br>0.071 (n=2)<br>0.0397 (n=3)	302<br>1097<br>1134	34 (n=2)<br>42 (n=3)
		31<br>(out-of-plane plane I^-)	0.222 (n=1)	552	24 (n=3)
**1·N**	89.8	222<br>(in-plane I^-)	0.042 (n=3)	1289	53 (n=2)
		13.6<br>(out-of-plane plane I^-)	0.278 (n=3)	1084	159 (n=2)

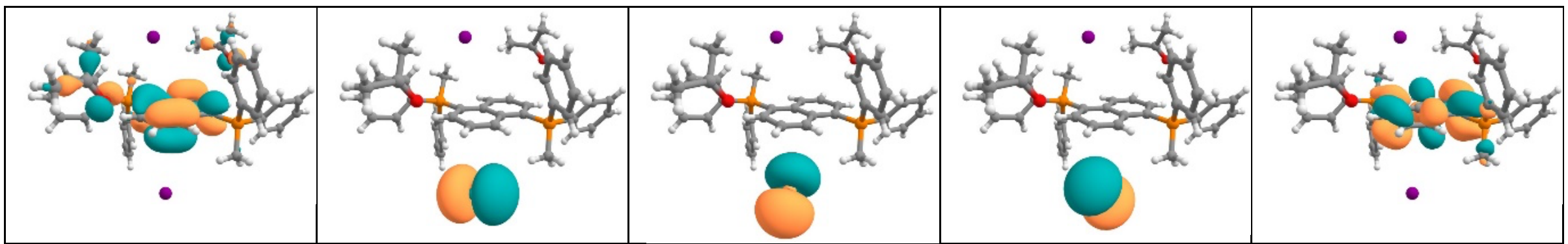

HOMO-7	HOMO-2	HOMO-1	HOMO	LUMO

HOMO-7→ LUMO (4%) LE
HOMO-2→ LUMO (2%) CT
HOMO-1→ LUMO (38%) CT
HOMO→ LUMO (50%) CT

Figure S43. Molecular orbitals corresponding to the T_1 excited state of the **1·A°** crystal structure fragment with out-of-plane orientation of iodide counterions calculated by wB97XD/DGDZVP method. The calculated energy of the T_1 state (578 nm) is in good agreement with the experimental phosphorescence spectrum (620 nm). The contribution of the CT configuration to the T_1 state is 90%, dominating the LE contribution (4%).

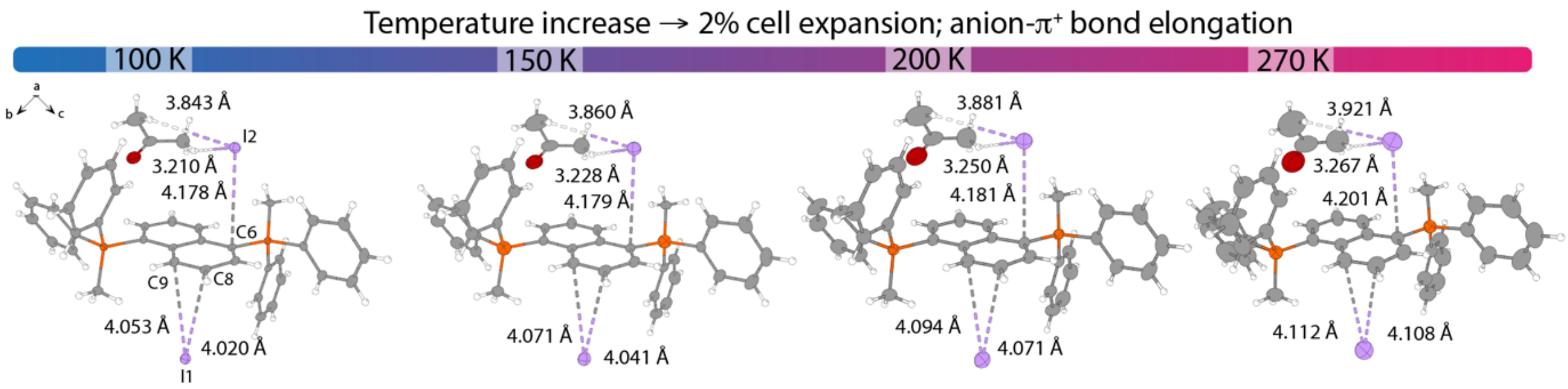

Figure S44. Variable temperature SCXRD measurements of the **1·A°**: molecular view of the **1·A°** with selected shortest I···C anion-π+ and I···H-C distances (displacement ellipsoids are shown at the 50% probability level).

Table S10. Summary of the variable temperature time-resolved measurements for **1·A°**.

Temperature [K]	τ (A$_i$) [μs]	τ$_{av}$ [μs][a]	Q.Y.	k$_r$ [s^{-1}]	k$_{nr}$ [s^{-1}]
77	6.80 (62.28 %)<br>9.20 (37.55 %)<br>51.78 (0.16 %)	7.8	0.71	9.1×10^4	3.7×10^4
117	5.46 (53.00 %)<br>7.76 (46.82 %)<br>41.56 (0.19 %)	6.6	–		
167	4.19 (53.58 %)<br>6.26 (46.30 %)<br>87.29 (0.11 %)	5.2	–		
217	2.59 (31.88 %)<br>4.45 (67.95 %)<br>25.71 (0.18 %)	3.9	–		
257	1.80 (22.59 %)<br>3.47 (76.99 %)<br>6.32 (0.43 %)	3.1	–		
297	2.12 (50.21 %)<br>3.16 (49.79 %)	2.6	0.21	8.1×10^4	3.0×10^5
317	1.18 (31.73 %)<br>2.16 (68.16 %)<br>16.70 (0.11 %)	1.9			

[a] Amplitude-weighted average lifetime, calculated from $\tau_{av}=\Sigma A_i\tau_i$, A_i=weight of the i-th exponent.

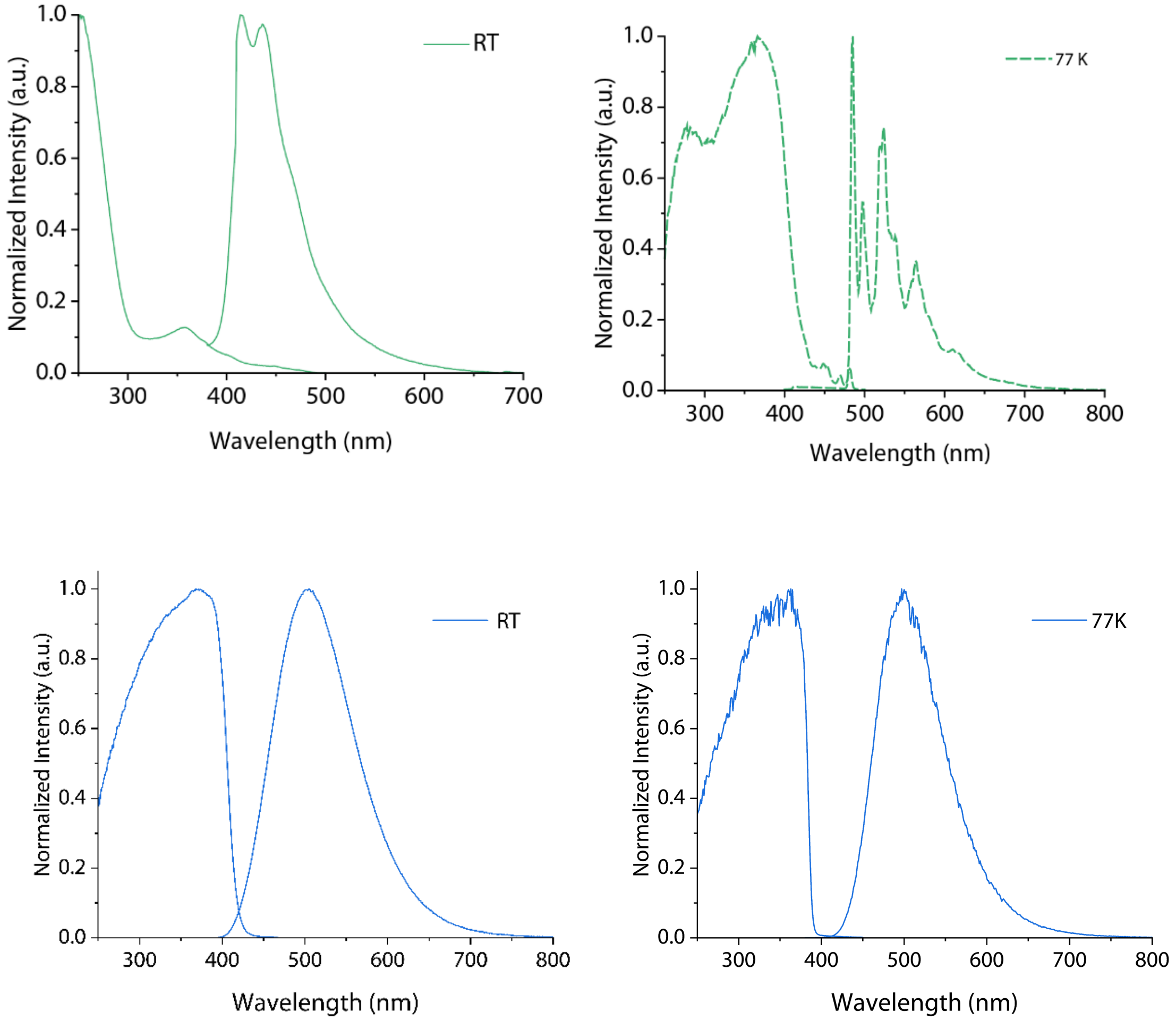

Figure S45. Excitation and emission profiles of **1·W** at 297 and 77 K.

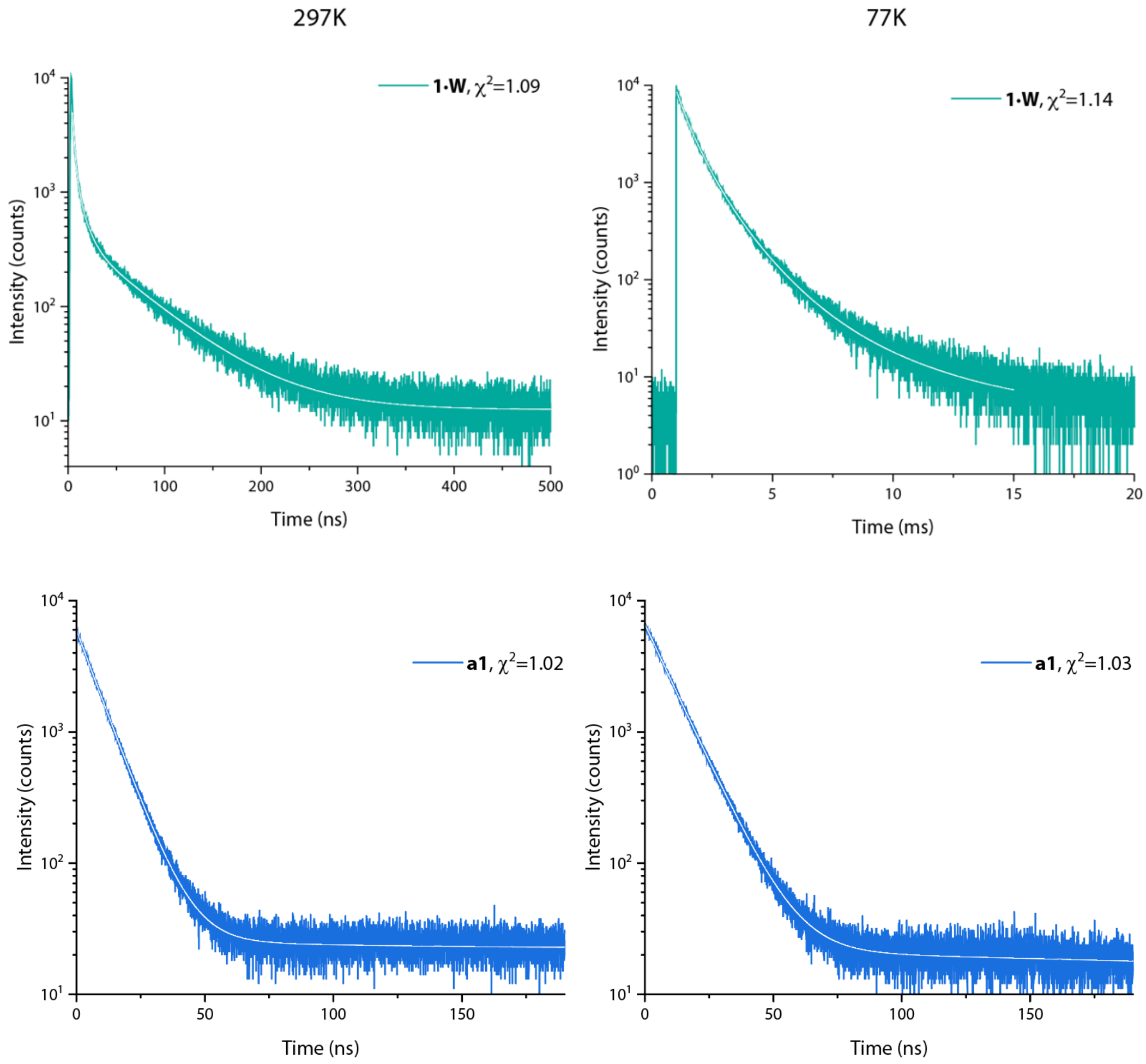

Figure S46. Time-resolved emission decays and their fit of solid **1·W** at 297 K and 77 K.

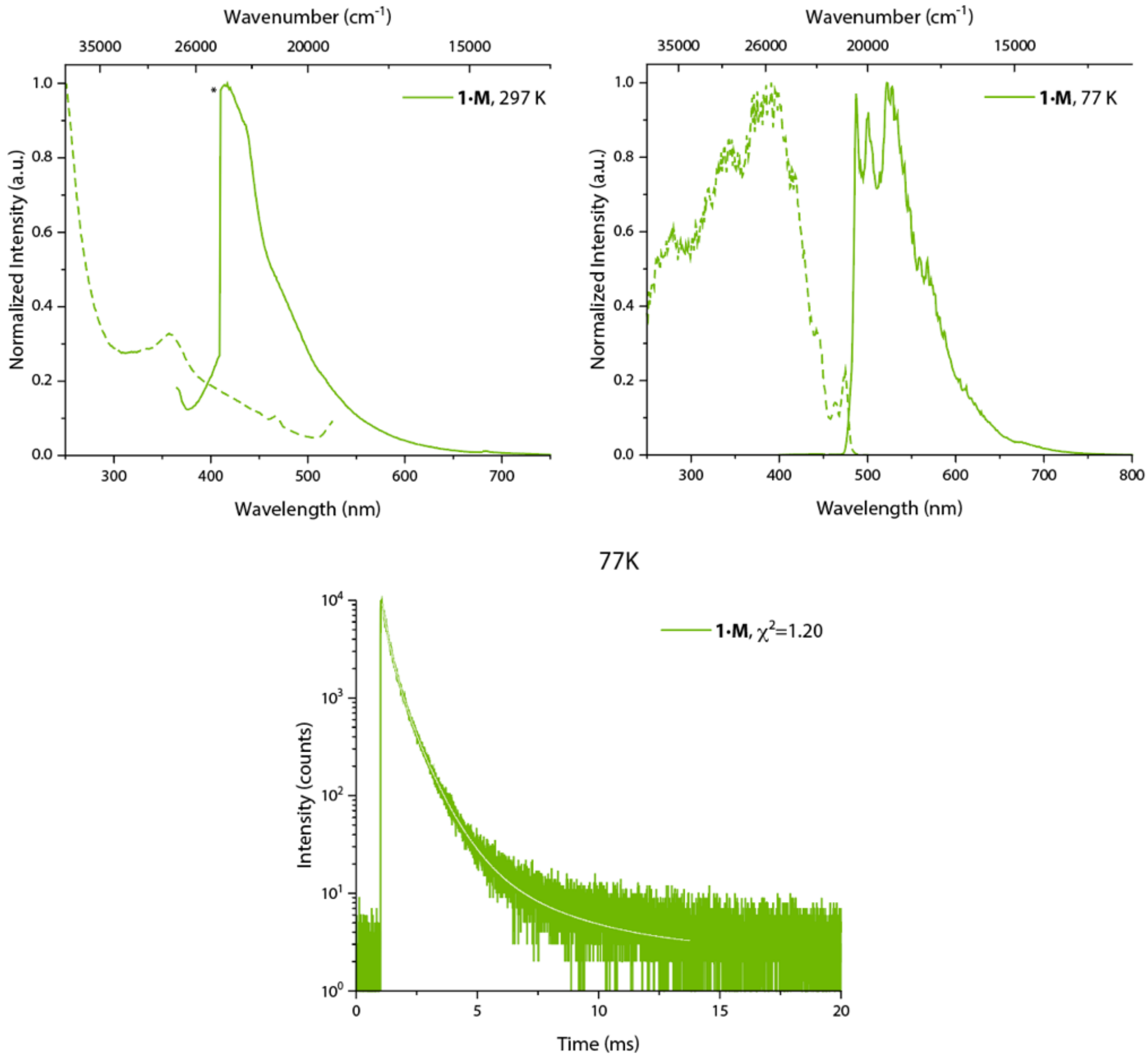

Figure S47. Excitation and emission profiles of **1·M** at 297 and 77 K. Time-resolved emission decays and their fit of solid **1·M** at 77 K. * – sample appears to be poorly emissive at 297 K, which resulted in the appearance of a reproducible artifact of the detector at 410 nm.

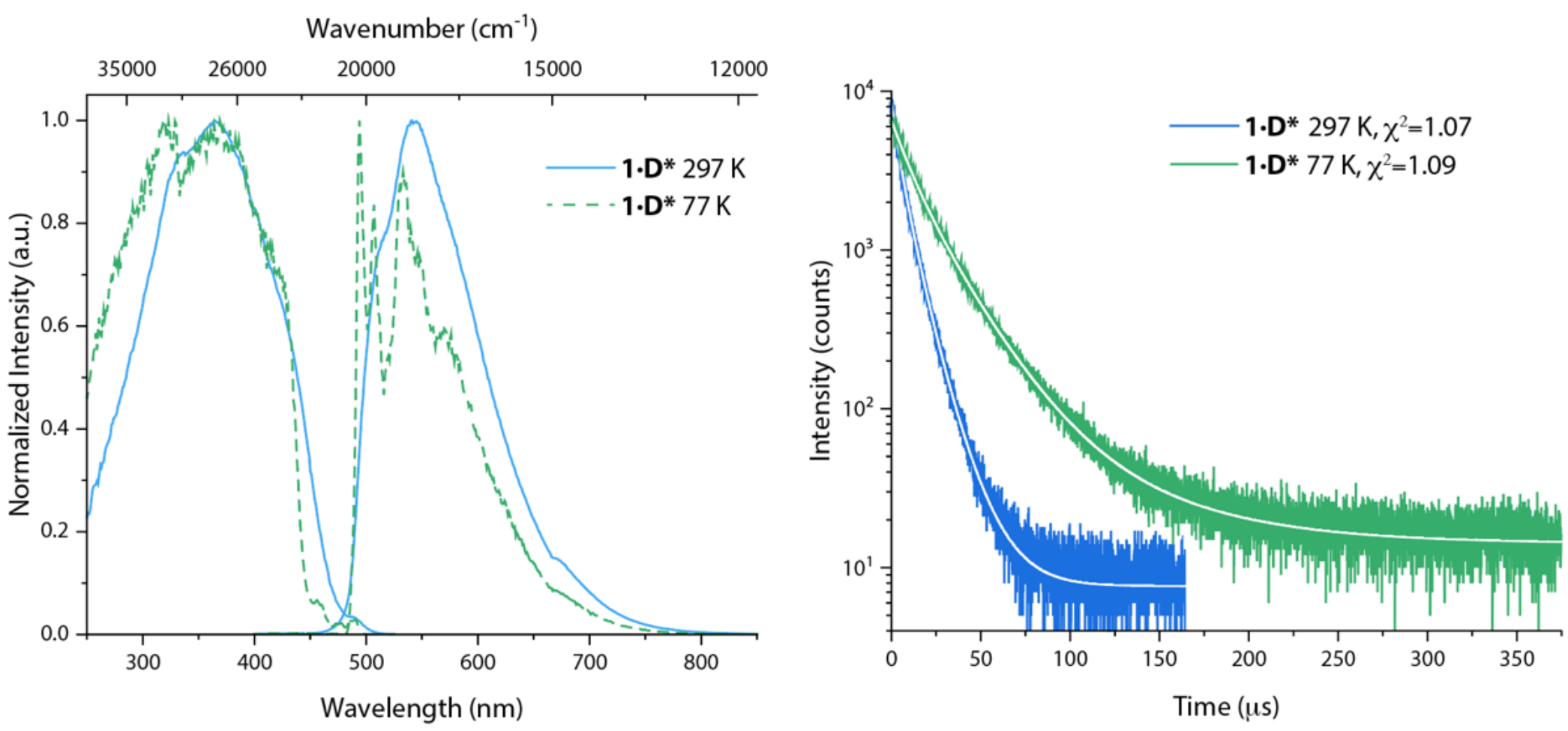

Figure S48. Excitation and emission profiles of **1·D*** at 297 and 77 K. Time-resolved emission decays and their fit of solid **1·D*** at 297 K and 77 K.

References

[1] A. Belyaev, I. Kolesnikov, A. S. Melnikov, V. V. Gurzhiy, S. P. Tunik, I. O. Koshevoy, "Solution *versus* solid-state dual emission of the Au(i)-alkynyl diphosphine complexes *via* modification of polyaromatic spacers" *New Journal of Chemistry* **2019**, DOI 10.1039/C9NJ03426A.

[2] G. M. Sheldrick, "SHELXT – Integrated space-group and crystal-structure determination" *Acta Cryst A Foundations and Advances* **2015**, *71*, 3–8.

[3] G. M. Sheldrick, IUCr, "Crystal structure refinement with *SHELXL*" *Acta Crystallogr. C Struct. Chem.* **2015**, *71*, 3–8.

[4] T. Delouche, A. Vacher, E. Caytan, T. Roisnel, B. Le Guennic, D. Jacquemin, M. Hissler, P. A. Bouit, "Multi-Stage Redox Systems Based on Dicationic P-Containing Polycyclic Aromatic Hydrocarbons" *Chemistry – A European Journal* **2020**, *26*, 8226–8229.

[5] R. F. W. . Bader, "Atoms in molecules : a quantum theory" **1990**, 438.

[6] A. D. Becke, "Density-functional thermochemistry. III. The role of exact exchange" *J. Chem. Phys.* **1993**, *98*, 5648–5652.

[7] C. Lee, W. Yang, R. G. Parr, "Development of the Colle-Salvetti correlation-energy formula into a functional of the electron density" *Phys. Rev. B* **1988**, *37*, 785.

[8] N. Godbout, D. R. Salahub', J. Andzelm' And, E. Wimmer, "Optimization of Gaussian-type basis sets for local spin density functional calculations. Part I. Boron through neon, optimization technique and validation" *https://doi.org/10.1139/v92-079* **2011**, *70*, 560–571.

[9] E. Espinosa, E. Molins, C. Lecomte, "Hydrogen bond strengths revealed by topological analyses of experimentally observed electron densities" *Chem. Phys. Lett.* **1998**, *285*, 170–173.

[10] J. Da Chai, M. Head-Gordon, "Long-range corrected hybrid density functionals with damped atom–atom dispersion corrections" *Physical Chemistry Chemical Physics* **2008**, *10*, 6615–6620.

[11] E. Van Lenthe, E. J. Baerends, J. G. Snijders, "Relativistic regular two-component Hamiltonians" *J. Chem. Phys.* **1993**, *99*, 4597–4610.

[12] E. Van Lenthe, J. G. Snijders, E. J. Baerends, "The zero-order regular approximation for relativistic effects: The effect of spin–orbit coupling in closed shell molecules" *J. Chem. Phys.* **1996**, *105*, 6505–6516.

[13] E. J. Baerends, N. F. Aguirre, N. D. Austin, J. Autschbach, F. M. Bickelhaupt, R. Bulo, C. Cappelli, A. C. T. van Duin, F. Egidi, C. Fonseca Guerra, A. Förster, M. Franchini, T. P. M. Goumans, T. Heine, M. Hellström, C. R. Jacob, L. Jensen, M. Krykunov, E. van Lenthe, A. Michalak, M. M. Mitoraj, J. Neugebauer, V. P. Nicu, P. Philipsen, H. Ramanantoanina, R. Rüger, G. Schreckenbach, M. Stener, M. Swart, J. M. Thijssen, T. Trnka, L. Visscher, A. Yakovlev, S. van Gisbergen, "The Amsterdam Modeling Suite" *Journal of Chemical Physics* **2025**, *162*, 162501.
